# Stabilized thioamide peptide agonists of the neuropeptide Y type 2 receptor for targeted cancer imaging

**DOI:** 10.1039/d6cb00042h

**Published:** 2026-06-12

**Authors:** Hoang Anh T. Phan, Yanan Chang, Taylor M. Barrett, Kristen E. Fiore, Daniel Y. Zhang, Ethan J. Grove, E. James Petersson

**Affiliations:** a Department of Chemistry, School of Arts and Sciences, University of Pennsylvania 231 South 34th Street Philadelphia PA 19104 USA ejpetersson@sas.upenn.edu; b Department of Biochemistry and Biophysics, Perelman School of Medicine, University of Pennsylvania 421 Curie Boulevard Philadelphia PA 19104 USA

## Abstract

The neuropeptide Y type 2 receptor (Y_2_R) is highly expressed in human neuroblastoma and glioblastoma cells, and it has been shown to stimulate cancer cell growth. To develop an effective imaging probe for glioblastomas, peptide-based agents can be designed as Y_2_R agonists to be internalized by receptor-mediated endocytosis. However, the short half-life of most neuropeptides (<30 minutes) makes them unsuitable as imaging probes. Thioamide substitution, a single-atom O-to-S modification, is a promising tool to enhance peptide stability for therapeutic and imaging purposes. Herein, we designed and evaluated the first fluorescent cyclic thioamide peptides (HAP1 and HAP1-R^S^_33_) as specific agonists and imaging agents of the Y_2_R. High-performance liquid chromatography and mass spectrometry were used to identify cleavage sites by analyzing peptides after incubating in mouse serum, confirming enhanced stability of the peptides. Our stabilized cyclized thiopeptide probe showed a significant improvement in half-life from approximately 30 minutes to over 8 hours while maintaining moderate potency and high selectivity for binding with Y_2_R receptor expressing cells, enabling selective imaging of Y_2_R-expressing neuroblastoma cells. These results show that thioamide stabilized cyclized peptide probes targeting specific receptors may have potential for use in different biological or clinical applications.

## Abstract 2

A fluorescent cyclic thioamide peptide has a serum half life over 8 hours and high selectivity for binding to Y_2_R receptor expressing cells, enabling imaging of neuroblastomas and glioblastomas.

## Graphical abstract

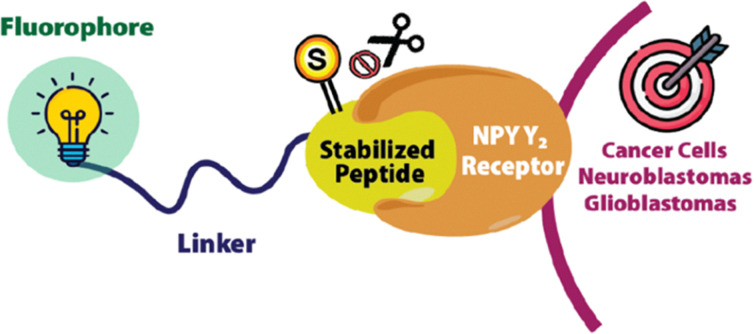

## Introduction

**Fig. 1:**
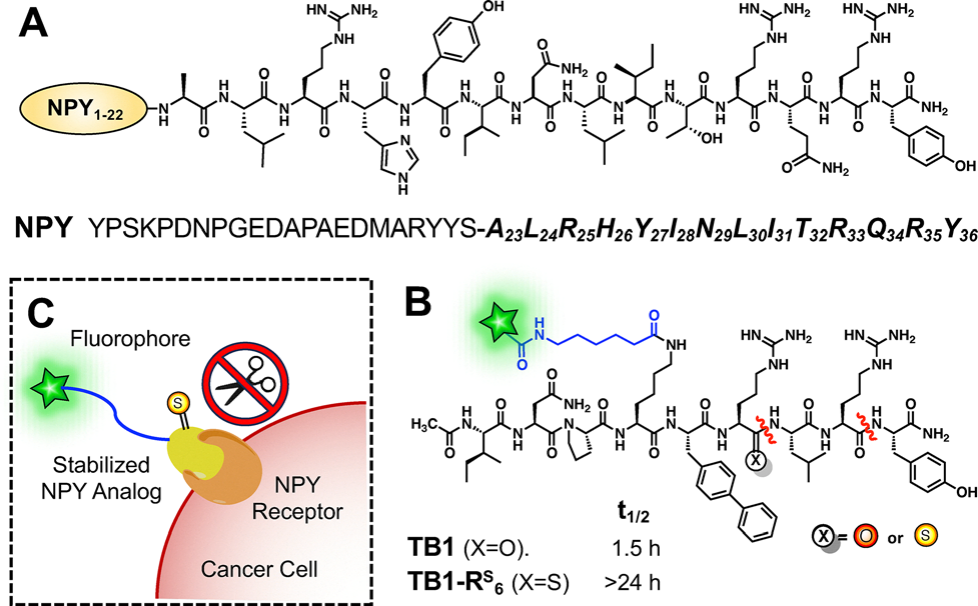
Schematic of Y_2_R probe design. (A) NPY C-terminal sequence and structure. (B) Y_1_R imaging probe TB1-R^S^_6_, showing thioamide effects on kallikrein proteolysis and serum half-life compared to the all-amide analog TB1 (based on BVD-15). (C) The thioamide stabilized peptide is conjugated to a linker (blue) and a fluorophore. The peptide targets the NPY receptor that is overexpressed in cancers such as Y_2_R in neuroblastomas and glioblastomas, making it an imaging agent for these cancers.

Neuropeptide Y (NPY, Fig. 1A) receptors, with five different subtypes (Y_1_, Y_2_, Y_4_, Y_5_, and y_6_) identified in mammals, belong to the superfamily of G-protein coupled receptors (GPCRs).^1–3^ While the y_6_ receptor is only functional in rabbits and mice, the other four receptors, Y_1_, Y_2_, Y_4_, and Y_5_, have been known to play many critical physiological roles in human metabolic homeostasis, memory, anxiety, cognition, and circadian rhythm.^2–5^ NPY receptors have thus been implicated in many diseases such as metabolic disorders, hypertension, neurodegenerative diseases, and cancers.^4–6^ The native ligands for NPY receptors are the 36-amino acid NPY, along with two of its relatives – pancreatic polypeptide (PP) and peptide YY (PYY).^5^ Owing to the specific interactions between NPY and its receptors, NPY-mimetic peptides offer promising avenues for the design of scaffolds specifically targeting NPY receptors for therapeutic and imaging purposes.

The most appealing targets for NPY-based therapeutics and imaging so far are the Y_1_ and Y_2_ receptors (Y_1_R and Y_2_R), the two most well-studied NPY receptors. Y_1_R and Y_2_R have been found to be overexpressed in many types of cancers, such as breast carcinomas, ovarian cancers, and brain cancers.^4,5,7^ In the case of breast cancer, Y_1_R overexpression was observed in 85% to 100% of incidences of primary human breast cancers and metastatic tumors originating from breast tumors, whereas normal human breast tissues predominantly express Y_2_R.^4,8^ This differentiation between Y_1_R : Y_2_R expression in normal and cancerous breast tissues has motivated development of diagnostic and therapeutic tools for breast cancer imaging and treatment. This is a challenging endeavor since Y_1_R and Y_2_R have similar pharmacological profiles; both can bind to NPY and PYY with equally high affinity, but have low affinity towards PP.^1,3,9^

NPY-based radiolabeled peptidyl scaffolds such as BVD-15 have been subsequently designed to image breast cancers overexpressing Y_1_Rs.^10,11^ On the other hand, according to Human Protein Atlas database and their consensus dataset for RNA expression, Y_2_R is mostly expressed in brain tissues.^4,10–14^ Furthermore, a comprehensive study investigating the expression of NPY receptors in 131 primary human brain tumors showed that glioblastomas predominantly expressed Y_2_R over Y_1_R.^14–16^ Beyond high incidence or frequency of Y_2_R enrichment, these glioblastomas also exhibited remarkably high measured densities of Y_2_R receptor; these densities were among the highest NPY receptor densities in any tissues, even comparable to that of Y_1_R in breast cancers.^15^ Additionally, Y_2_R is also a potential target for neuroblastoma as it is expressed in neuroblastoma cell lines and tissues.^17–19^ Given these precedents and our laboratory's previous success in targeting Y_1_R for breast cancer imaging, summarized below, we sought to investigate NPY analogs targeting Y_2_R as potential intraoperative and diagnostic agents for glio- and neuroblastomas.^20^

The overarching goal of our study is to utilize thioamide substitution to design stable, fluorescently labeled Y_2_R-targeting peptides. We design fluorescent probes because they have been shown to help improve surgical outcomes by guiding surgeons to identify margins and precisely remove tumor during resection.^21^ Locally disrupted blood–brain barrier may further enhance fluorescent tracer accumulation at the site of the target tumor tissue. To overcome proteolytic stability issues of imaging peptides, we previously described the design of a thioamide modified peptide, TB1-R^S^_6_ (Fig. 1B, thioamide denoted by superscript S), which targets Y_1_R and has the potential to be used as a breast cancer imaging probe.^20^

TB1-R^S^_6_ was significantly stabilized toward proteolytic degradation compared to its all-amide congener, without sacrificing bioactivity. Studies of thioamide effects in model peptide substrates of serine proteases determined which thioamide positions would disrupt proteolysis. In particular, we found that the kallikrein protease, known to cleave at two Arg residues in the C-terminus of NPY, could be disrupted by placement of a thioamide at either the P3 or P1 position. Thus, we used a single thioamide substitution to block cleavage at both Arg sites since it would be in the P1 position relative to Arg_33_ and the P3 position relative to Arg_35_ (Fig. 1B).

We envision that a similar approach can be taken to design NPY-inspired peptides specifically targeting Y_2_R for imaging of glioblastomas and neuroblastomas. Additionally, thioamidation has been previously shown to significantly improve the affinity and pharmacological properties of macrocyclic peptides, by enhancing metabolic stability and cell permeability.^22,23^ In this work, we developed the first fluorescently labeled, cyclic thioamide stabilized peptides that have specific agonistic properties towards Y_2_R, thereby serving as a selective imaging agent for neuro- and glioblastoma imaging. The concept of our Y_2_R-targeting probe design is summarized in Fig. 1C.

## Results & discussion

### Fluorescently labeled NPY Y2 receptor targeting peptide design and serum stability

Researchers have developed small molecule Y_2_R antagonists such as BIIE0246 and JNJ-5207787.^24,25^ A Y_2_R-targeted imaging effort was led by Winterdahl *et al.*, where they developed a radioligand for positron emission tomography (PET) imaging based on the small molecule antagonist JNJ-31020028, thereby providing the first images of Y_2_Rs in the living brain.^26^ However, it is challenging to attach a fluorophore to small molecule antagonists without compromising their binding since they are often of comparable size. In terms of peptide-based agents, although full-length NPY and PYY peptides are the natural ligands of Y_2_R, they do not offer specificity toward Y_2_R over Y_1_R. Furthermore, a labeled peptide of 34–36 amino acids in length can be cumbersome to synthesize and is labile to proteolysis and other undesired metabolic modifications. To this end, several Y_2_R-specific peptides have been developed, with the smallest Y_2_R full agonist being a dodecapeptide cyclic peptide.^18,27–29^

For our design, evidence from the literature supports the idea that cyclic NPY derivatives, featuring a lactam bridge, can potentially offer enhanced activity and selectivity toward Y_2_R over Y_1_R compared to their linear counterparts, motivating us to pursue 12–13 amino acid cyclic peptides that are based on the C-terminus of NPY (Fig. 1A).^18,30–32^ Furthermore, we chose this as a starting scaffold because of its agonistic property. While antagonists like Y_1_R targeting TB1 scaffolds can bind on the cell surface expressing NPY receptors and thus are suitable for imaging, agonists are in fact preferred for imaging.^14,33,34^ Agonists can be internalized by receptor-mediated endocytosis, thereby allowing them to selectively accumulate inside tumor cells.^33,34^

In our design, placement of the fluorophore and cyclization sites need to be strategic since we do not want to disrupt Y_2_R binding and selectivity. A complete alanine scan by Beck-Sickinger *et al.* showed that Tyr_27_, Asn_29_, Ile_31_, Arg_33_, Gln_34_, Arg_35_, and Tyr_36_ are critical for Y_2_R binding.^35^ Furthermore, C-terminal amino acids have been identified to form important interactions with the negatively charged residues in the Y_2_R binding pocket.^36,37^ On the other hand, residues in the N-terminal region of the NPY fragment, Ile_28_, Leu_30_, and Thr_32_, seem to be ideal positions for modifications. We thus decided to conjugate our fluorophore (*i.e.*, fluorescein) with a linker at the N-terminus of the peptides. As for the cyclization site, we chose Lys_28_ and Glu_32_ based on literature reports by Beck-Sickinger, Rist, and colleagues.^18^ The acetylated dodecapeptide peptide, cyclic [Lys_28_-Glu_32_]NPY_25–36_ (Table 1), was in fact the first small full agonist of the Y_2_R, and this peptide was validated by a receptor binding study with the Y_2_R-expressing human neuroblastoma cells SMS-KAN.^18^

**Table 1:** Fluorescently labeled peptides targeting the Y_2_R and control peptides

Peptide	Sequence
Oxo Linear_24–36_ (1)	Ac-K*RHYKNLIERQRY-NH_2_
Thio Linear_24–36_ (2)	Ac-K*RHYKNLIE**R**^S^QRY-NH_2_
Oxo Cyclic_24–36_ (3)	Ac-K*RHYK̲K̲N̲L̲I̲RQRY-NH_2_
Thio Cyclic_24–36_ (4)	Ac-K*RHYK̲K̲N̲L̲I̲R^S^QRY-NH_2_
HAP1 (5)	Ac-K*HYK̲K̲N̲L̲I̲RQRY-NH_2_
HAP1-R^S^_33_ (6)	Ac-K*HYK̲K̲N̲L̲I̲**R**^S^QRY-NH_2_
NPY (7)	YPSKPDNPGEDAPAEDMARYYSA*LRHYINLITRQRY*
NPY[L_28,31_]_24–36_ (8)	Ac-LRHYLNLLTRQRY
PYY (9)	YPIKPEAPGEDASPEELNRYYAS*LRHYLNLVTRQRY*

Although backbone modifications have been made previously in the C-terminus of related peptide YY,^1,2^ the use of thioamide stabilization here with its two site effects, and its application in the cyclic peptide and imaging framework, are novel, to our knowledge. Since this design shares the C-terminal RXRY (where *X* is variable) sequence with TB1-R^S^_6_, we hypothesized that an unmodified peptide would have similar proteolytic liabilities and that insertion of a thioamide at Arg_33_ could exert the same stabilizing effects. Considering that Arg_35_ has been shown to be critical for Y_2_R binding further cemented our choice of Arg_33_ as the position for thioamidation.^38^ Thus, we synthesized a series of fluorescently labeled peptides as summarized in Table 1 and investigated them through serum stability assays, biological activity assays, and imaging experiments. The synthesis strategies and analytical data are included in the SI (Fig. S1 and S2; Tables S1–S3). As an example, we also characterized one construct (HAP1; peptide 5) to confirm if its lack of significant secondary structure through circular dichroism (CD) and nuclear magnetic resonance (NMR) (Fig. S27 and S28).

**Fig. 2:**
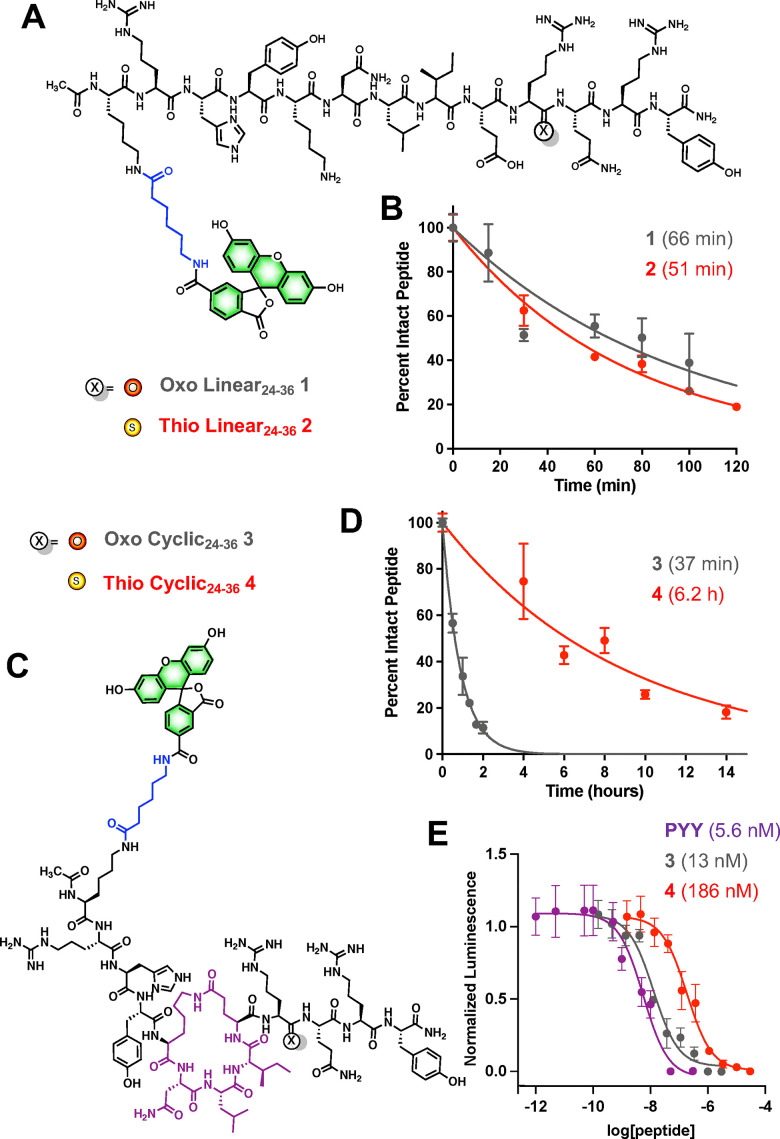
Serum stability of linear and cyclic Ac-NPY_24–36_. Peptides. (A) Structure of the all-amide and thioamide linear peptides, Ac[K_24,28_E_32_]NPY_24–36_ (peptides 1 and 2). (B) Mouse serum stability of peptides 1 and 2. Standard deviations for 3 technical replicates are shown. (C) Structure of the all-amide and thioamide cyclic peptide, Ac-Cyclo_28–32_[K_24,28_E_32_]NPY_24–36_ (peptides 3 and 4). (D) Mouse serum stability of the peptides shown in (C). Peptides were incubated in mouse serum and the resulting mixtures were analyzed with HPLC and MALDI MS to quantify intact peptide at each time points as wells as cleavage sites. Standard errors with at least 2 biological replicates with 3 technical replicates each are shown. Primary data are shown in the SI (Fig. S3–S6). (E) Y_2_R activation (agonist assays): both peptides can serve as good agonists of the Y_2_R. Standard errors with 1-2 biological replicates with 3 technical replicates each are shown.

A fluorescently-labeled linear, all-amide NPY Y_2_R peptide and its thioamide counterpart (Oxo Linear_24–36_ (1) and Thio Linear_24–36_ (2); Table 1, Fig. 2A) were first synthesized along with their cyclic all-amide and thioamide counterparts (Oxo Cyclic_24–36_ (3) and Thio Cyclic_24–36_ (4); Table 1 and Fig. 2C). We were interested in comparing the thioamide effects in linear peptides *versus* cyclic peptides of the same sequence. Mouse serum stability assays and assays with purified kallikrein using high performance liquid chromatography (HPLC) and matrix-assisted laser desorption ionization mass spectrometry (MALDI MS) indicated that thioamide substitution did not enhance the overall stability of the linear peptides due to multiple cleavage sites by proteases other than kallikrein in mouse serum and at sites other than the thioamidated Arg^S^_33_ (Fig. S3, S4, S10 and S11). Both all-amide and thioamide linear peptides had a half-life (*t*_1/2_) of 50–70 minutes (Fig. 2B). Interestingly, cyclization of the peptide alone did not help with the peptide stability against kallikrein (Fig. S12) and mouse serum proteases (Fig. 2D and Fig. S5), as the half-life of Oxo Cyclic_24–36_3 was only 37 minutes, which was even shorter than the half-life of Oxo Linear_24–36_1 (*t*_1/2_ = 66 minutes). This somewhat surprising result can be explained by considering the multiple cleavage sites in the peptide. Kallikrein cleaves Oxo Linear_24–36_1 primarily at Lys_28_ and Arg_35_ (Fig. S10). Cyclization at Lys_28_ prevents cleavage there, but Arg_25_ then becomes one of the primary cleavage sites (Fig. S12).

**Fig. 3:**
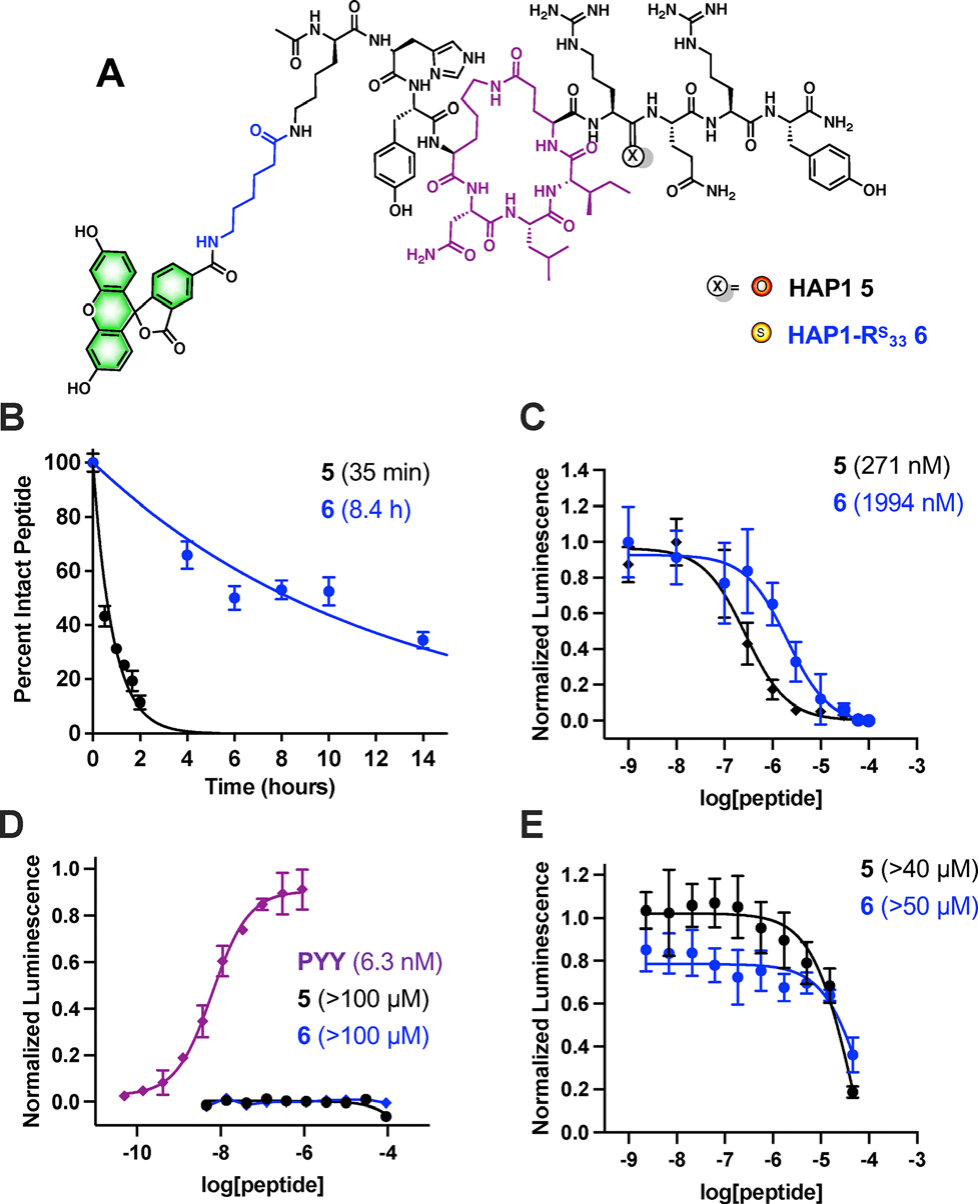
Serum stability and receptor binding data of HAP1 and HAP1-R^S^_33_. (A) Structure of the all-amide and thioamide cyclic peptide, Ac[K_25,28_E_32_]NPY_25–36_ peptides (5 and 6). (B) Mouse serum stability of the peptides. Peptides were incubated in mouse serum and the resulting mixtures were analyzed with HPLC and MALDI MS to quantify intact peptide at each time points as wells as cleavage sites. Standard deviations of 3 technical replicates are shown. Primary data are shown in Fig. S7 and S8. (C) NPY Y_2_R activation (agonist assays): both peptides can serve as good agonists of the Y_2_R. (D) NPY Y_1_R activation (agonist assays): HAP1 and HAP1-R^S^_33_ are not agonists of Y_1_R; PYY, a known agonist of Y_1_R, served as a control. (E) NPY Y_1_R antagonist assays: HAP1 and HAP1-R^S^_33_ showed very weak antagonist properties towards Y_1_R. Dose response curves for HAP1 and HAP1-R^S^_33_ are shown for all biological activity assays. All data points were done in at least triplicates across 1-3 biological replicates (standard errors are shown).

Placing a thioamide at Arg_33_ significantly protected Thio Cyclic_24–36_ (4) against kallikrein (Fig. S13; at 30 minutes, most of the HAP1 was cleaved by kallikrein at Arg_35_, but most of HAP1-R^S^_33_ was still intact) and increased the stability of the cyclic peptide in mouse serum (*t*_1/2_ = 6.2 hours; Fig. 2D and Fig. S6), which was more than 10 times that of the all-amide peptides 1 and 2. This validated our decision to move forward with a cyclic scaffold rather than a linear one for this study and demonstrated that both cyclization and thioamidation were necessary for global peptide stability. Nevertheless, the stability of this cyclic thioamide Y_2_R peptide was still low compared to the Y_1_R-targeting homolog (TB1-R^S^_6_) that we developed previously (*t*_1/2_ ≥24 hours). HPLC and MALDI MS data from serum stability assays showed that the thioamide cyclic peptide got cleaved at the following positions Ac-K(Ahx-FAM)/R/HY/K̲K̲N̲L̲I̲R^S^QR/Y-NH_2_ (Fig. S6), prompting us to further optimize this peptide. As the literature suggests that the N-terminus of the NPY peptide is not critical in binding and recognition by Y_2_R, we removed Arg_25_ to eliminate a kallikrein cleavage site (Fig. S15) and make a slightly shortened scaffold (HAP1 or peptide 5; Table 1, Fig. 3A). Both the all-amide (HAP1) and thioamide (HAP1-R^S^_33_) versions of this peptide had fewer cleavage sites in mouse serum than that of the NPY_24–36_ peptides 1-4: Ac-K(Ahx-FAM)HY/K̲K̲N̲L̲I̲R^S^QR/Y-NH_2_ (Fig. S8). The all-amide peptide HAP1 (5) still had a short half-life of 35 minutes (due primarily to cleavage at Arg_35_), while its thioamide HAP1-R^S^_33_ (6) analog had a 14-fold greater half-life of 8.4 hours (Fig. 3B). We proceeded to further test the HAP1 and HAP1-R^S^_33_ with biological activity and selectivity assays, as well as imaging and flow cytometry experiments.

### Evaluation of biological activity

We proceeded to evaluate the biological activity of the original Oxo Cyclic_24–36_ and Thio Cyclic_24–36_ (peptides 3 and 4) along with HAP and HAP1-R^S^_33_ peptides (peptides 5 and 6) with commercial enzyme-linked luminescence assays. Experimental setup and details are included in the SI. We investigated the agonistic property of our peptides with CHO-K1 cells expressing Y_2_R with various doses of the peptides. We found that both HAP1 and HAP1-R^S^_33_ could effectively activate Y_2_R (HAP1: EC_50_ = 271 ± 40 nM; HAP1-R^S^_33_: EC_50_ = 1994 ± 477 nM) (Fig. 3C and Table 2). PYY (a close analog of NPY and a known agonist of both the Y_1_R and Y_2_R) and NPY[Leu_28,31_]_24–36_ (a known selective agonist of the Y_2_R) were used as positive controls and we observed EC_50_ values that were consistent with the literature values, validating our assays (Table 2).

**Table 2:** Y_1_R and Y_2_R potency of peptides

Peptide	Y_2_R EC_50_ (nM) (Agonist)	Y_1_R EC_50_ (µM)	
		Agonist	Antagonist
Oxo Cyclic_24–36_ (3)	13 ± 2	—	—
Thio Cyclic_24–36_ (4)	186 ± 26	—	—
HAP1 (5)	271 ± 40	None	>40 µM
HAP1-R^S^_33_ (6)	1994 ± 477	None	>50 µM
NPY[L_28,31_]_24–36_ (8)	25 ± 2	—	—
PYY (9)	5.6 ± 1.0	6.3 ± 0.7 nM	—

It is clear that the modifications made to enhance the stability of the HAP1 constructs compromised some Y_2_R activity. Our first cyclic peptide construct, Oxo Cyclic_24–36_ (3), despite having a linker and a fluorophore, had an EC_50_ value of 13 ± 2 nM, which was very similar to that of the control linear peptide NPY[Leu_28,31_]_24–36_ (8). It was only when we deleted Arg_25_ to make the oxo cyclic HAP1 (5) that the EC_50_ became 271 nM (a 21-fold decrease in potency compared to 3), which is consistent with previous literature showing that Arg_25_ interactions contribute to receptor binding, although not necessarily activation.^37,39,40^ HAP1-R^S^_33_ (6) had a further 7-fold decrease in Y_2_R potency compared to all-amide HAP1 (5). Interestingly, there was a larger decrease in activity upon Arg_25_ deletion for the oxo peptides (21-fold for 3*vs.*5) than for the thio peptides (11-fold for 4*vs.*6), suggesting that the loss of affinity or activity upon Arg_25_ deletion is partially rescued by thioamidation. Given that thioamidation improved the half-life of the peptide 14-fold and that many Arg_33_ modification attempts in the literature have resulted in between 70 and more than 500-fold decreases in Y_2_R potency, our modification is a reasonable compromise and a good starting point for further optimization.^41^ It should also be noted that since our goal is to develop imaging agents, not therapeutics, weak receptor activation is not necessarily a problem as long as binding is maintained.

To evaluate the NPY receptor subtype specificity of these peptides, we conducted similar assays using CHO-K1 cells expressing the Y_1_R with various doses of HAP1 and HAP1-R^S^_33_ peptides. Both peptides exhibited no agonistic properties toward Y_1_R (Fig. 3D, Table 2). PYY (peptide 9) was again used as a control since it is an agonist of both the Y_1_R and Y_2_R; our measured EC_50_ value for PYY towards Y_1_R was 6.3 nM, consistent with the literature value of 4.1 nM (Y_1_R CHO-K1 β-Arrestin GPCR agonist assay) (Fig. 3D). We also attempted to conduct an antagonist assay with CHO-K1 cells expressing Y_1_R in which we activated the receptor using PYY and tested the inhibition of this activation with different concentrations of our HAP1 and HAP1-R^S^_33_ peptides. The peptides only elicit weak antagonistic responses toward Y_1_R in the µM range (Fig. 3E, Table 2). In other words, we demonstrated that both our all-amide and thioamide peptides showed selectivity towards Y_2_R over Y_1_R, and the cyclization and thioamide modifications enhance these imaging peptides’ stability and Y_2_R selectivity.

### Cellular imaging and flow cytometry

**Fig. 4:**
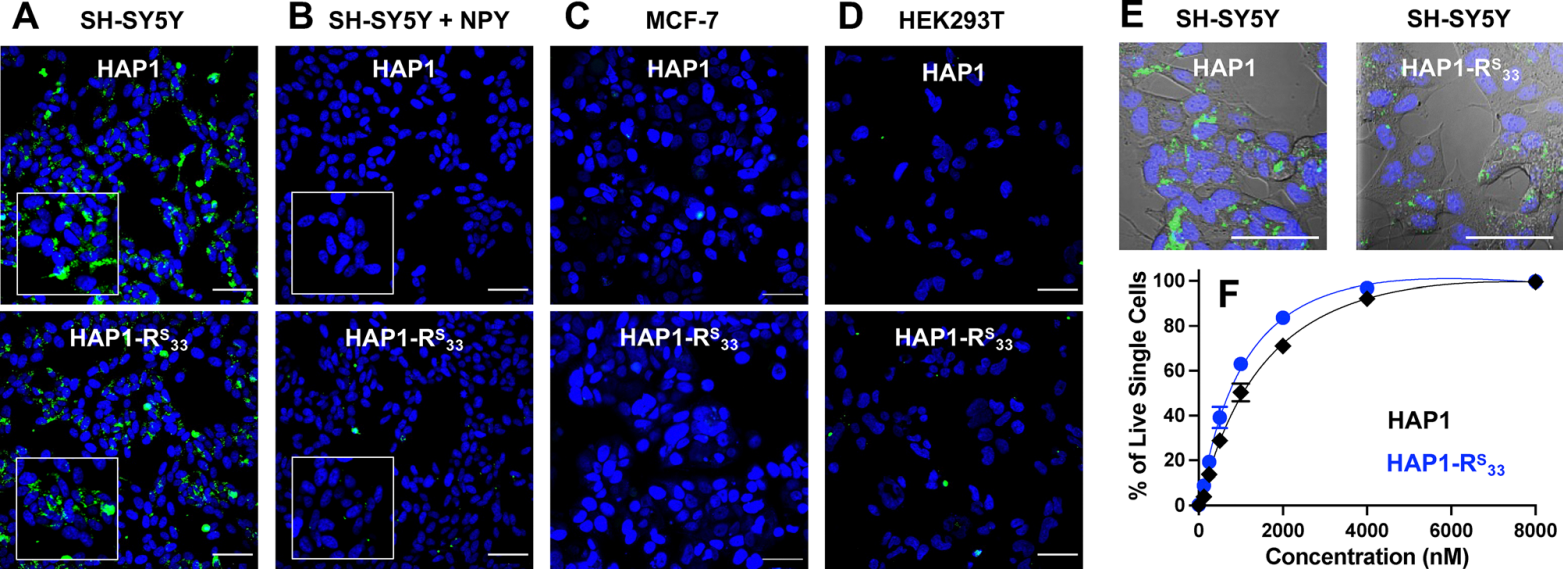
Selective binding of HAP1 and HAP1-R^S^_33_ to Y_2_R-expressing cancer cells. Imaging (A and B) neuroblastoma SH-SY5Y cells (Y_2_R-expressing cells), (C) breast cancer MCF-7 cells (Y_1_R- expressing cells), and (D) HEK293T (no NPYR) cells with all-amide HAP1 and thioamide HAP1-R^S^_33_ peptides (green fluorescence). Hoechst dye (blue) was used to visualize the cell nuclei. Overlay of bright field images with blue and green fluorescence (E) indicates that much of the fluorescein-labeled peptides have been internalized. Quantification of SH-SY5Y binding (F) performed by measurement of fluorescein-positive cells through flow cytometry after gating for live cells based on scattering parameters (additional data in Fig. S31–S33 & Table S6). In each imaging experiment, the fluorescent peptides were incubated at a 250 nM concentration with the cells for 30 minutes; for competition experiment with NPY (B), 5 µM NPY was pre-incubated with SH-SY5Y cells for 30 minutes prior to the addition of the fluorescent probe. Scale bar = 50 µm. The images were obtained in 1-2 biological replicates, and representative images are shown. Bright field images, split channel images, and merged images of all conditions as well as controls (buffer or DMSO) are included in Fig. S22–S25.

We performed confocal microscopy experiments to determine whether our all-amide and thioamide imaging peptides could selectively bind Y_2_R-expressing SH-SY5Y cells, a commonly used neuroblastoma model cell line. As controls, we used two other cell lines with little to no Y_2_R expression; breast cancer cell line MCF-7, which expresses Y_1_R, and human embryonic kidney cell line HEK293T, which expresses neither Y_1_R nor Y_2_R. In addition, we performed competition experiments with known Y_2_R ligand NPY (peptide 7). Using the HAP-1 and HAP1-R^S^_33_ peptides labelled with 5(6)-carboxyfluorescein, we observed bright green, punctate fluorescence on SH-SY5Y cells, consistent with successful peptide-receptor binding and internalization (Fig. 4A). Pre-incubation with unlabeled NPY peptide effectively blocked the green fluorescence, indicating competitive binding and confirming the selectivity of our peptides for NPY receptors (Fig. 4B). No green fluorescence was observed when the labeled peptides were incubated with MCF-7 cells (Fig. 4C) or HEK293T cells (Fig. 4D). Quantification of binding by integrating fluorescein emission shows that HAP-1 and HAP1-R^S^_33_ have respective 17-fold and 14-fold increases over DMSO controls for SH-SY5Y cells, but no significant differences *vs.* controls for MCF-7 cells or HEK293T cells (Fig. S26).

Comparison of SH-SY5Y bright field images with fluorescence from a nuclear Hoechst dye (blue) and the fluorescence signal from the peptides (green) shows that the green fluorescence is punctate and within the cell body rather than a “halo” on the membrane, implying that much of the fluorescein-labeled peptides have been internalized (Fig. 4E). We conducted studies at either 4 °C or 37 °C using a membrane stain and found that cell labeling was significantly reduced at 4 °C, further supporting the importance of internalization since receptor-mediated endocytosis is prevented at 4 °C (Fig. S34–S39).

We also investigated internalization and binding affinity in flow cytometry experiments, in which SH-SY5Y cells were stained with different concentrations of HAP-1 or HAP1-R^S^_33_ peptides. At 37 °C, the percent of fluorescent live single cells at each peptide concentration was plotted to obtain dissociation constant (*K*_d_) values (Fig. 4F, Fig. S31–S33, Table S6). The *K*_d_ values are lower for both HAP1 and HAP1-R^S^_33_ were 2021 ± 418 nM and 1280 ± 182 nM, respectively. Interestingly, the HAP1-R^S^_33_ peptide had a similar, but slightly lower, *K*_d_ value than the HAP-1 peptide, in contrast to their relative EC_50_ values (Table 2). Fluorescent staining is lower at 4 °C than at 37 °C and requires higher concentrations to achieve saturation (Fig. S33). Taken together, our imaging and flow cytometry data show that HAP1 peptides bind specifically to Y_2_R-expressing cells *vs.* Y_1_R-expressing cells, with accumulation in the cells through internalization, making them suitable for imaging applications.

## Conclusions

Building upon the known agonist cyclic Ac-[Lys_28_-Glu_32_] NPY_25–36_ (Ac-RHYK̲K̲N̲L̲I̲RQRY-NH_2_)^18^ and mutations reported to improve Y_2_R activity from the literature (Oxo Cyclic_24–36_, peptide 3), we successfully designed and synthesized fluorescently labeled cyclic thioamide peptides targeting Y_2_R. We showed that we could strategically install a thioamide at the Arg_33_ position to improve the mouse serum stability of these peptides by 14-fold while retaining good agonist potency toward Y_2_R. More significantly, our cyclic labeled constructs HAP1 and HAP1-R^S^_33_ had selectivity toward Y_2_R, showing little to no potency when tested against Y_1_R-expressing cells.

We note that prior work has shown that while achieving Y_2_R:Y_1_R selectivity is extremely challenging, selectivity against the other NPY receptor subtypes is relatively straightforward due to their lower sequence homology and different pharmacological profiles.^1,3,9,39^ Thus, our testing focused on Y_2_R:Y_1_R selectivity. In future studies, it will be valuable to test selectivity against other NPY receptor subtypes, particularly Y_4_R and Y_5_R.

Our imaging and flow cytometry experiments showed that the HAP1 and HAP1-R^S^_33_ peptides could be used to stain and visualize Y_2_R-expressing SH-SY5Y neuroblastoma model cells. Given these results, we reaffirmed that thioamides can be strategically installed in fluorescently labeled peptides to make potent imaging constructs for specific receptors of interest. We are also interested in using a similar approach to make fluorescently labeled peptides for other disease-relevant receptors.

As the next steps for this work, in addition to the incorporation of thioamide at Arg_33_ position, we will evaluate whether we can further improve proteolytic stability by substituting residues at the peptide's C-terminus, such as Tyr_36_ replacement with *p*-fluoro-Phe (F^F^_36_) – a modification previously shown to improve selectivity toward Y_2_R in a study by Pedersen *et al.*^42^ It will also be interesting to test alternatives to deletion of Arg_25_ that can stabilize the N-terminal cleavage liability without losing Y_2_R affinity. These studies can be accompanied by more in-depth structural characterization, including CD and NMR. We will also conduct serum stability assays with human serum instead of mouse serum, for added translational relevance. We can test our constructs in tissues and animal models, replacing the carboxyfluorescein with near-infrared dyes that are more suitable for *in vivo* applications such as fluorescent guided surgery. Overall, this is a versatile peptide stabilization method that, with the right conjugation of imaging modalities, can also be translated into applications in PET and magnetic resonance imaging (MRI) for cancer imaging.

## Author contributions

H.A.T.P. and E.J.P. wrote the manuscript, with contributions and approval from the other authors. H.A.T.P. designed and performed most of the experiments. Y.C. conducted the imaging experiments. D.Y.Z. designed some earlier Y_2_R-targeting peptides that informed the work here, and D.Y.Z., T.M.B., and *E.G.* tested them. Y.C. and K.E.F. did the CD and NMR experiments.

## Conflicts of interest

There are no conflicts to declare.

## Supplementary Material

CB-007-D6CB00042H-s001

## Data Availability

The data supporting this article have been included as part of the supplementary information (SI). Supplementary information: experimental methods for peptide synthesis and characterization, proteolytic stability analysis, receptor activation studies, flow cytometry, microscopy, and structural studies. HPLC and MALDI MS data, additional microscopy images, CD and NMR data. See DOI: https://doi.org/10.1039/d6cb00042h.
