# Supplementary material for: Stabilized thioamide peptide agonists of the neuropeptide Y type 2 receptor for targeted cancer imaging
Source: RSC Chem Biol. 2026 Jun 12;7(8):1597–604. doi: 10.1039/d6cb00042h (PMC13262678; doi:10.1039/d6cb00042h)
Supplement: Supplementary file 1 [file CB-007-D6CB00042H-s001.pdf]

Supplementary Information (SI) for RSC Chemical Biology.
This journal is © The Royal Society of Chemistry 2026

Supporting Information

Stabilized Thioamide Peptide Agonists of the Neuropeptide Y Type 2 Receptor for Targeted Cancer Imaging

Hoang Anh T. Phan,[a] Yanan Chang,[a] Taylor M. Barrett,[a] Kristen E. Fiore,[a] Daniel Y. Zhang,[a] Ethan J. Grove,[a] E. James Petersson [a,b,*]

[a] *Department of Chemistry, School of Arts and Sciences, University of Pennsylvania, 231 South 34th Street, Philadelphia, PA 19104, USA.*

[b] *Department of Biochemistry and Biophysics, Perelman School of Medicine, University of Pennsylvania, 421 Curie Boulevard, Philadelphia, PA 19104, USA.*

[*]Email: ejpetersson@sas.upenn.edu

Table of Contents

1. General Information S2
2. Synthesis of Thioamide Acid Precursors S3
3. Solid Phase Peptide Synthesis (SPPS) of NPY Y_2R Peptides S3
4. Peptide Cleavage and Purification S7
5. Mouse Serum Stability Assay S10
6. Kallikrein Protease Assays S18
7. Neuropeptide Y Y_2 Receptor Activation Assays (Y_2R Agonist Assays) S23
8. Neuropeptide Y Y_1 Receptor Activation Assays (Y_1R Agonist & Antagonist Assays) S24
9. Cellular Imaging Experiments S26
10. Basic Structural Characterization of NPY Y_2R Imaging Peptide S33
11. Staining of SH-SY5Y Cells with Imaging Fluorescent Peptides at 4 ºC and 37 ºC S36
12. Imaging Temperature-Dependent Internalization of Peptides by SH-SY5Y Cells S41
13. References S46

1. General Information

(7-Azabenzotriazol-1-yloxy)tripyrrolidino-phosphonium hexafluorophosphate (PyAOP) was purchased from Chem-Impex (Wood Dale, IL, USA). All other fluorenylmethoxycarbonyl (Fmoc) protected amino acids and 2-(1*H*-benzotriazol-1-yl)-1,1,3,3-tetramethyluronium hexafluorophosphate (HBTU) were purchased from EMD Novabiochem (currently MilliporeSigma, EMD Millipore; Billerica, MA, USA). Piperidine, N,N-diisopropylethylamine (DIPEA), and mouse serum (M5905) were purchased from Sigma-Aldrich (St, Louis, MO, USA). cAMP Hunter™ eXpress NPY2R CHO-K1 GPCR Assay and PathHunter® eXpress NPY1R CHO-K1 β-Arrestin GPCR Assay kits were purchased from DiscoverX (Fremont, CA, USA). SH-SY5Y, HEK293T, and MCF7 cells were purchased from ATCC (Manassas, VA, USA). 2,2,2-Trifluoroethanol-d3 was purchased from Cambridge Isotope Laboratories (Tewksbury, MA, USA). All other reagents were purchased from Fisher Scientific (Pittsburgh, PA, USA) unless specified otherwise. Milli-Q filtered (18 MΩ) water was used for all solutions (EMD Millipore). Peptides were purified with an Agilent 1260 Infinity II Preparative HPLC system and analyzed with an Agilent 1260 Infinity II Analytical HPLC system (Santa Clara, CA, USA). Peptide mass spectrometry was collected with a Bruker Ultraflex III matrix-assisted laser desorption ionization mass spectrometer (MALDI MS), Bruker MicrofleX (MALDI-TOF MS), or Bruker RapifleX (MALDI-TOF/TOF) (Billerica, MA, USA). Absorption spectra were collected on a GENESYS 150 UV-vis spectrophotometer (Thermo Fisher Scientific; Waltham, MA, USA). Circular dichroism (CD) data were acquired with a Jasco J-1500 CD spectrometer. Nuclear magnetic resonance (NMR) spectra were collected with a Bruker AVANCE NEO 600 MHz spectrometer. (Billerica, MA). Cellular images were obtained with an Olympus CKX53 microscope, and confocal images were obtained using an Olympus FV1000 laser scanning confocal microscope.

2. Synthesis of Thioamide Acid Precursors

N_{α}-Fmoc-N_{ω}-Pbf-L-thioarginine-nitrobenzotriazolide were synthesized and characterized as previously published procedures by our laboratory.[1]

3. Solid Phase Peptide Synthesis (SPPS) of NPY Y_2R Peptides

Each peptide was manually synthesized on a 100 µmol scale on Rink Amide resin based on our established protocols.[12] For a typical synthesis, Rink Amide resin was added to a dry reaction vessel (RV) and initially swelled in 4 mL dimethylformamide (DMF) for 2 x 15 minutes with magnetic stirring. The resin was deprotected with 4 mL of 20% piperidine solution in DMF for 20 minutes. Between each reaction, the resin was washed extensively with sufficient amount of DMF, DCM (dichloromethane), and DMF, but always ended with the condition of the next reaction. For coupling of a regular amino acid, 5 equiv. of amino acid and acid and 5 equiv. of PyAOP (7-Azabenzotriazol-1-yloxy)tripyrrolidino-phosphonium hexafluorophosphate) was dissolved in 4 mL of DMF and added to the RV, following an addition of 10 equiv. of DIPEA. The reaction ran for 45 minutes under stirring; a second coupling for the first amino acid was done to ensure coupling efficiency. After coupling on the first amino acid and washing, the resin was stirred in 4 mL of 20% piperidine solution in DMF for 20 minutes for deprotection reaction. The same coupling and deprotection protocol were applied for all of the subsequent regular amino acids. For the thioamide analog, thioamide residues were coupled and deprotected with slightly modified procedures. Thioamides were coupled through pre-activated precursors, where 2.5 equiv. of the thioamide precursor was dissolved in 4 mL of dry dichloromethane (DCM) with 5 equiv. DIPEA and stirred for 2 x 45 minutes. This procedure was repeated prior to deprotection to ensure efficient incorporation of the thioaminoacid onto the peptide chains. After coupling of ThioArg and Fmoc-

Lys(Mtt)-OH amino acid, the resin was treated with 4 mL acetyl capping solution of DMF, methylmorpholine (NMM), and acetic anhydride (84:6:10 v/v). This reaction was repeated twice to ensure the acetyl capping has complete. For the deprotection of thioamides, 4 mL of 2% DBU (1,8-diazabicyclo(5.4.0)undec-7-ene) in DMF was added to the RV and reacted three times for 2 minutes each, with extensive washing with DMF and DCM between each deprotection step. For coupling of special amino acids such as Fmoc-Glu(O-2PhiPr)-OH, Fmoc-Lys(Mtt)-OH, and Fmoc-Ahx-OH, 2.5 equiv. of amino acid and acid and 2.5 equiv. of PyAOP were dissolved in 4 mL of DMF, then added to the RV with an addition of 5 equiv. of DIPEA and stirred for 2 x 45 minutes.

For the synthesis of cyclic peptides, to cyclize the peptide, Glu(O-2PhiPr) and Lys(Mtt) were first selectively deprotected in 4 mL of trifluoroacetic acid (TFA), triisopropylsilane (TIPS), Milli-Q water, and DCM (5:5:5:85 v/v) for 10 x 10 minutes. This was followed by a bridge coupling reaction with 2 equiv. of PyAOP and 4 equiv. of DIPEA in 4 mL of DMF. This cyclization reaction was run for 2 x 1 hour and 45 minutes.

The fluorescent dye was coupled onto the peptide via a hexanoic acid linker. Following the coupling of the N-terminal Fmoc-Lys(Mtt)-OH and the final deprotection reaction, the peptide was first acetylated with a 4 mL capping solution containing DMF, *N*-methylmorpholine, and acetic anhydride (84:6:10 v/v) for 2 x 10 minutes, then again selectively deprotected with 4 mL of TFA, TIPS, Milli-Q water, and DCM (5:5:5:85 v/v) for 10 x 10 minutes before coupling the linker Fmoc-Ahx-OH. For the final coupling of 5(6)-carboxyfluorescein (FAM), 5 eq. of the amino acid was dissolved in 4 mL DMF with 4.5 equiv. of HBTU and 10 equiv. of DIPEA and the reaction was run overnight at room temperature. The general synthetic schemes for the linear and cyclic peptides were outlined in **Fig. S1** and **Fig. S2**.

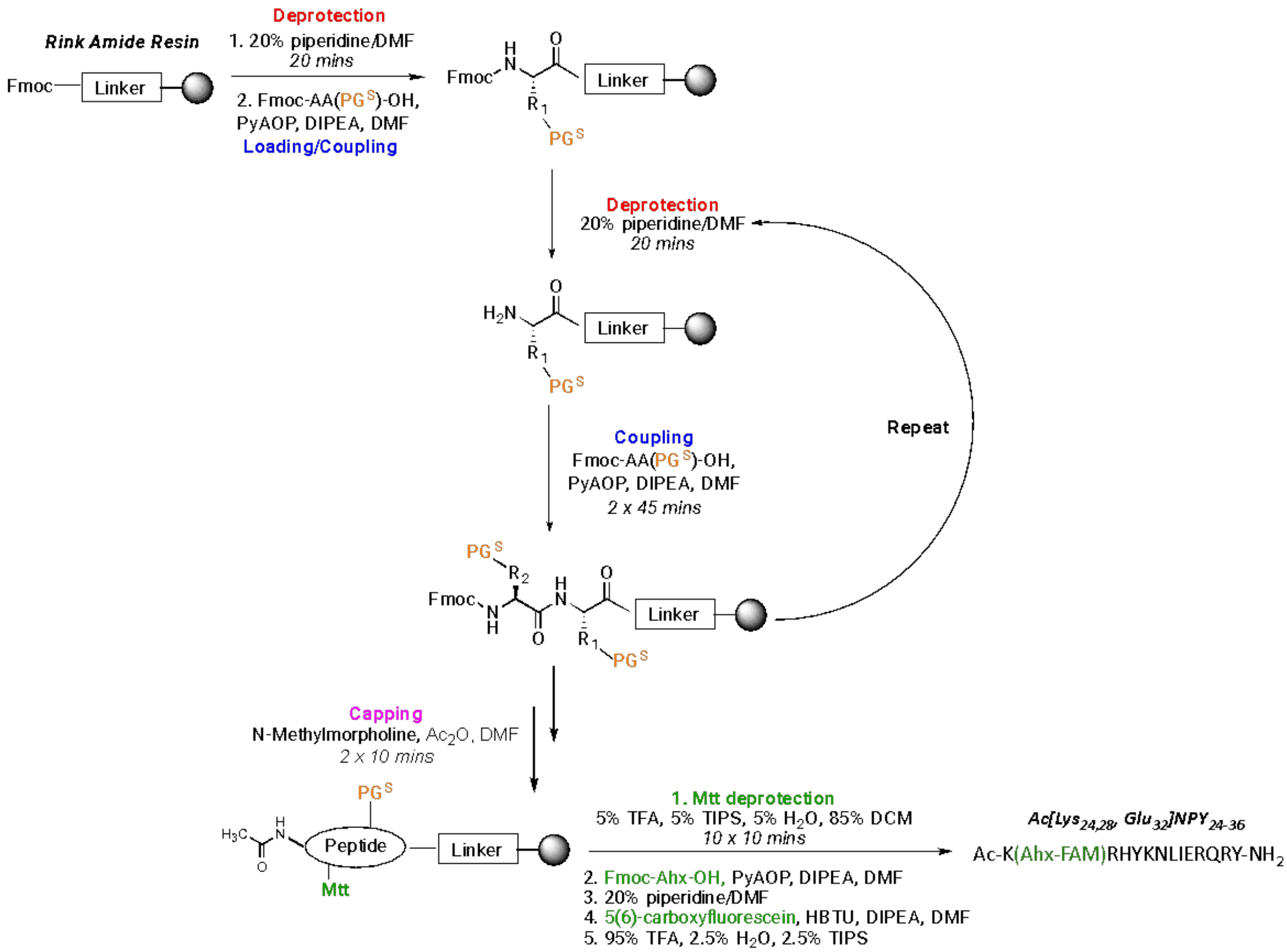

Fig. S1. Synthesis of Oxo Linear$_{24\text{-}36}$ (**1**).

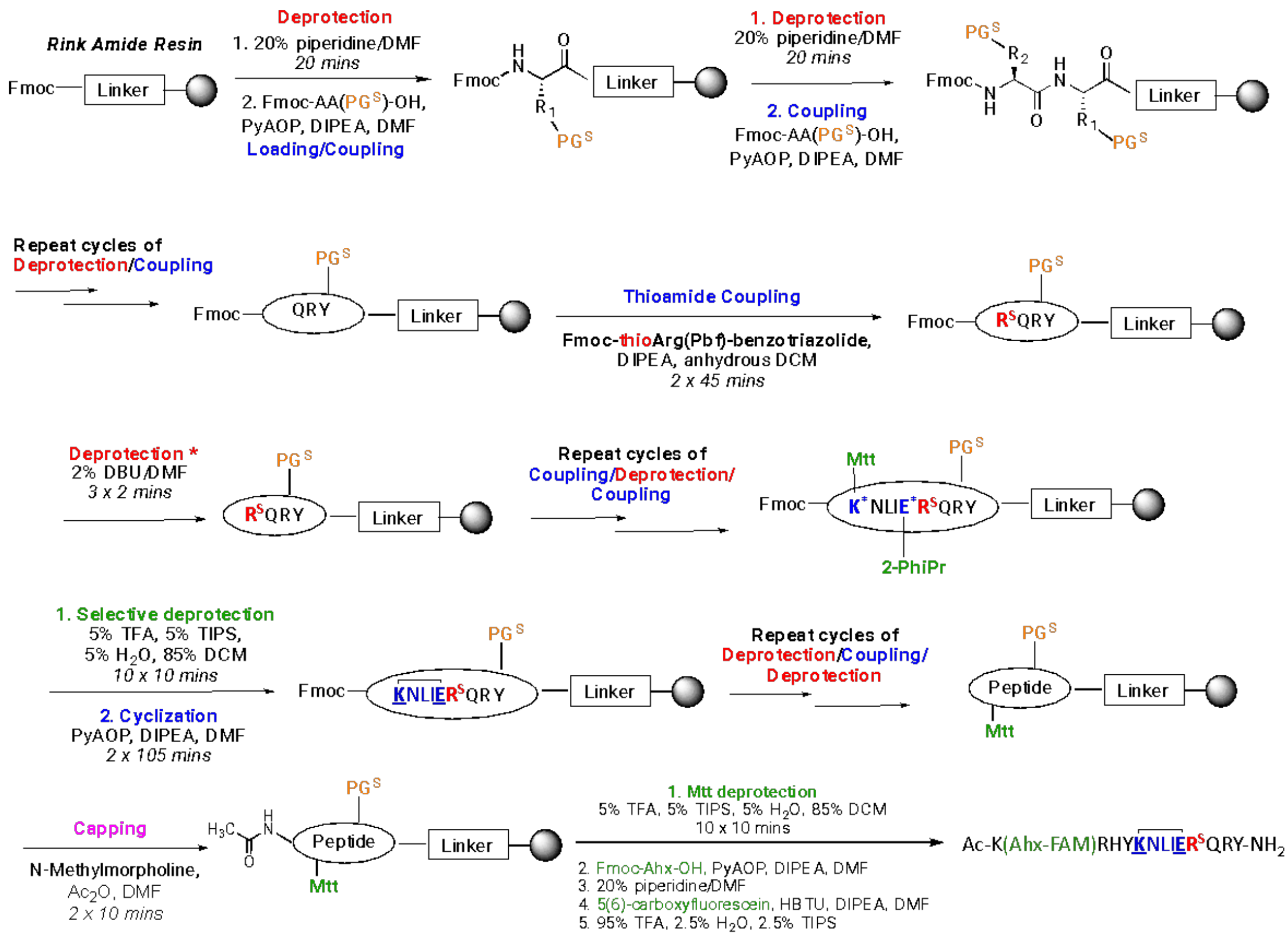

Fig. S2. Synthesis of Oxo Cyclic$_{24\text{-}36}$ (**3**) and HAP1 (**5**).

4. Peptide Cleavage and Purification

Upon completion of the synthesis, the resin was dried with DCM under vacuum. Peptides were cleaved from resin by treatment with a 6 mL fresh cleavage cocktail of TFA, Milli-Q water, and TIPS (95:2.5:2.5 v/v) for 45 minutes with stirring. After treatment, the cocktail solution was expelled from the RV with nitrogen, washed with DCM, and reduced to a volume of less than 1 mL by rotary evaporation. This resulting solution was then treated with about 30 mL of cold ethyl ether in order to precipitate out the peptides and transferred into a Falcon tube. The peptide was centrifuged down in a Tabletop Centrifuge at 4,000 rpm for 10 minutes and the supernatant was discarded into another tube. This resulting precipitate was flash frozen with liquid nitrogen and evaporated using lyophilization. The crude peptide was diluted in CH_3CN/H_2O (10:90 v/v) and then purified on a Luna® Omega 5 µm PS C18 100 Å, LC preparative column (Phenomenex; Torrance, CA, USA) by an Agilent 1260 Infinity II Preparative HPLC, using a 25-35% acetonitrile (CH_3CN) gradient, as detailed in the **Table S1** and **Table S2**, at a flow rate of 10 mL/min. MALDI MS was used to confirm peptide identities (**Table S3**). Purified peptides were dried on a lyophilizer (Labconco; Kansas City, MO, USA) or in a vacuum centrifuge (Savant/Thermo Scientific; Rockford, IL, USA). All peptides were subjected to multiple rounds of purification until 99% purity by analytical HPLC was achieved. It was noted that only the major isomer of the (5)6-FAM conjugated peptides were further purified and used for subsequent experiments.

Table S1. Peptide Purification Methods and Retention Time.

No.	Peptide (Abbreviation)	Sequence	Gradient	Retention Time
**1**	Ac[K$_{24,28}$E$_{32}$]NPY$_{24-36}$ (Oxo Linear$_{24-36}$)	Ac-K*RHYKNLIERQRY-NH$_2$	1	12.4
**2**	Ac[K$_{24,28}$E$_{32}$R$^S_{33}$]NPY$_{24-36}$ (Thio Linear$_{24-36}$)	Ac-K*RHYKNLIE**R**^{S}QRY-NH$_2$	1	13.5
**3**	Ac-Cyclo$_{28-32}$[K$_{24,28}$E$_{32}$]NPY$_{24-36}$ (Oxo Cyclic$_{24-36}$)	Ac-K*RHYKNLIERQRY-NH$_2$	1	17.8
**4**	Ac-Cyclo$_{28-32}$ [K$_{24,28}$E$_{32}$R$^S_{33}$]NPY$_{24-36}$ (Thio Cyclic$_{24-36}$)	Ac-K*RHYKNLIE**R**^{S}QRY-NH$_2$	1	19.2
**5**	Ac-Cyclo$_{28-32}$[K$_{25,28}$E$_{32}$R$^S_{33}$]NPY$_{25-36}$ (HAP1)	Ac-K*HYKNLIERQRY-NH$_2$	1	20.2
**6**	Ac-Cyclo$_{28-32}$ [K$_{25,28}$E$_{32}$R$^S_{33}$]NPY$_{25-36}$ (HAP1-R$^S_{33}$)	Ac-K*HYKNLIE**R**^{S}QRY-NH$_2$	1	21.0

* Abbreviation: R^S: thioarginine

Table S2. HPLC Gradient for Peptide Purification.

No.	Time (min)	% B
1	0:00	20
	3:00	20
	5:00	25
	25:00	35
	26:00	50
	27:00	100
	29:00	100
	33:00	50
	35:00	20

* Solvent A: 0.1 % TFA in water; Solvent B: 0.1 % TFA in acetonitrile

Table S3. Calculated and Observed Masses of Peptides.

Peptide	$[M+H]^+$		$[M+Na]^+$	
	Calculated	Observed	Calculated	Observed
Oxo Linear$_{24\text{-}36}$ (**1**)	2316.17	2315.176	2338.15	2338.064
Thio Linear$_{24\text{-}36}$ (**2**)	2332.14	2332.575	2354.12	2354.562
Oxo Cyclic$_{24\text{-}36}$ (**3**)	2298.16	2298.105	2320.14	-
Thio Cyclic$_{24\text{-}36}$ (**4**)	2313.13	2313.029	2335.11	-
HAP1 (**5**)	2142.06	2142.208	2164.04	2164.375
HAP1-R^S_{33} (**6**)	2158.03	2158.108	2179.91	-

* Abbreviation: R^S: thioarginine

5. Mouse Serum Stability Assay

A 25 µL solution of 150 µM peptide in sterile Mill-Q water was incubated at 37 °C in the presence of 25 µL mouse serum (Sigma Aldrich M5905). After incubating for the desired time (t = 0, 30, 60, 80, 100, and 120 minutes), the serum proteins were precipitated out with 50 µL methanol then incubated at – 20 °C for 10 minutes. For the cyclic thioamide peptide, assays were done with longer incubation time (t = 0, 1, 2, 4, 5, and 8 hours). The samples were pelleted by centrifugation at 13,000 rpm for 15 minutes at 4 °C using an Eppendorf 5415R centrifuge. To prepare samples for analysis with analytical HPLC, 80 µL of supernatant was diluted to 200 µL with Milli-Q water after adding 0.5 µL of 300 µM BODIPY as internal standard. Samples were run in triplicate for each time point. All samples were analyzed by an Agilent 1260 Infinity II series Analytical HPLC using a Phenomenex Luna C8(2) Analytical column (Torrance, CA, USA) using the gradient in **Table S4**. All peptides were monitored at 484 nm and the amount of intact peptide was quantified by integrating peak areas. To determine the percent intact peptide in each sample, the internal standard was used for normalization of the amount of intact peptide; the average ratio of intact peptide from the three trials to internal standard was then compared to the ratio at t = 0 minutes. MALDI-MS was used to confirm the identity of the intact peptide and its cleavage products from the fractions collected from each HPLC run (**Figs. S3-S8**).

Table S4. Analytical HPLC Gradient for Serum Stability Assays.

No.	Time (min)	% B
1	0:00	2
	5:00	2
	15:00	25
	27:00	31
	41:00	45
	43:00	100
	45:00	100
	47:00	2

* Solvent A: 0.1 % TFA in water; Solvent B: 0.1 % TFA in acetonitrile

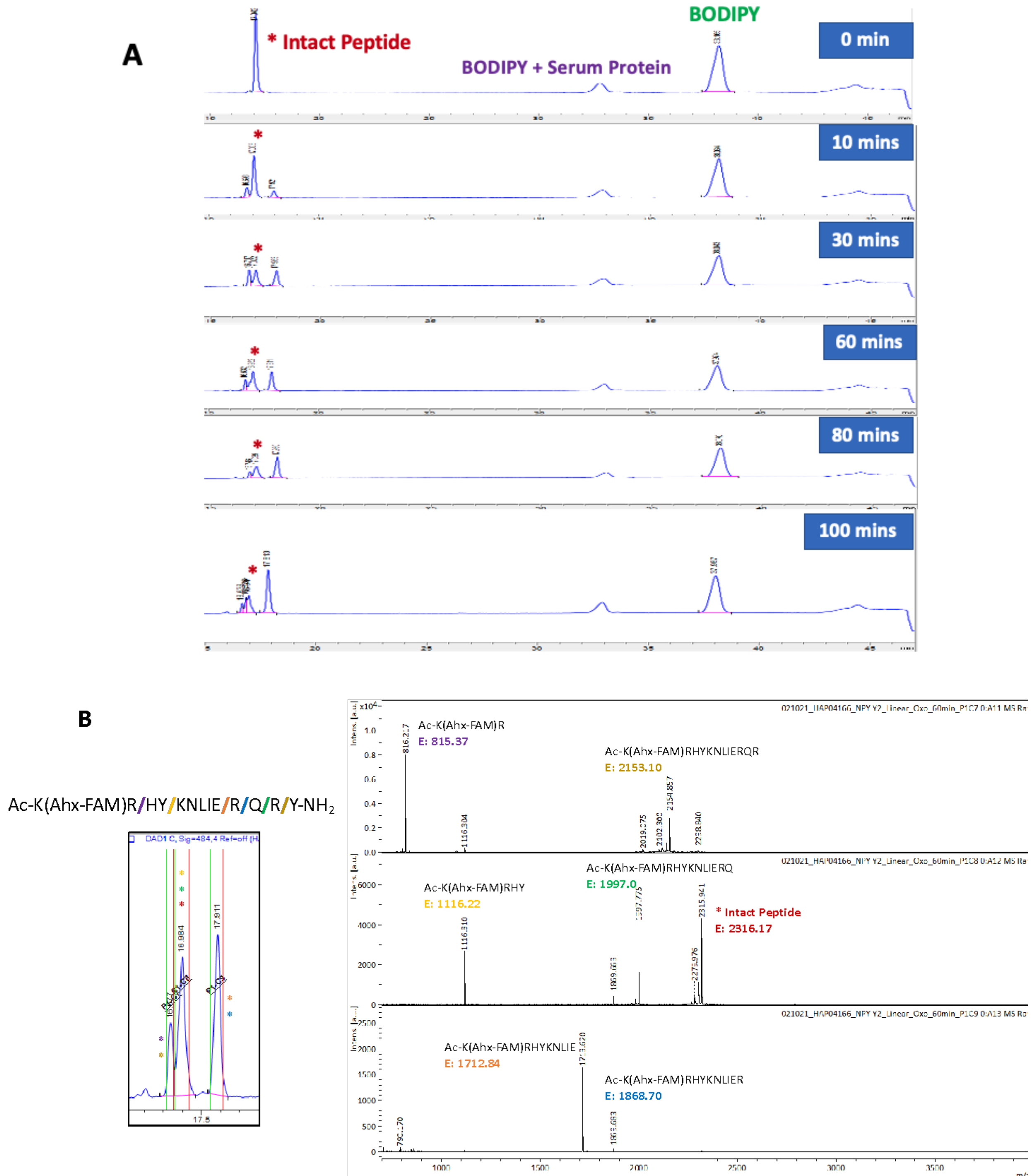

Fig. S3. HPLC and MALDI from Mouse Serum Stability Assays with Oxo Linear$_{24\text{-}36}$ Peptide (**1**). (A) HPLC collected at different time points of incubation with mouse serum. Intact peptides and the internal standard BODIPY are labeled. (B) MALDI MS collected from the HPLC fractions. Abbreviation: E is the expected mass; the slash / indicates cleavage site.

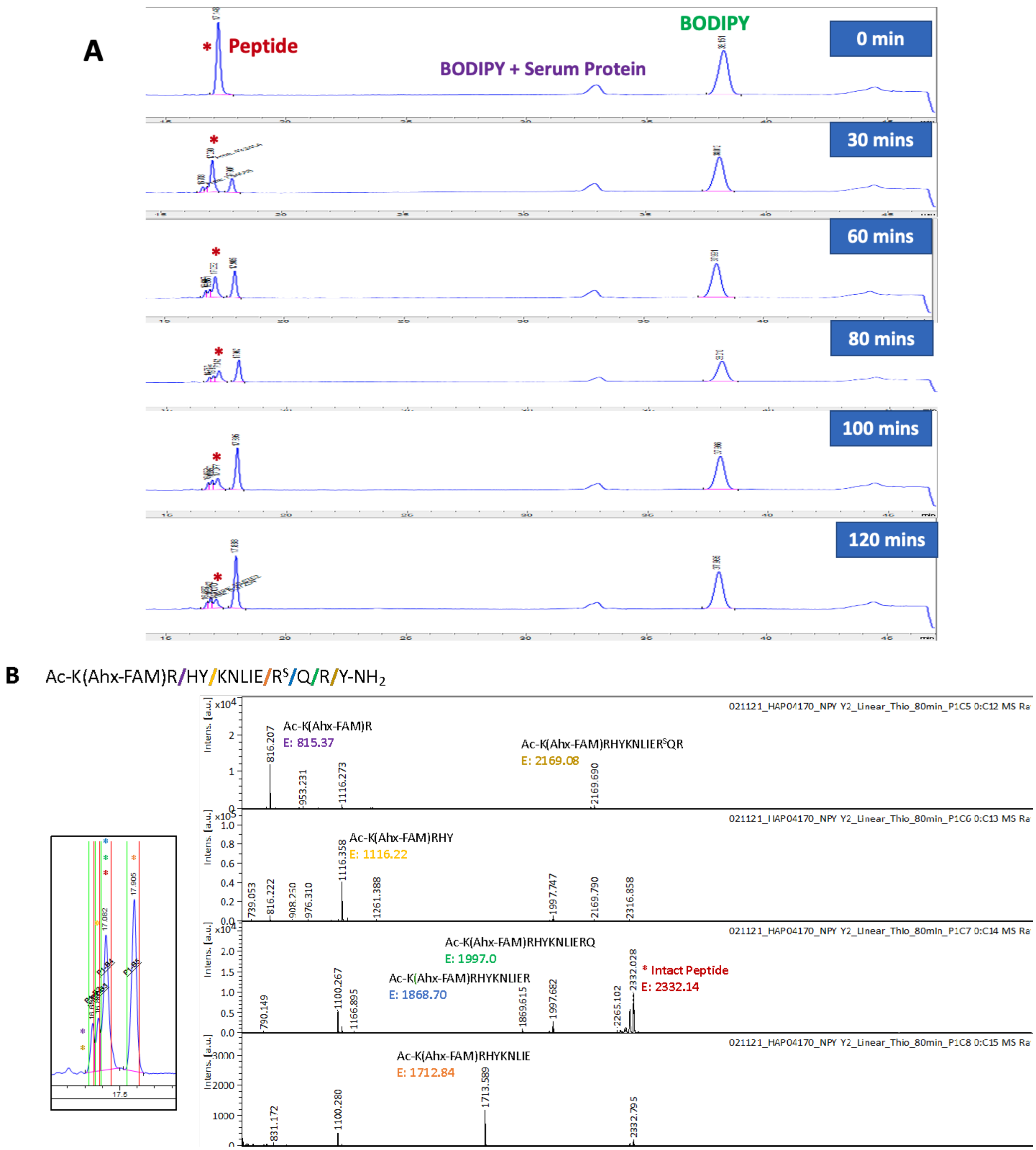

Fig. S4. HPLC and MALDI from Mouse Serum Stability Assays with Thio Linear$_{24\text{-}36}$ Peptide (**2**). (A) HPLC collected at different time points of incubation with mouse serum. Intact peptides and the internal standard BODIPY are labeled. (B) MALDI MS collected from the HPLC fractions. Abbreviation: E is the expected mass; the slash / indicates cleavage site.

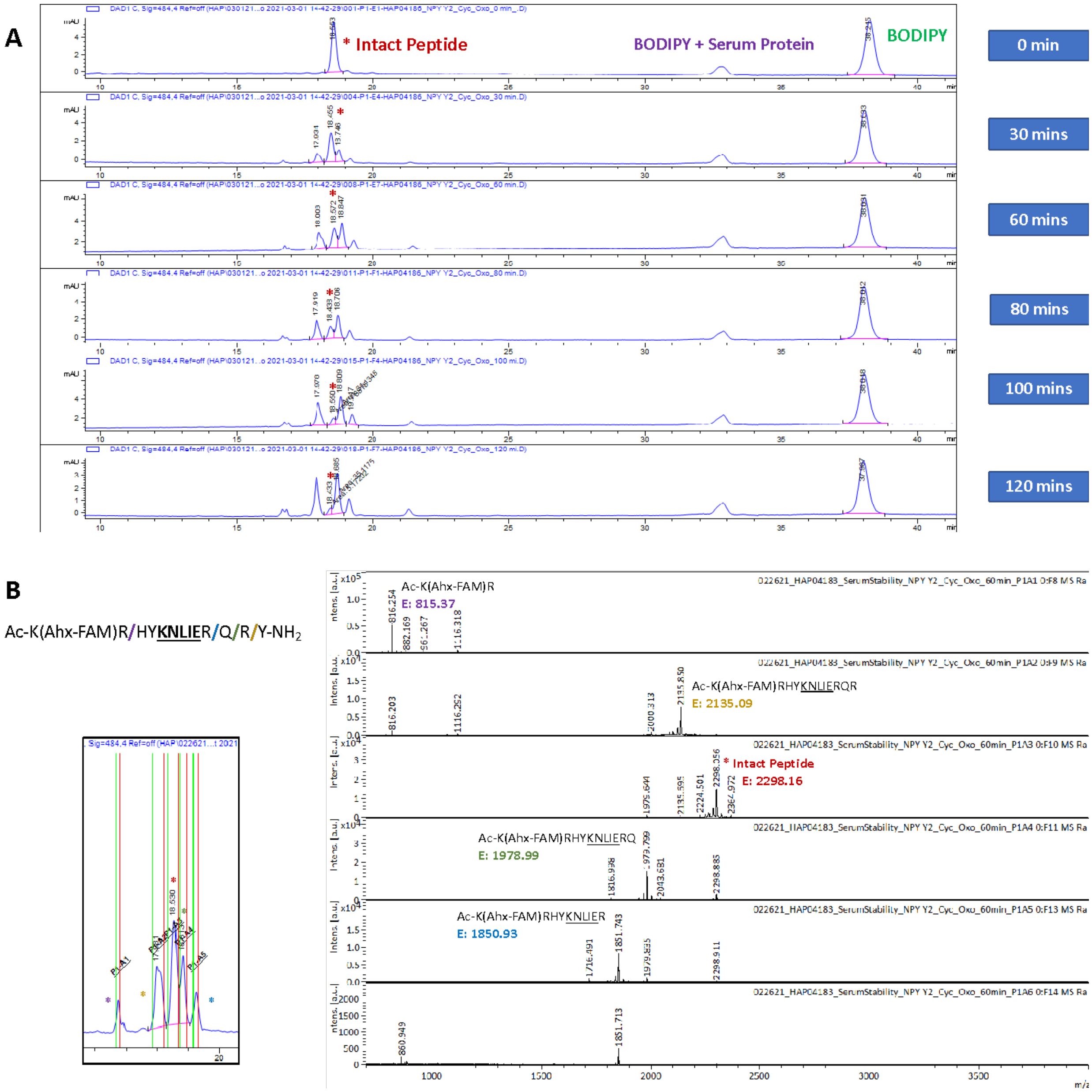

Fig. S5. HPLC and MALDI from Mouse Serum Stability Assays with Oxo Cyclic$_{24\text{-}36}$ Peptide (**3**). (A) HPLC collected at different time points of incubation with mouse serum. Intact peptides and the internal standard BODIPY are labeled. (B) MALDI MS collected from the HPLC fractions. Abbreviation: E is the expected mass; the slash / indicates cleavage site.

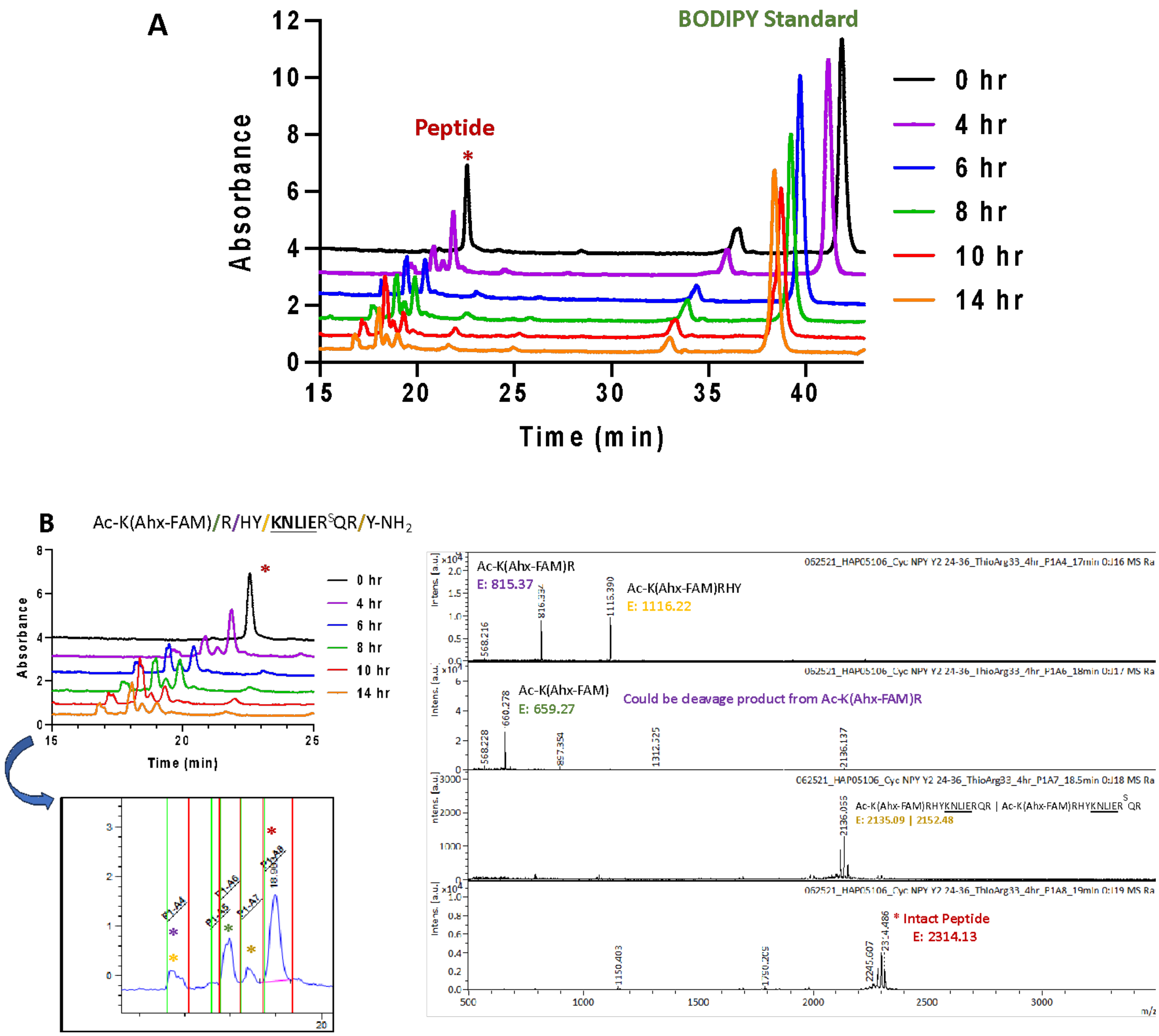

Fig. S6. HPLC and MALDI from Mouse Serum Stability Assays with Thio Cyclic$_{24\text{-}36}$ Peptide (**4**). (A) HPLC collected at different time points of incubation with mouse serum. Intact peptides and the internal standard BODIPY are labeled. (B) MALDI MS collected from the HPLC fractions. Abbreviation: E is the expected mass; the slash / indicates cleavage site.

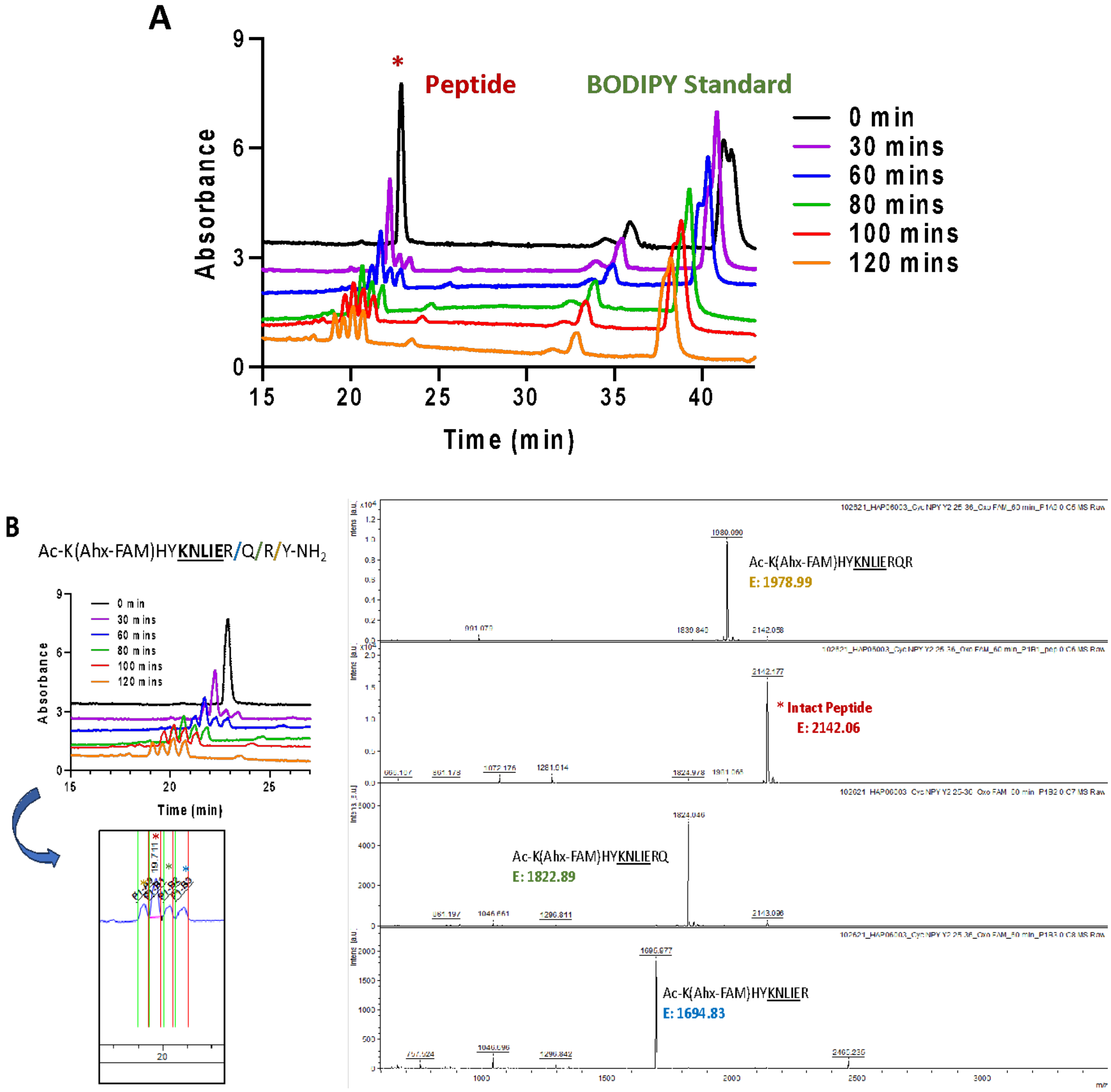

Fig. S7. HPLC and MALDI from Mouse Serum Stability Assays with HAP1 Peptide (**5**). (A) HPLC collected at different time points of incubation with mouse serum. Intact peptides and the internal standard BODIPY are labeled. (B) MALDI MS collected from the HPLC fractions. Abbreviation: E is the expected mass; the slash / indicates cleavage site.

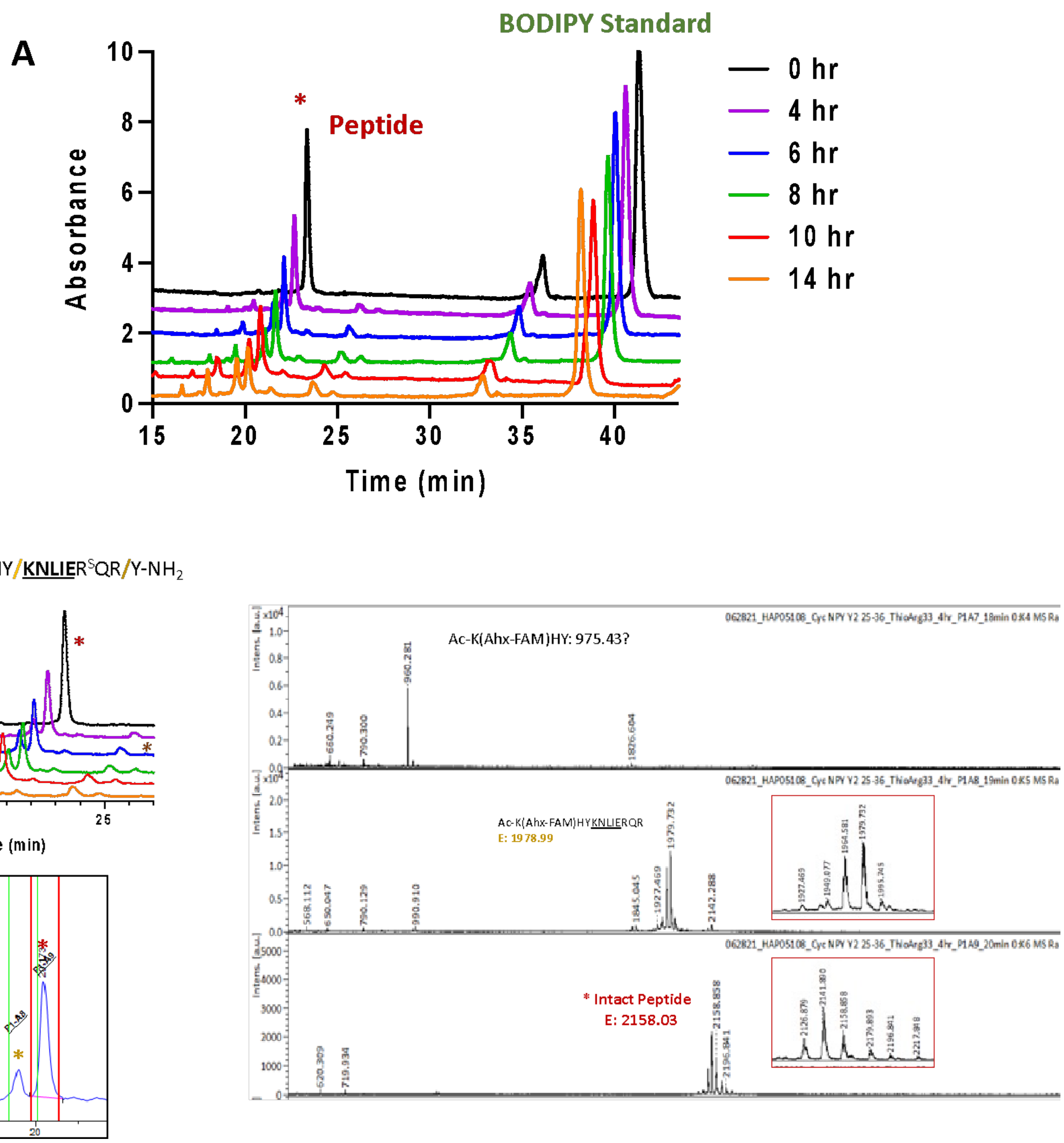

Fig. S8. HPLC and MALDI from Mouse Serum Stability Assays with HAP1-R^{S}_{33} Peptide (**6**). (A) HPLC collected at different time points of incubation with mouse serum. Intact peptides and the internal standard BODIPY are labeled. (B) MALDI MS collected from the HPLC fractions. Abbreviation: E is the expected mass; the slash / indicates cleavage site.

6. Kallikrein Protease Assays

A 40 µL solution of 39 µL of 40 µM peptide in buffer was incubated at 37 °C in the presence of 1 µL of 0.559 mg/mL kallikrein (Calbiochem 420307-50UG). After incubating for 30 minutes, the the proteolysis reactions were quenched with 20 µL 20% glacial acetic acid. To prepare samples for analysis with analytical HPLC, 60 µL of reaction mixture was diluted to a final volume of 200 µL with Milli-Q water, then added 0.5 µL of 300 µM BODIPY as internal standard prior to injection. Samples were run in triplicate for each time point. All samples were analyzed by an Agilent 1260 Infinity II series Analytical HPLC using a Phenomenex Luna C8(2) Analytical column (Torrance, CA, USA) using the gradient in **Table S5**. All peptides were monitored at 484 nm. MALDI-MS was used to confirm the identity of the intact peptide and its cleavage products from the fractions collected from each HPLC run (**Figs. S9-S15**).

Table S5. Analytical HPLC Gradient for Serum Stability Assays.

No.	Time (min)	% B
1	0:00	2
	5:00	2
	15:00	25
	27:00	31
	41:00	45
	43:00	100
	45:00	100
	47:00	2

* Solvent A: 0.1 % TFA in water; Solvent B: 0.1 % TFA in acetonitrile

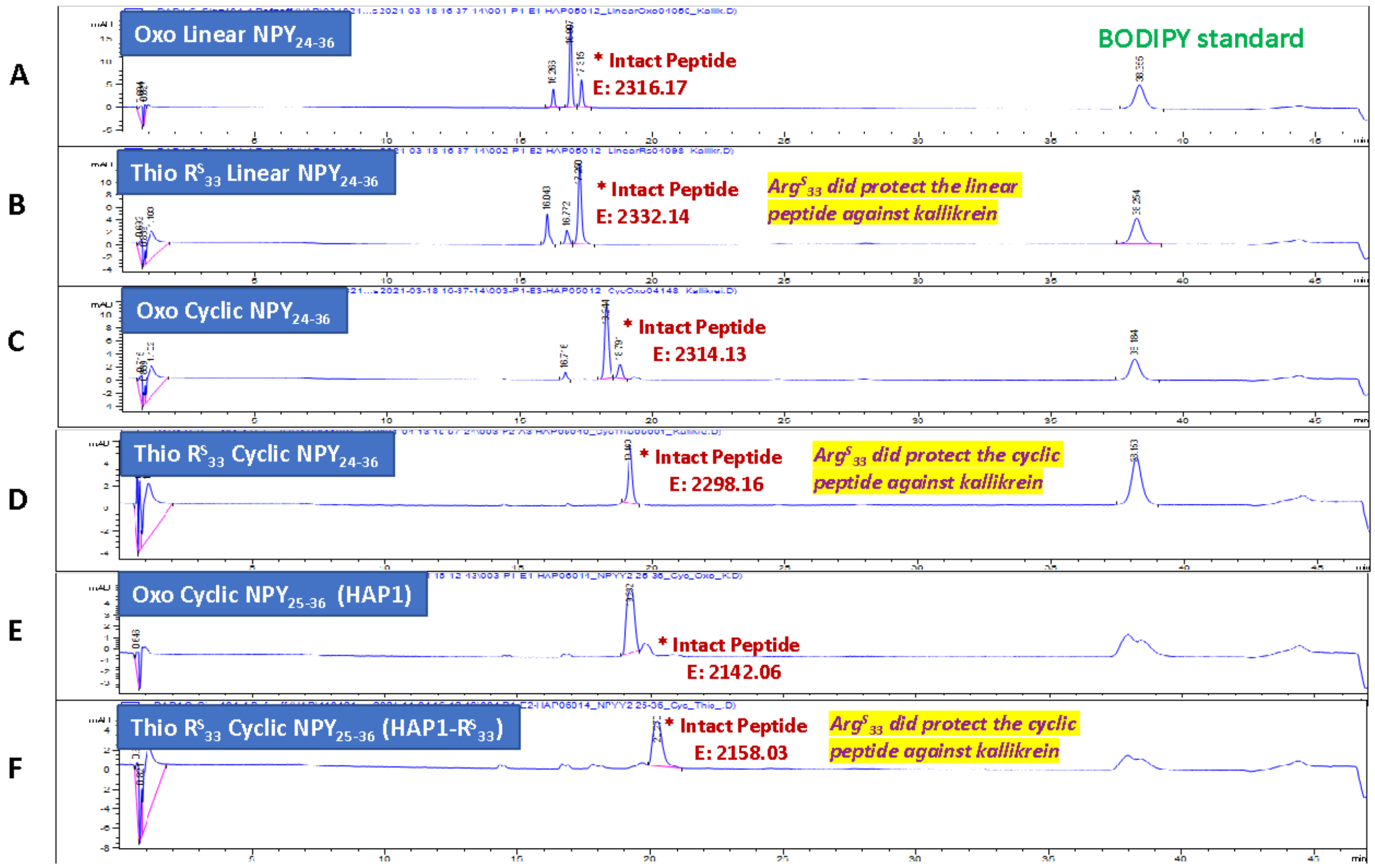

Fig. S9. Summary from Kallikrein Protease Assays with Linear$_{24\text{-}36}$, Cyclic $_{24\text{-}36}$, and HAP1 Peptides. HPLC collected at 30 minutes of incubation with kallikrein. Overall, there are less cleavage sites with kallikrein than the number of cleavage sites with the mouse serum assays. This suggested that there are other proteases present in the mouse serum that could cleave our peptides. Abbreviation: E is the expected mass.

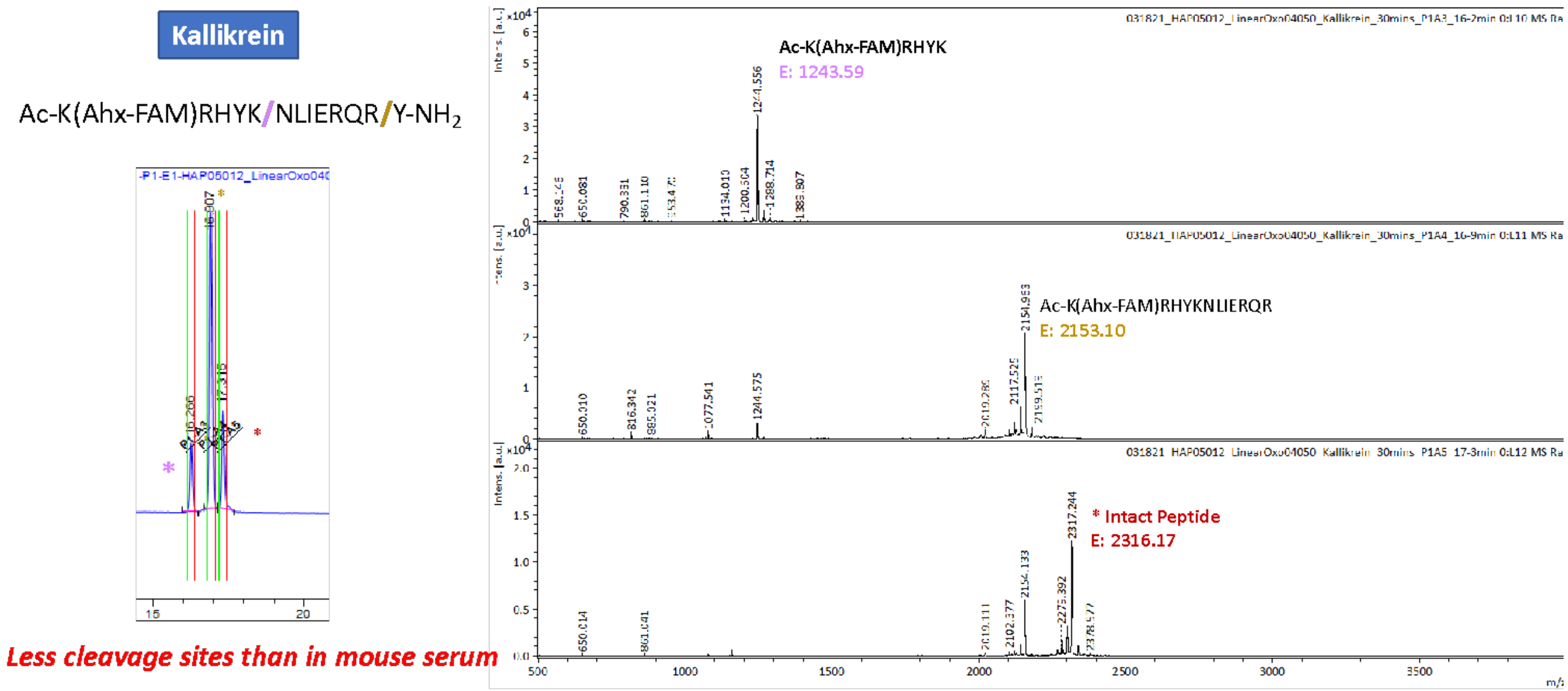

Fig. S10. HPLC and MALDI from Kallikrein Assays with Oxo Linear$_{24\text{-}36}$ Peptide (**1**). (A) HPLC collected at 30 minutes of incubation with kallikrein. Intact peptides and the internal standard BODIPY are labeled. (B) MALDI MS collected from the HPLC fractions. Abbreviation: E is the expected mass; the slash / indicates cleavage site.

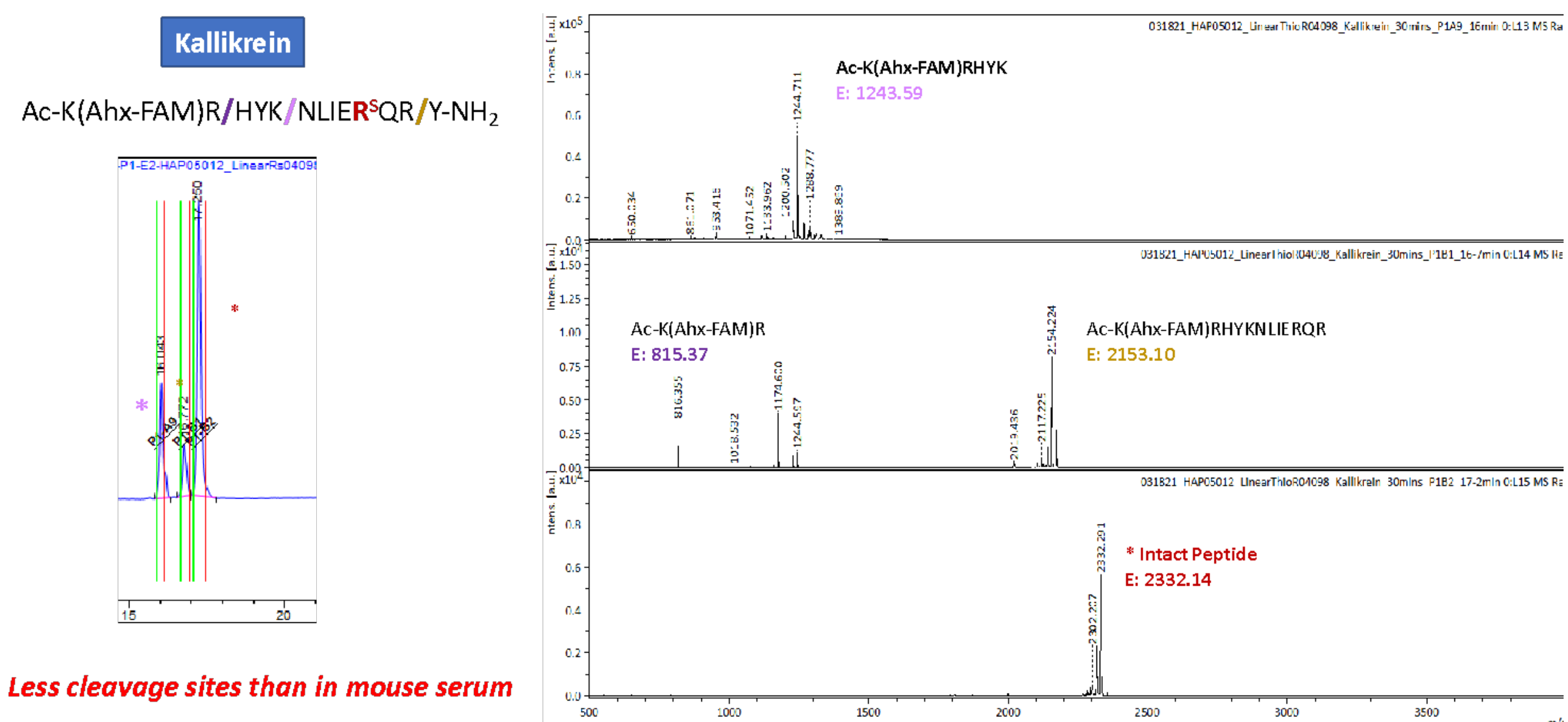

Fig. S11. HPLC and MALDI from Kallikrein Assays with Thio Linear$_{24\text{-}36}$ Peptide (**2**). (A) HPLC collected at 30 minutes of incubation with kallikrein. Intact peptides and the internal standard BODIPY are labeled. (B) MALDI MS collected from the HPLC fractions. Thioamidation at Arg protects the peptide from cleavage by kallikrein at the C-terminal of the peptide. Abbreviation: E is the expected mass; the slash / indicates cleavage site.

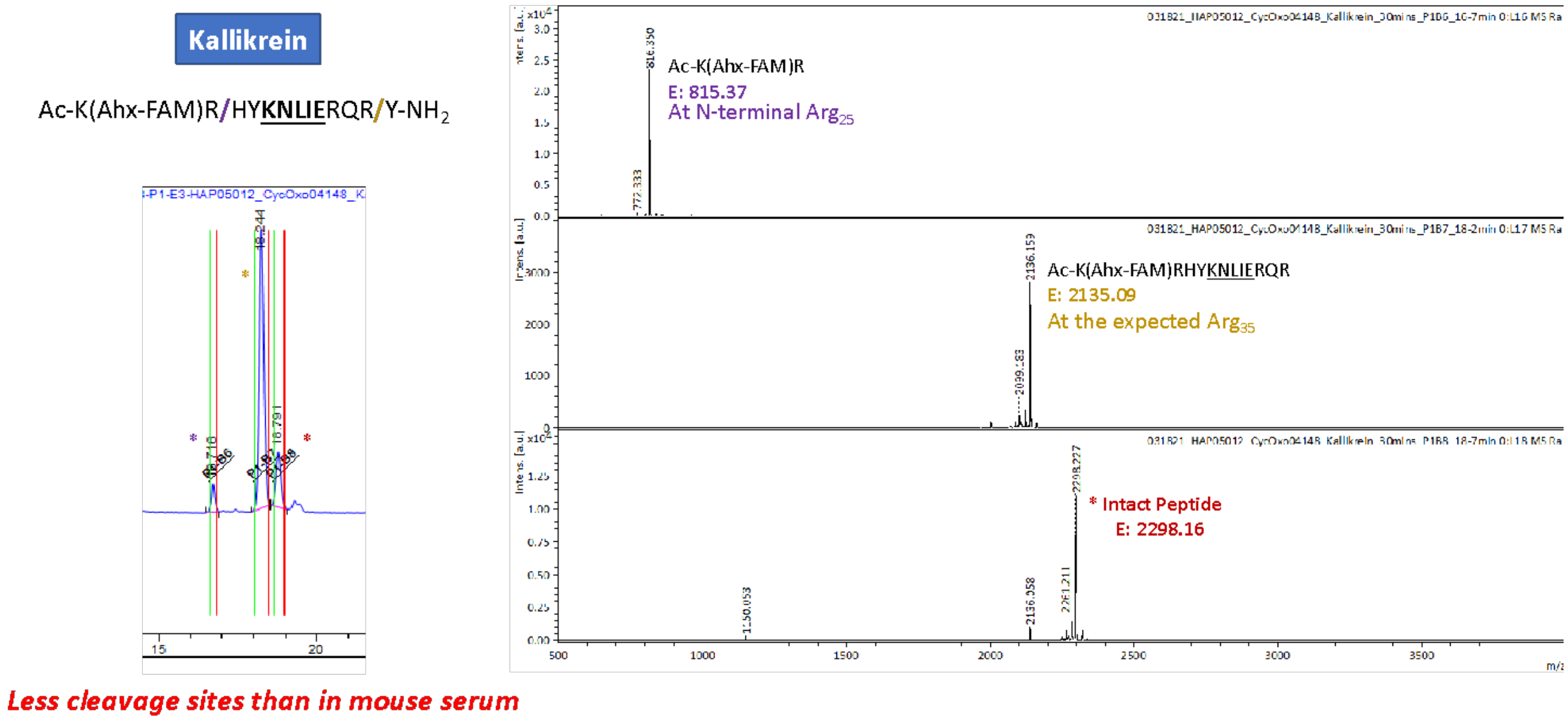

Fig. S12. HPLC and MALDI from Kallikrein Assays with Oxo Cyclic$_{24\text{-}36}$ Peptide (**3**). (A) HPLC collected at 30 minutes of incubation with kallikrein. Intact peptides and the internal standard BODIPY are labeled. (B) MALDI MS collected from the HPLC fractions. Abbreviation: E is the expected mass; the slash / indicates cleavage site; the underline indicates the cyclic portion of the peptide.

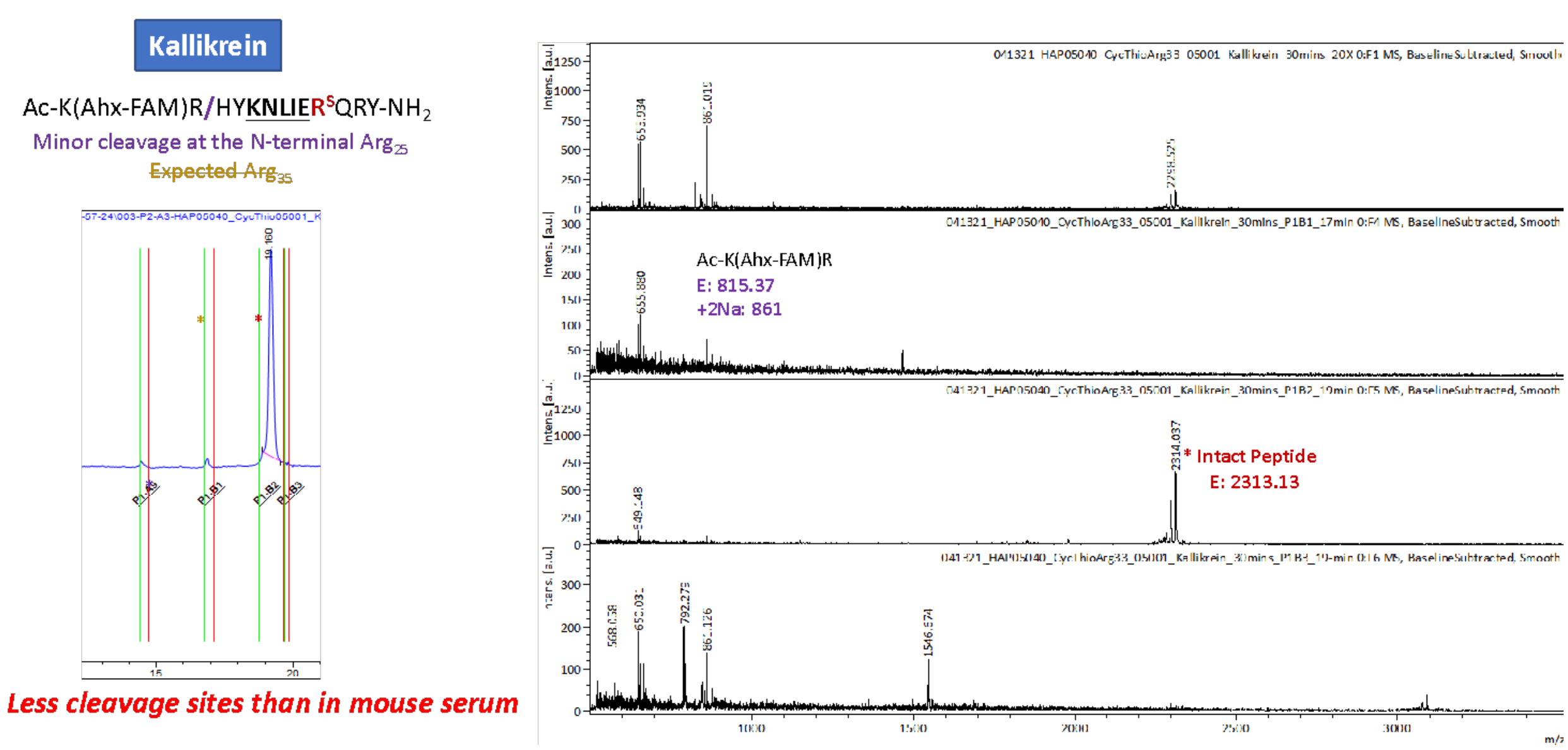

Fig. S13. HPLC and MALDI from Kallikrein Assays with Thio Cyclic$_{24\text{-}36}$ Peptide (**4**). (A) HPLC collected at 30 minutes of incubation with kallikrein. Intact peptides and the internal standard BODIPY are labeled. (B) MALDI MS collected from the HPLC fractions. Thioamidation at Arg protects the peptide from cleavage by kallikrein at the C-terminal of the peptide. Abbreviation: E is the expected mass; the slash / indicates cleavage site; the underline indicates the cyclic portion of the peptide.

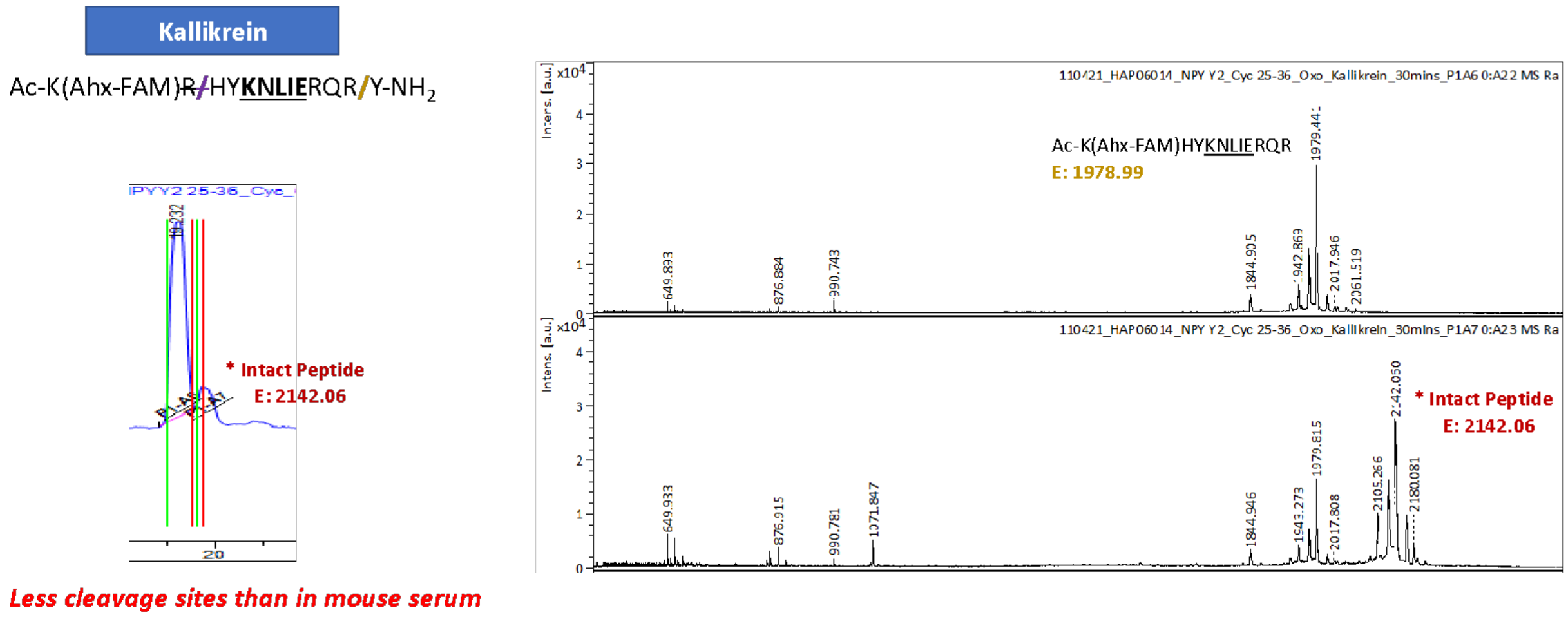

Fig. S14. HPLC and MALDI from Kallikrein Assays with HAP1 Peptide (**5**). (A) HPLC collected at 30 minutes of incubation with kallikrein. Intact peptides and the internal standard BODIPY are labeled. (B) MALDI MS collected from the HPLC fractions. By shortening the peptide by removing the Arg_{25}, we eliminated this cleavage site by the kallikrein (as shown in Fig. S12). Abbreviation: E is the expected mass; the slash / indicates cleavage site; the underline indicates the cyclic portion of the peptide.

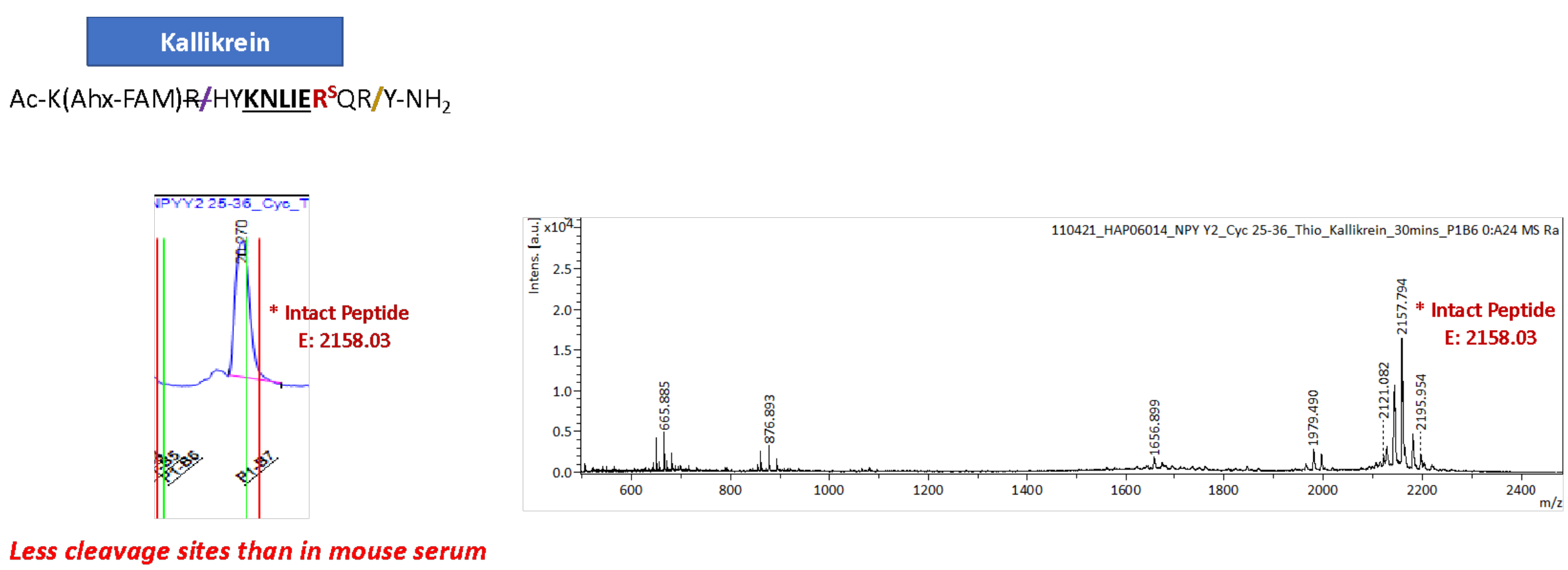

Fig. S15. HPLC and MALDI from Kallikrein Assays with HAP1-$R^S{}_{33}$ Peptide (**6**). (A) HPLC collected at 30 minutes of incubation with kallikrein. Intact peptides and the internal standard BODIPY are labeled. (B) MALDI MS collected from the HPLC fractions. By shortening the peptide by removing the Arg_{25}, we eliminated this cleavage site by the kallikrein (as shown in Fig. S13). Abbreviation: E is the expected mass; the slash / indicates cleavage site; the underline indicates the cyclic portion of the peptide.

7. Neuropeptide Y Y_2 Receptor Activation Assays (Y_2R Agonist Assays)

To determine the EC_{50} of HAP1 and HAP1-R^S_{33} peptides, a cAMP Hunter® eXpress NPY2R CHO-K1 GPCR Assay was employed, according to manufacturer's protocol. Provided cells were thawed and plated onto a 96-well plate with 100 µL cell plating reagent and stored in a 37 ºC incubator with 5% CO_2 overnight, for 18-24 hours. After incubation, cell plating reagent was removed and replaced with 30 µL cell assay buffer. Solutions of our peptides, peptide YY (PYY; control peptide) and Ac-[$Leu_{28,31}$]-NPY_{24-36} (control peptide) in cell assay buffer at varying concentrations were plated into a separate 96 well plate to be transferred to the assay plate. For the agonist assay, cells were treated with 15 µL 3X agonist (HAP1, HAP1- R^S_{33}, peptide YY (PYY), or Ac-[$Leu_{28,31}$]-NPY_{24-36}) for 30 minutes in the incubator (37 ºC with 5% CO_2). A cAMP curve (without any cells) could also be done to ensure that the assay works properly. Plates set up is in **Figs. S16-S17**. Then, cells were treated with 15 µL antibody solution and 60 µL cAMP working detection solution provided in the assay kit, followed by incubation at room temperature for 1 hour. The white-walled 96-well tissue culture treated plate was covered with tinfoil to avoid photobleaching. Finally, 60 µL cAMP solution A was added to the plate before it was read on a Tecan M1000pro in luminescence mode (no filter) with an integration time of 100 ms after incubation at 1 hour and 3 hours post-treatment. Within each trial, luminescence signal was normalized to the maximum and the minimum (treatment with buffer). The normalized data were then analyzed by averaging data across two-three independent biological replicates (different batches of cells) on different days.

	1	2	3	4	5	6	7	8	9	10	11	12
A	300nM PYY	50nM	10nM	5nM	1nM	0.5nM	0.1nM	0.05nM	0.005nM	0.001nM	30 uM Thio	0.12 uM
B	300nM PYY	50nM	10nM	5nM	1nM	0.5nM	0.1nM	0.05nM	0.005nM	0.001nM	10 uM	0.04 uM
C	300nM PYY	50nM	10nM	5nM	1nM	0.5nM	0.1nM	0.05nM	0.005nM	0.001nM	3.3 uM	0.013 uM
D	3000 nM Oxo	1000 uM	333 nM	111 nM	37 nM	12 nM	4 nM	1.3 nM	0.46 nM	0.15 nM	1.11 uM	0.004 uM
E	3000 nM Oxo	1000 uM	333 nM	111 nM	37 nM	12 nM	4 nM	1.3 nM	0.46 nM	0.15 nM	0.37 uM	0.0015 uM
F	3000 nM Oxo	1000 uM	333 nM	111 nM	37 nM	12 nM	4 nM	1.3 nM	0.46 nM	0.15 nM	Buffer + F	Buffer
G	30 uM Thio	10 uM	3.3 uM	1.11 uM	0.37 uM	0.12 uM	0.04 uM	0.013 uM	0.004 uM	0.0015 uM	Buffer + F	Buffer
H	30 uM Thio	10 uM	3.3 uM	1.11 uM	0.37 uM	0.12 uM	0.04 uM	0.013 uM	0.004 uM	0.0015 uM	Buffer + F	Buffer

Fig. S16. An example of the plate set up for Oxo and Thio Cyclic $_{24\text{-}36}$ peptides (**3** and **4**) for NPY Y_2R agonist assays.

	1	2	3	4	5	6	7	8	9	10	11	12
A	2.31 uM cAMP	770 nM	257 nM	85.6 nM	28.5 nM	9.51 nM	3.17 nM	1.06 nM	0.352 nM	0.117 pM	2.31 uM	9.51 nM
B	2.31 uM cAMP	770 nM	257 nM	85.6 nM	28.5 nM	9.51 nM	3.17 nM	1.06 nM	0.352 nM	0.117 pM	770 nM	3.17 nM
C	300nM PYY	50nM	10nM	5nM	1nM	0.5nM	0.1nM	0.05nM	0.005nM	0.001nM	257 nM	1.06 nM
D	300nM PYY	50nM	10nM	5nM	1nM	0.5nM	0.1nM	0.05nM	0.005nM	0.001nM	85.6 nM	0.352 nM
E	300nM PYY	50nM	10nM	5nM	1nM	0.5nM	0.1nM	0.05nM	0.005nM	0.001nM	28.5 nM	0.117 pM
F	100uM HAP1	60uM	30uM	10uM	3uM	1uM	0.3uM	0.1uM	0.01uM	0.001uM	Buffer + F	Buffer
G	100uM HAP1	60uM	30uM	10uM	3uM	1uM	0.3uM	0.1uM	0.01uM	0.001uM	Buffer + F	Buffer
H	100uM HAP1	60uM	30uM	10uM	3uM	1uM	0.3uM	0.1uM	0.01uM	0.001uM	Buffer + F	Buffer

Fig. S17. An example of the plate set up for HAP1 peptides (**5** and **6**) for NPY Y_2R agonist assays.

8. Neuropeptide Y Y_1 Receptor Activation Assays (Y_1R Agonist & Antagonist Assays)

A DiscoverX PathHunter® eXpress NPY1R CHO-K1 β-Arrestin GPCR Assay was employed. According to the manufacture's protocol, provided cells were thawed and plated onto a 96-well plate with 100 µL cell plating reagent and stored in a 37 ºC incubator with 5% CO_2 for 48 hours. Solutions of both peptides, HAP1 and HAP1- $R^S{}_{33}$, in DPBS buffer at varying concentrations were plated into a separate 96 well plate to be transferred to the assay plate.

If being tested for agonistic potency, cells were treated with 10 µL of 11X HAP1 or HAP1-$R^S{}_{33}$ in cell plating reagent for 30 minutes in the incubator (37 ºC with 5% CO_2). Plates set-up for agonist assays are shown in **Fig. S18**. If being tested for antagonist, cells were treated with 5 µL of HAP1 or HAP1-$R^S{}_{33}$ for 30 minutes in the incubator (37 ºC with 5% CO_2), followed by

treatment with 5 µL of 50 nM (final working concentration) peptide YY (PYY) for 1.5 hours in in the incubator (37 °C with 5% CO_2). Plates set-up for antagonist assays is shown in **Fig. S19**.

Next, cells were treated with the detection solution provided in the assay kit and allowed to incubate at room temperature for 1 hour. Finally, the assay plate luminescence was read on a Tecan M1000pro in luminescence mode (no filter) with an integration time of 100 ms after incubation at 1 hour and 3 hours post-treatment. Within each trial, luminescence signal was normalized to the maximum and the minimum (treatment with buffer). Each condition was done in technical triplicates.

	1	2	3	4	5	6	7	8	9	10	11	12
A	909 nM PYY	303	101	34	11	4	1	0.4	0.1	0.05	909	4
B	909 nM PYY	303	101	34	11	4	1	0.4	0.1	0.05	303	1
C	91 uM HAP1	30	10	3	1	0.4	0.1	0.04	0.01	0.005	101	0.4
D	91 uM HAP1	30	10	3	1	0.4	0.1	0.04	0.01	0.005	34	0.1
E	91 uM HAP1	30	10	3	1	0.4	0.1	0.04	0.01	0.005	11	0.05
F	91 uM HAP1-R^S_{33}	30	10	3	1	0.4	0.1	0.04	0.01	0.005	Buffer	Buffer
G	91 uM HAP1-R^S_{33}	30	10	3	1	0.4	0.1	0.04	0.01	0.005	Buffer	Buffer
H	91 uM HAP1-R^S_{33}	30	10	3	1	0.4	0.1	0.04	0.01	0.005	Buffer	Buffer

Fig. S18. An example of the plate set up for HAP1 peptides (**5** and **6**) for NPY Y_1R agonist assays.

	1	2	3	4	5	6	7	8	9	10	11	12
A	909 nM PYY	303	101	34	11	4	1	0.4	0.1	0.05	909	4
B	909 nM PYY	303	101	34	11	4	1	0.4	0.1	0.05	303	1
C	45 uM HAP1	15	5	2	1	0.2	0.1	0.02	0.01	0.002	101	0.4
D	45 uM HAP1	15	5	2	1	0.2	0.1	0.02	0.01	0.002	34	0.1
E	45 uM HAP1	15	5	2	1	0.2	0.1	0.02	0.01	0.002	11	0.05
F	45 uM HAP1-R^S_{33}	15	5	2	1	0.2	0.1	0.02	0.01	0.002	Buffer + 50 nM PYY	Buffer
G	45 uM HAP1-R^S_{33}	15	5	2	1	0.2	0.1	0.02	0.01	0.002	Buffer + 50 nM PYY	Buffer
H	45 uM HAP1-R^S_{33}	15	5	2	1	0.2	0.1	0.02	0.01	0.002	Buffer + 50 nM PYY	Buffer

Fig. S19. An example of the plate set up for HAP1 peptides (**5** and **6**) for NPY Y_1R antagonist assays.

9. Cellular Imaging Experiments

The following media were used in cell culture: DMEM or DMEM:F12 (Corning), DMEM:F12 no phenol (Thermo Fisher), 10% FBS (Atlanta Biologicals), 1% Glutamine Supplement (Gibco), 1% Penicillin/Streptomycin (Gibco). SH-SY5Y, HEK-293T, and MCF-7 cells were cultured in the appropriate media in 100 mm dishes prior to the experiment. SH-SY5Y cells were cultured in 89% DMEM:F12 (1:1), 10% fetal bovine serum, and 1% penicillin/streptomycin. HEK-293T and MCF-7 cells were cultured in 89% DMEM, 10% fetal bovine serum, and 1% penicillin/streptomycin. For the cell imaging experiment, 25 μg/mL poly-D-lysine solution was premade with sterile water (tissue culture grade; CCS 1180) and poly-D-lysine hydrobromide (Sigma P6407). The culture surface of ibidi μ- slide 8 well imaging chambers was aseptically coated with 300 μL of the poly-D-lysine solution (300 μL /well or per 25 cm^2) and incubated for 1 hour at room temperature. The solution was aspirated, and each well was rinsed with sterile tissue culture grade sterile water 3 times as excess poly-D-lysine could be toxic to cells. The coated vessel was uncovered in the laminal hood to dry for at least 2 hours before introducing cells and medium.

When cells reached 60-70% confluency in a 100 mm dish, the medium was aspirated. Cells were washed with DPBS without Ca^{2+} and Mg^{2+} then aspirated. Cells were then trypsinized with 3 mL trypsin-EDTA for 5 minutes and neutralized with 10 mL medium. The mixture of cells and solution were transferred into a 15 mL falcon tube and centrifuged at 1,000 rpm for 5 minutes. The solution was aspirated, and 6 mL medium was added into the Falcon tube to resuspend the pellet. Then, cells were plated onto pre-treated ibidi μ-slide 8 well imaging chambers for 24-48 hours prior to imaging at 37 °C in 5% CO_2 (each well should have at least $1.5*10^4$ cells). DMEM:F12 no phenol (with Mg^{2+} added) solution was made with 10.17 mg magnesium chloride hexahydrate (203 g/mol) to 50 mL DMEM:F12 no phenol. Once the cells reached 80-100%, old media was

aspirated. Cells were washed twice with 300 µL DMEM:F12 no phenol (Mg^{2+}) solution per well, 5 µM NPY was added onto well A3 and A4 and incubated in incubator for 30 minutes for competition binding studies. After aspirating solution from all wells, 300 µL DMEM:F12 no phenol solution containing one of the peptides or control solution were added into each well. Each peptide was previously diluted in the DMEM:F12 no phenol solution. The final concentration for our imaging peptides was 250 nM and for NPY was 5 µM. After incubating for 30 minutes at room temperature, the media was removed from the cell monolayer surface and each well was rinsed with 300 µL DMEM:F12 no phenol solution. The live cell images were taken with 300 µL DMEM:F12 no phenol solution in each well. A sample set up is shown in **Fig. S20**.

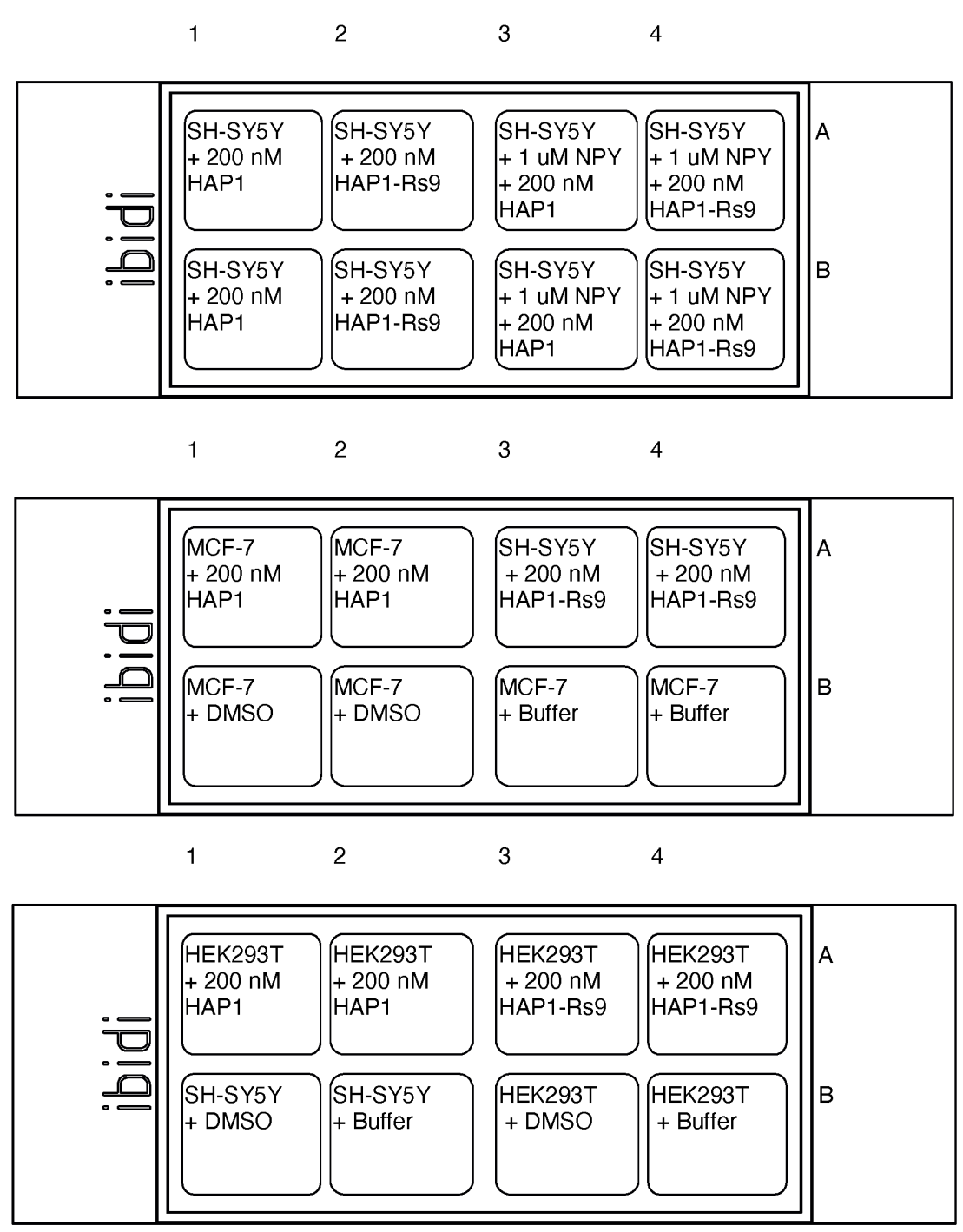

Fig. S20. An example of cellular imaging experiments with HAP1 and HAP1- R^{S}_{33} in Ibidi µ-slide 8 well imaging chambers with SH-SY5Y, MCF-7, and HEK293T cells.

For preliminary data, the cells were then immediately visualized at 20X magnification with an Olympus CKX53 microscope with a Reflected Light Fluorescence Illuminator equipped with a blue 460-495 nm exciter filler, 500 nm dichroic mirror, and a long pass 510 nm emission/barrier filter. Images were taken by Olympus EP50 camera via the Olympic EP app on an iPhone 13. All settings for the images (*e.g.* brightness, contrast, etc.) are normalized to the same values. The study was performed in one to two biological replicates (different batches of cells done on different days) (**Fig. S21**). For higher resolution data, we conducted the imaging by using an Olympus FV1000 laser scanning confocal microscope equipped with a UPLFLN 40X oil immersion objective (NA = 1.30) and a 1 Airy unit pinhole (Hoechst excitation: 405 nm; Fluorescein excitation: 488 nm) (**Figs. S22-S24**).[2] Controls with buffer (DMEM) and DMSO for each cell line are shown in **Fig. S25**. Cell fluorescence from raw images was analyzed using ImageJ-Fiji with brightness, contrast and all other settings normalized to the same value. There was no significant background fluorescence as can be seen in the buffer control.

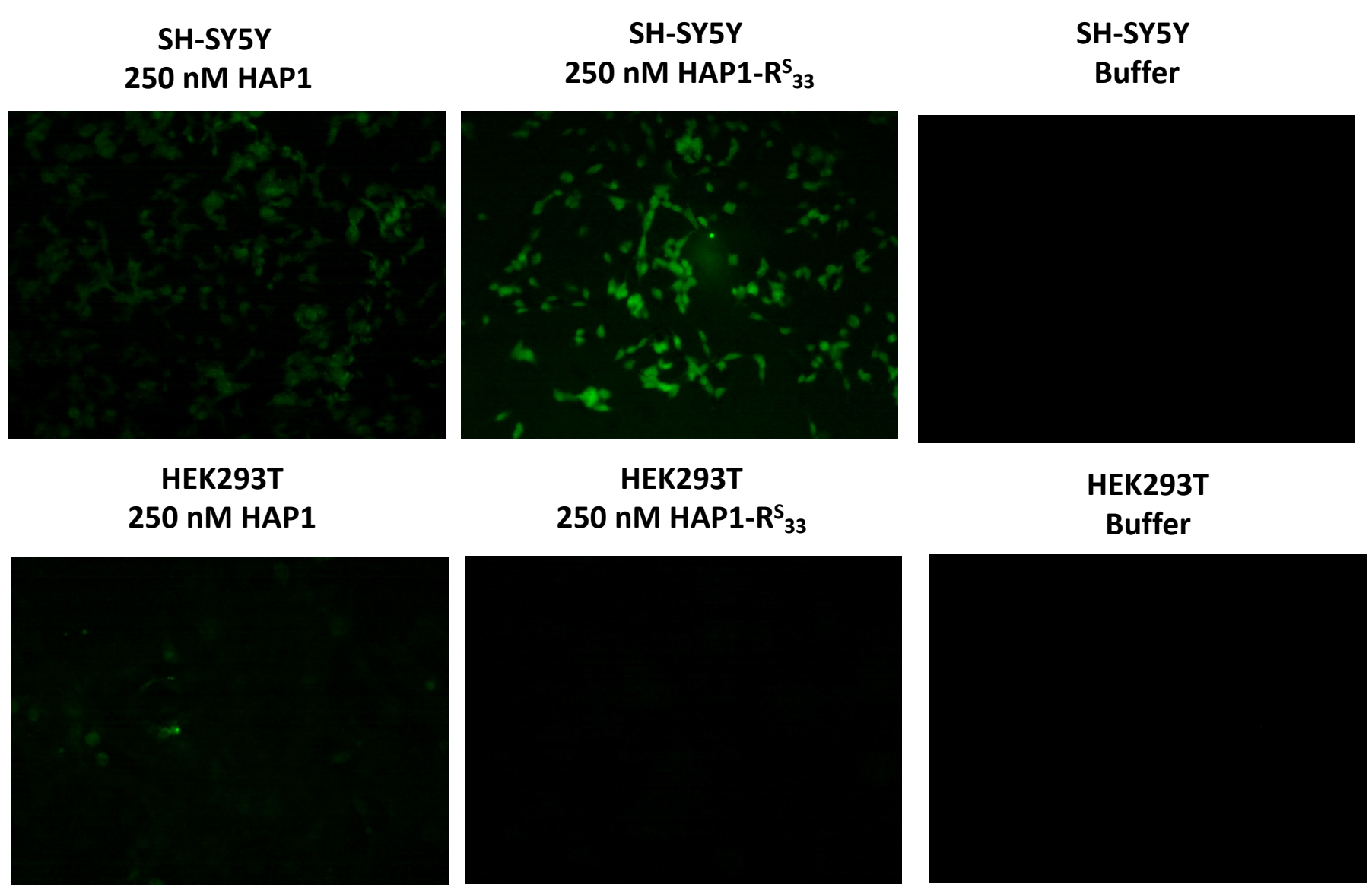

Fig. S21. Imaging neuroblastoma SH-SY5Y cells (Y_2R-expressing cells) and HEK293T cells (negative control) with HAP1 and HAP-R^S_{33} peptides.

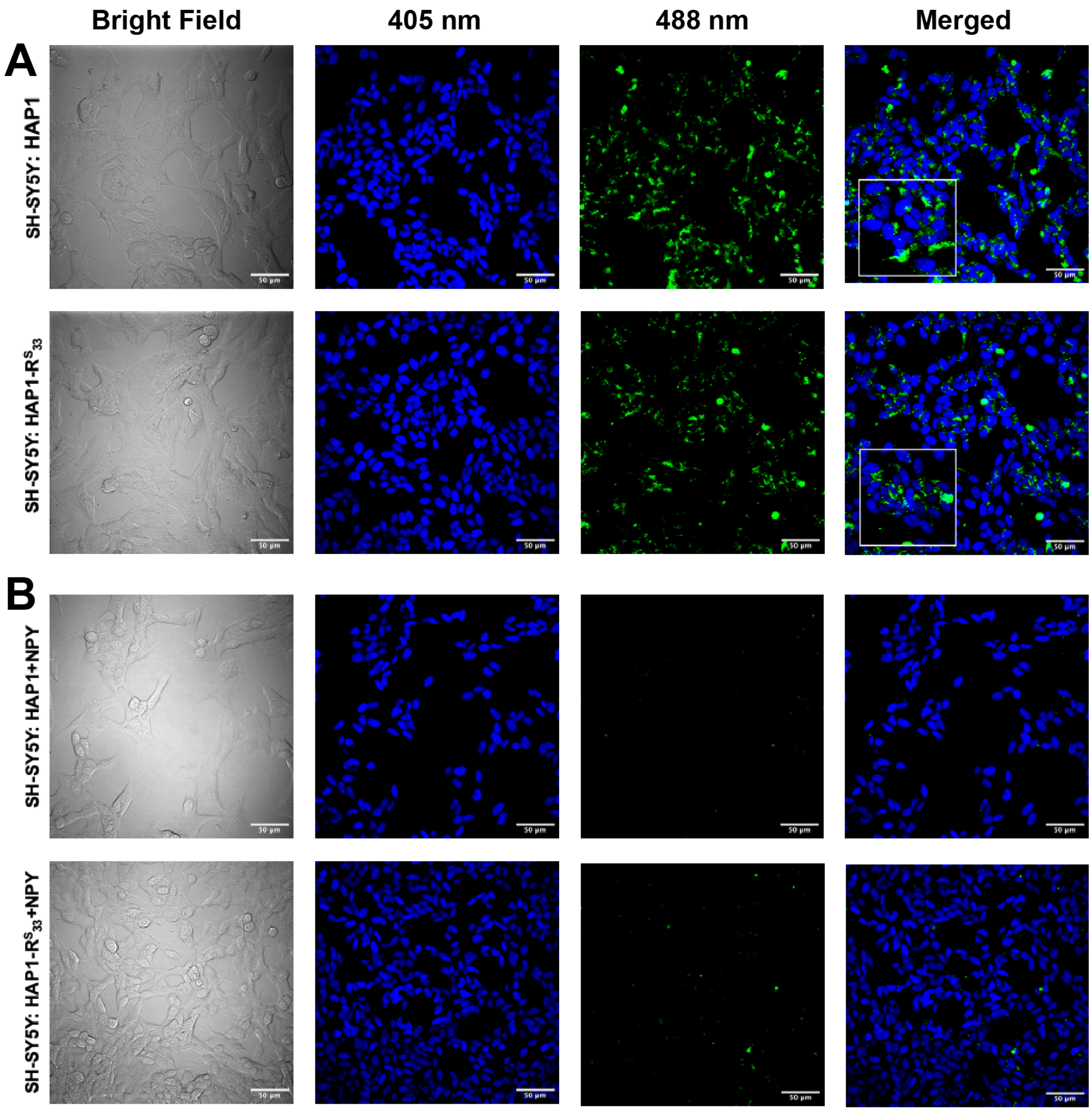

Fig. S22. Live cell imaging of Y_2R-expressing neuroblastoma SH-SY5Y cells with (A) HAP1 or HAP1-$R^S{}_{33}$ peptide and (B) HAP1 or HAP1-$R^S{}_{33}$ peptide in the presence of competing NPY peptide. Bright fields, split channel images (405 nm and 488 nm), and merged channel images are shown. Scale bar = 50 µm.

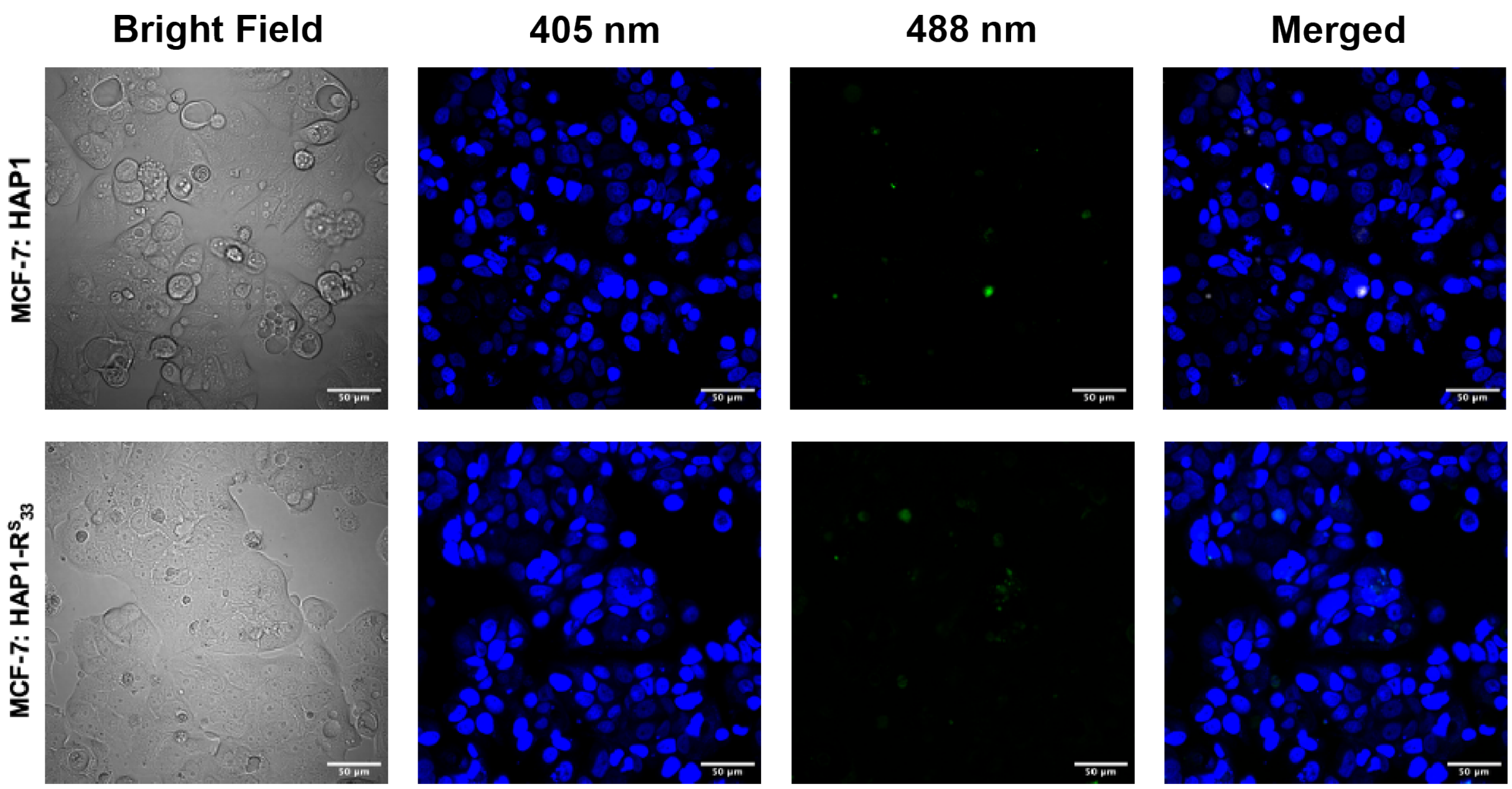

Fig. S23. Live cell imaging of Y_1R-expressing breast cancer MCF-7 cells with HAP1 or HAP1-R$^S_{33}$ peptide. Bright fields, split channel images (405 nm and 488 nm), and merged channel images are shown. Scale bar = 50 μm.

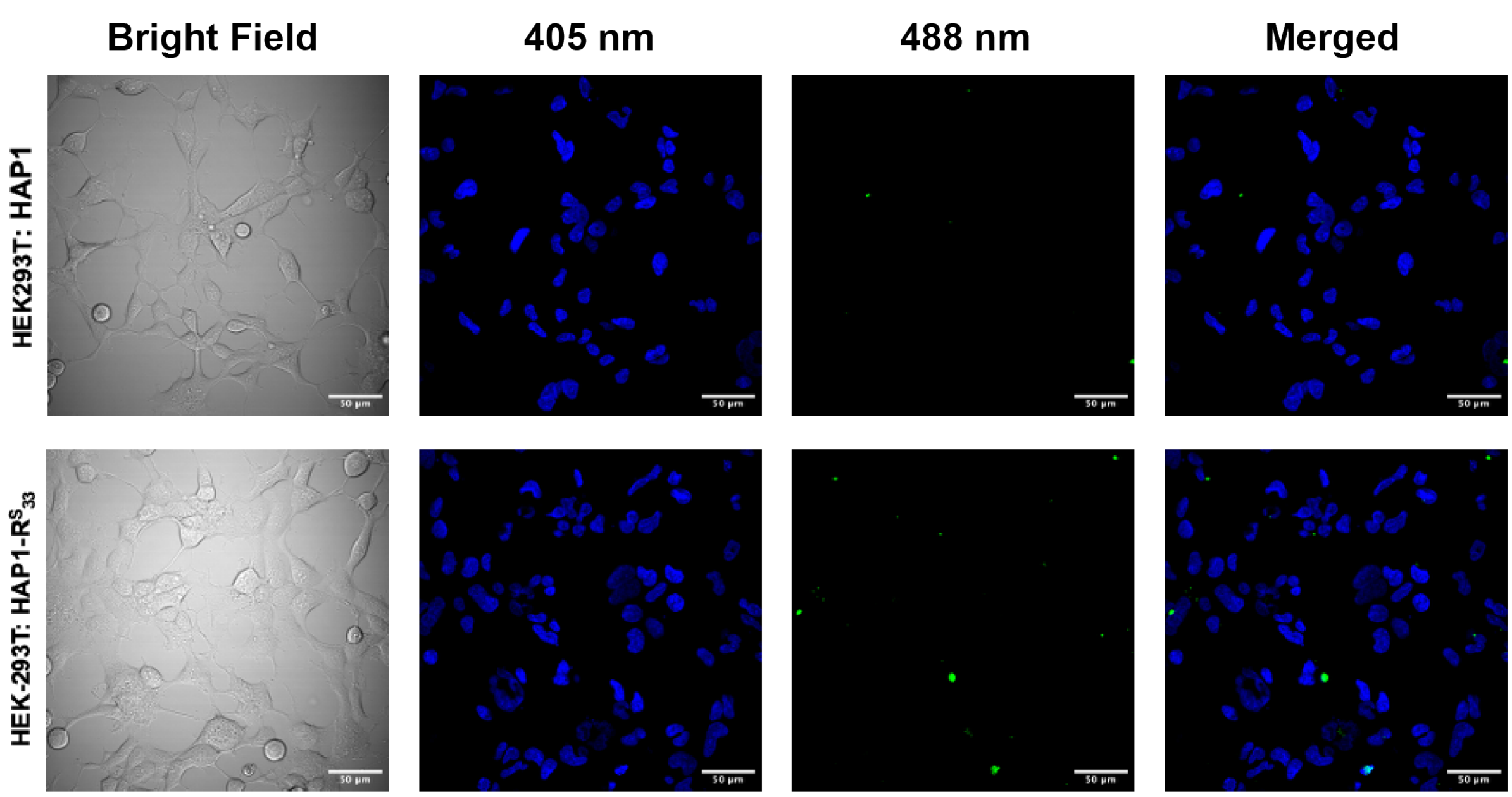

Fig. S24. Live cell imaging of NPY receptor-negative HEK293T cells with HAP1 or HAP1-R$^S_{33}$ peptide. Bright fields, split channel images (405 nm and 488 nm), and merged channel images are shown. Scale bar = 50 μm.

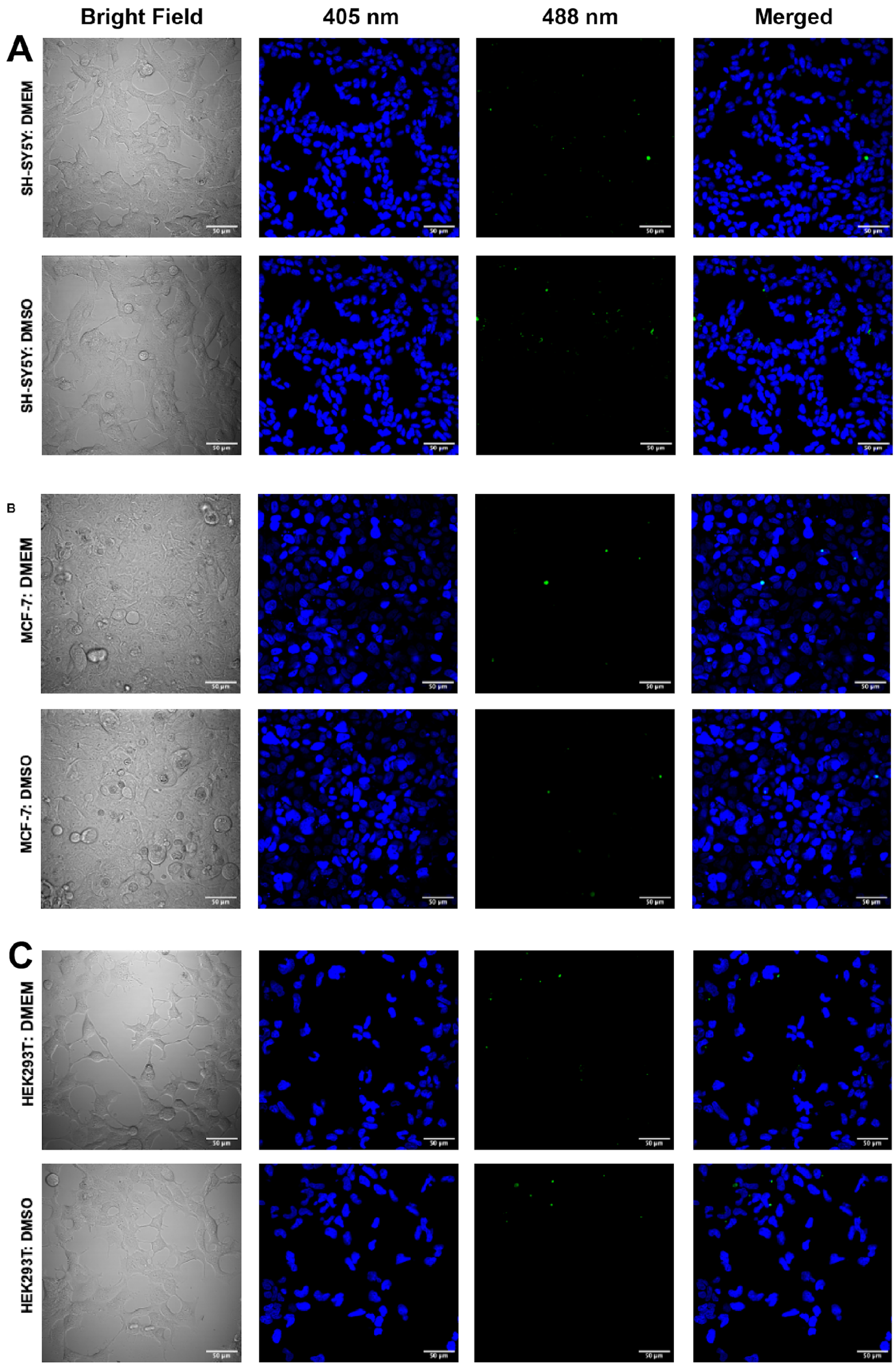

Fig. S25. Live cell imaging of buffer control (DMEM) and DMSO control for (A) neuroblastoma SH-SY5Y cells, (B) breast cancer MCF-7 cells, and (C) NPY receptor-negative HEK293T cells. Bright fields, split channel images (405 nm and 488 nm), and merged channel images are shown. Scale bar = 50 µm.

The fluorescence intensity from SH-SY5Y cells, MCF-7 cells, and HEK293T cells (**Fig. S26**) were analyzed using ImageJ-Fiji and plotted in. Using ImageJ-Fiji, the Green-channel (single-color fluorescent channel) images were quantified for integrated fluorescence density across the entire images at the same threshold. For SH-SY5Y cells, we plotted in GraphPad the average integrated density of two technical replicates from the DMSO control or from each experimental condition with HAP1 or HAP1-$R^S{}_{33}$ and either with or without the competing NPY peptides. MCF-7 and HEK293T cells were performed in singlicate.

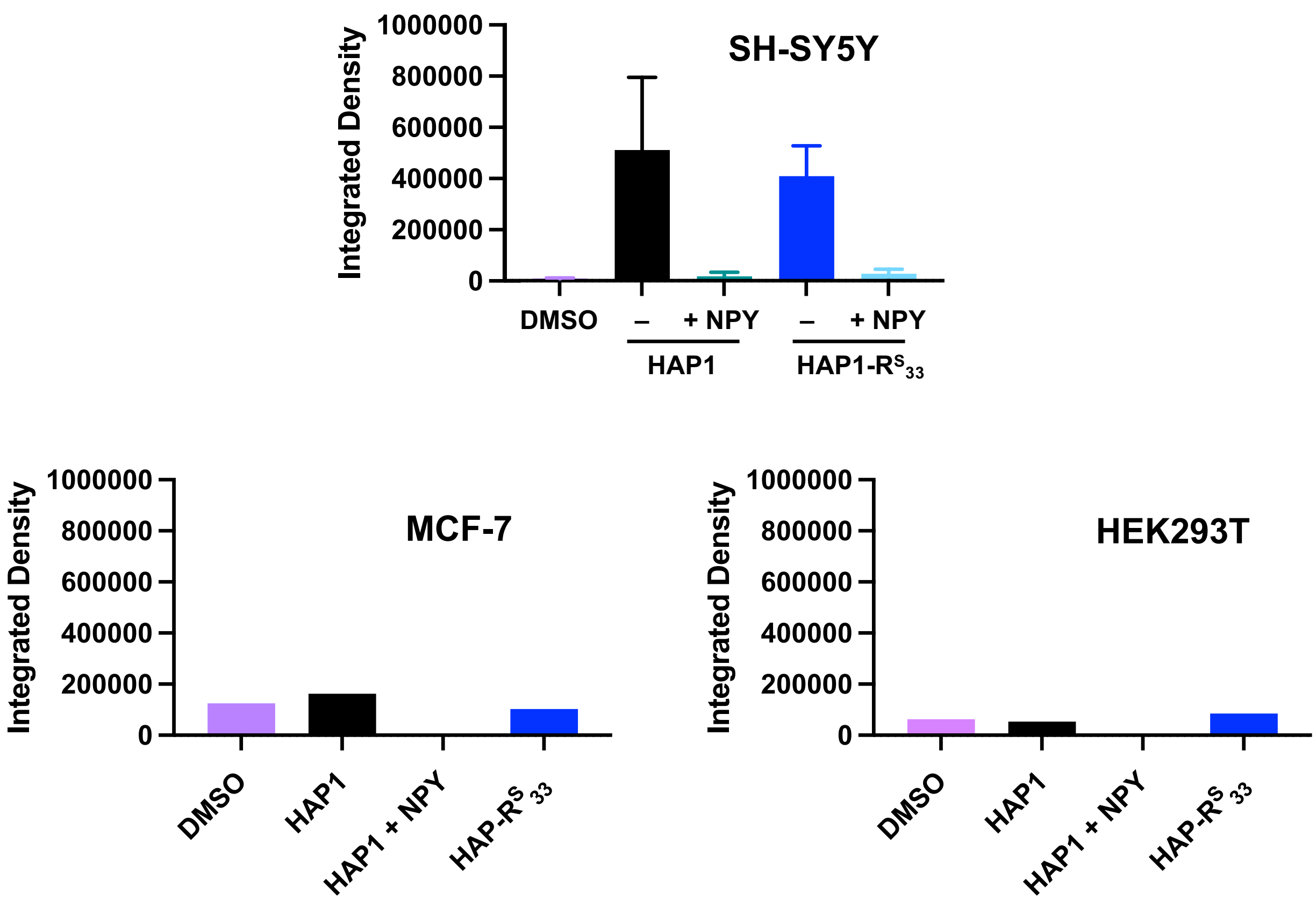

Fig. S26. Fluorescence intensity from peptide treated SH-SY5Y (Y_2R-expressing cells), MCF-7 cells (Y_1R-expressing cells) and HEK293T cells (NPY receptor negative cells). Integrated green channel fluorescence from cells treated with fluorescein-labeled HAP1 or HAP1-$R^S{}_{33}$ peptide as well as controls using DMSO vehicle only or block with excess NPY.

10. Basic Structural Characterization of NPY Y_2R Imaging Peptide

Circular Dichroism (CD)

Dried all-amide HAP1 peptide, Ac-Cyclo$_{28\text{-}32}$[$K_{25,28}E_{32}R^S{}_{33}$]NPY$_{25\text{-}36}$, was dissolved in Milli-Q water and quantified by UV based on the fluorescein extinction coefficient (ε_{494nm} = 68,000 M^{-1} cm^{-1}). Three aliquots of 200 µL of 50 µM peptides were prepared and lyophilized. Prior to the experiments, 50 µM peptide was dissolved with 200 µL of 20%, 30% and 40% TFE and adjusted to pH = 5 by adding 0.05 mM of sodium acetate. The wavelength absorbance scans were collected on a Jasco J-1500 spectrometer with a 1 mm path length Helma 110-QS CD cuvette (**Fig. S27**). Measurements were completed at 25˚C, collecting between 350-190 nm with a continuous scanning rate of 50 nm/min and a digital integration (DIT) of 1 second. The instrument was blanked with the corresponding TFE solutions before sample collection. The raw signal (θ, mDeg) was converted to molar ellipticity (θ_{Molar}), where l is the pathlength in cm and c is the concentration (M).

In 2002, Yao et al. published structural data of the cyclic NPY$_{24\text{-}36}$ scaffold in which it suggested that the peptide adopted a helical structure.[3] Our CD data showed that our fluorescently labeled construct HAP1, Ac-Cyclo$_{28\text{-}32}$[$K_{25,28}E_{32}R^S{}_{33}$]NPY$_{25\text{-}36}$, retained a helical structural, with two negative peaks at around 202 nm and 222. As 30% TFE seemed to give the most structured peptide, we used this concentration of TFE for the NMR experiment.

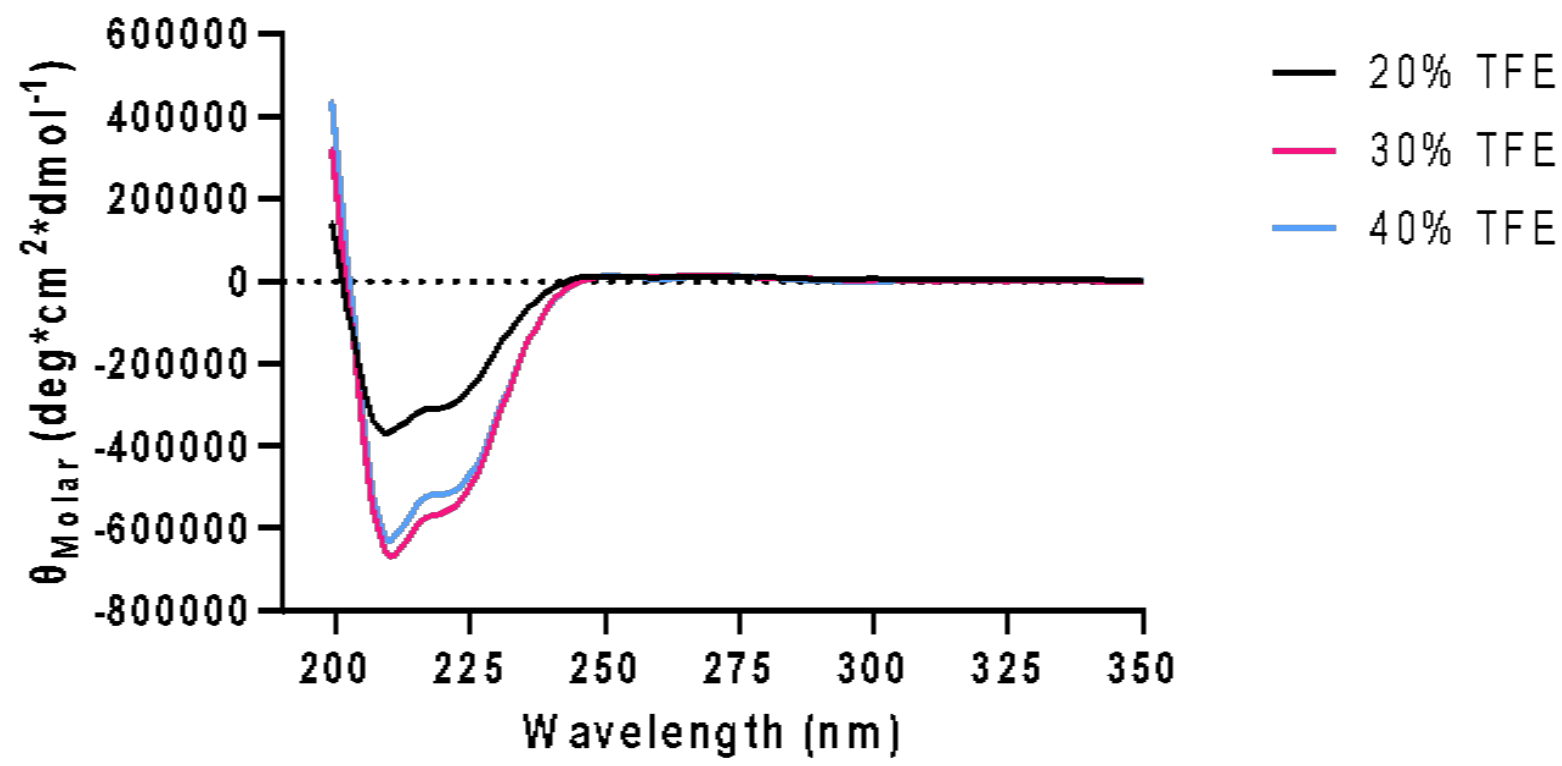

Fig. S27. CD spectra of 50 μM HAP1 peptide in TFE at pH = 5.

NMR

Using the AVANCE NEO 600 MHz NMR, we collected preliminary TOCSY (Total Correlation Spectroscopy) and Rotating-frame nuclear Overhauser effect spectroscopy (ROESY) of 3 mM all-amide HAP1 (Ac-Cyclo$_{28\text{-}32}$[K$_{25,28}$E$_{32}$R$^{S}_{33}$]NPY$_{25\text{-}36}$ construct) in 30% TFE-d$_3$, pH = 5.0 (**Fig. S28**). In the future, further analysis of the spectra along with collection of spectra of both all-amide and thioamide peptides can be collected for comparison and structural validation.

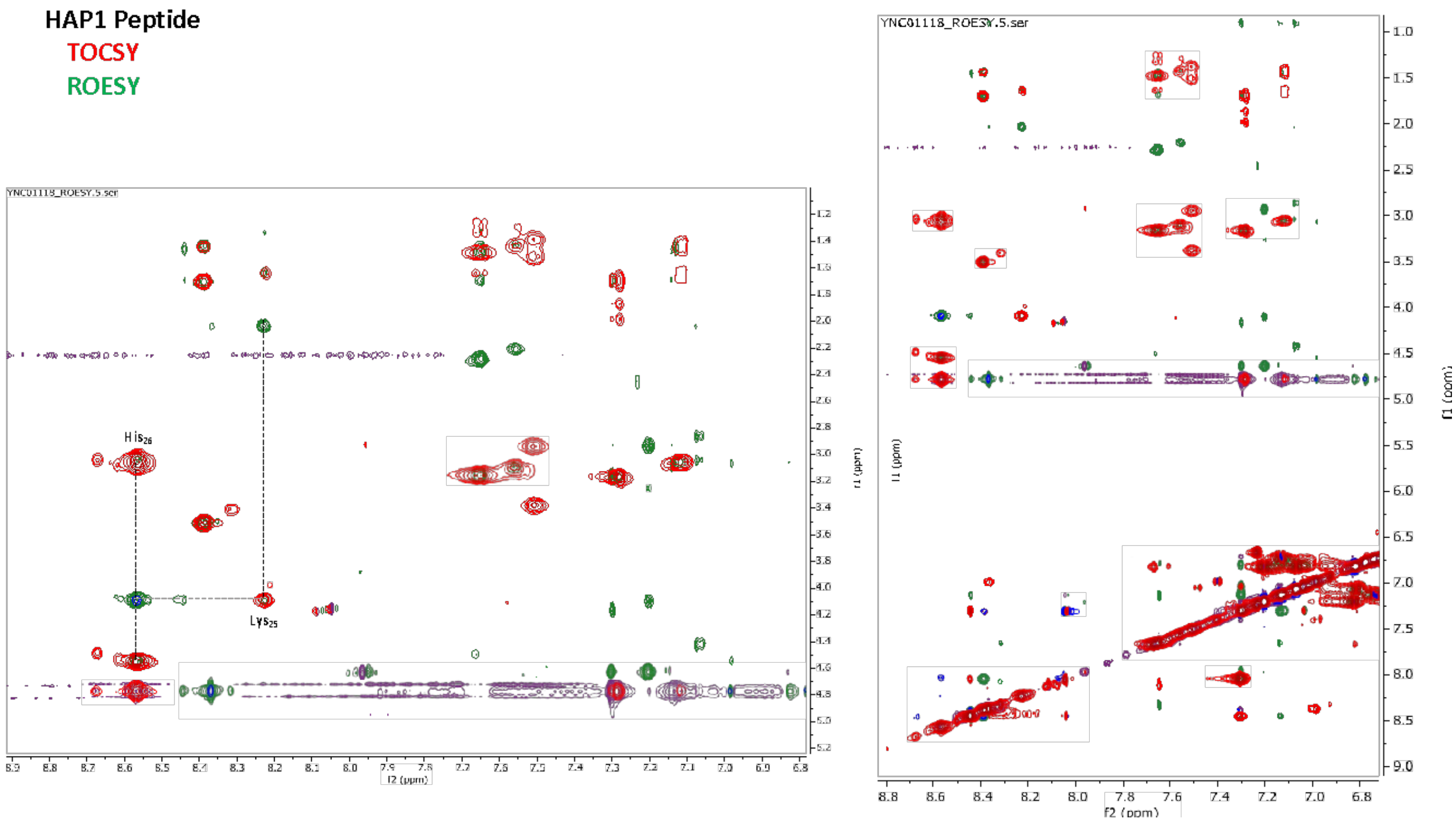

Fig. S28. TOCSY and ROESY data for the all-amide HAP1 peptide.

11. Staining of SH-SY5Y Cells with Imaging Fluorescent Peptides at 4 ºC and 37 ºC

The goal of the experiment is to quantify of fluorescein-labeled HAP1 or HAP1-R^S_{33} peptide bound to Y_2R-expressing SH-SY5Y cells. In summary, each peptide was incubated with the cells at different concentrations at either 4 ºC on ice or at 37 ºC, then quantified with flow cytometry. Standard surface marker staining is often conducted at 4 ºC degree since this prevents receptor internalization (*e.g.* endocytosis) while maintaining cell viability. However, to investigate peptide uptake or receptor internalization, incubation step can be conducted at 37 ºC or physiological temperature instead since endocytosis is not inhibited at this temperature.[4,5]

Specifically, to prepare the cells, SH-SY5Y cells were previously cultured in a T-25 flask in medium comprised of 89% DMEM:F12 (1:1), 10% fetal bovine serum, and 1% penicillin/streptomycin in 37 ºC in 5% CO_2, until they reached at least 90% confluency. To harvest the cells, the cells were first washed with DPBS without Ca^{2+} and Mg^{2+} then aspirated. Cells were then dissociated with 3 mL non-enzymatic cell lift solution (Cell Dissociation Buffer, enzyme-free, PBS; Gibco 13151014) for 15 minutes in 37 ºC in 5% CO_2 and neutralized with 10 mL medium. The mixture of cells and solution were transferred into a 15 mL Falcon tube and centrifuged at 1,500 rpm for 5 minutes. The solution was aspirated, and 1 mL medium was added into the Falcon tube to resuspend the pellet. The cells were then counted and then diluted to the appropriate volume of medium to bring the cells concentrations to 5 x 10^5 cells/mL. In a sterile 96-well plate (V-bottom plate is preferred), 100 µL of cell suspension was aliquoted into each well of a 96-well plate so that there were 5 x 10^4 cells per well. For each temperature condition, a 96-well plate was prepared.

To process the cells, the 96-well plate was centrifuged at 1500 rpm for 5 minutes before the media was removed from the cells. 100 µL of the fluorescent labeled peptide (either HAP1 or

HAP1-R^{S}_{33}) diluted in FACS buffer at different concentrations (in triplicates) was added to each well (see the concentrations and plate setup in **Fig. S29** and **Fig. S30**). The samples were mixed well by vortexting lightly (or pipetting up and down) and stained on ice at 4 ºC (Plate #1) or in the incubator at 37 ºC (Plate #2) for 30 mins. The cells were then washed 3 times with FACS buffer (1X PBS, 2.5% v/v FBS, and 0.02% sodium azide) before being centrifuged down at 1500 rpm to remove media and any extra unbound peptides. The cells were then stained for live/dead marker with 200 μL/well of 2500X TO-PRO-3 Stain (Thermo; Excitation/Emission at 642 nm/661 nm; diluted with 1X DPBS) for 10-15 minutes at room temperature and in the dark. The cells were centrifuged at 1500 rpm then the media was removed. The cells were resuspended with 200 μL/well of FACS buffer before analysis with a cytometer.

The samples were analyzed on a Beckman Coulter CytoFLEX S cytometer, using a 488 nm (Blue) laser for monitoring green emission of the fluorescent peptide, and the 630/640 (Red) laser for monitoring the nuclear stain or dead cell indicator. Regarding the gating strategy, a sample of gating was shown in **Fig. S31**. Forward and side scatters (SSC-A vs FSC-A) was first used to exclude debris and select the overall population of SH-SY5Y cells (labeled as "Cells"). SSC-H (height) vs. SSC-A (area) gating was then performed to remove doublets and select only singlets for analysis (labeled as "Single Cells"). Only live cells were selected for further analysis (labeled as "Live Single Cells") using the channel detecting TO-PRO-3 stain (APC channel). To detect positive staining with the green fluorescent peptides (labeled as "Live Single Cells, FAM+), gating was set against the unstained cell controls (0 nM of peptide); there was good separation in fluorescent signal between negative and positive staining populations (**Fig. S32**).

	1	2	3	4	5	6	7	8	9	10	11	12
A	16000.0	8000.0	4000.0	2000.0	1000.0	500.0	250.0	0.0	HAP1 (Oxo)			
B	16000.0	8000.0	4000.0	2000.0	1000.0	500.0	250.0	0.0	HAP1 (Oxo)			
C	16000.0	8000.0	4000.0	2000.0	1000.0	500.0	250.0	0.0	HAP1 (Oxo)			
D	16000.0	8000.0	4000.0	2000.0	1000.0	500.0	250.0	0.0	HAP1-R^S_{33} (Thio)			
E	16000.0	8000.0	4000.0	2000.0	1000.0	500.0	250.0	0.0	HAP1-R^S_{33} (Thio)			
F	16000.0	8000.0	4000.0	2000.0	1000.0	500.0	250.0	0.0	HAP1-R^S_{33} (Thio)			
G												
H												

Fig. S29. An example of a plate setup for staining SH-SY5Y cells with HAP1 or HAP1-R^S_{33} at 4 ºC. The peptide concentrations are reported in nM.

	1	2	3	4	5	6	7	8	9	10	11	12
A	8000.0	4000.0	2000.0	1000.0	500.0	250.0	125.0	0.0	HAP1 (Oxo)			
B	8000.0	4000.0	2000.0	1000.0	500.0	250.0	125.0	0.0	HAP1 (Oxo)			
C	8000.0	4000.0	2000.0	1000.0	500.0	250.0	125.0	0.0	HAP1 (Oxo)			
D	8000.0	4000.0	2000.0	1000.0	500.0	250.0	125.0	0.0	HAP1-R^S_{33} (Thio)			
E	8000.0	4000.0	2000.0	1000.0	500.0	250.0	125.0	0.0	HAP1-R^S_{33} (Thio)			
F	8000.0	4000.0	2000.0	1000.0	500.0	250.0	125.0	0.0	HAP1-R^S_{33} (Thio)			
G												
H												

Fig. S30. An example of a plate setup for staining SH-SY5Y cells with HAP1 or HAP1-R^S_{33} at 37 ºC. The peptide concentrations are reported in nM.

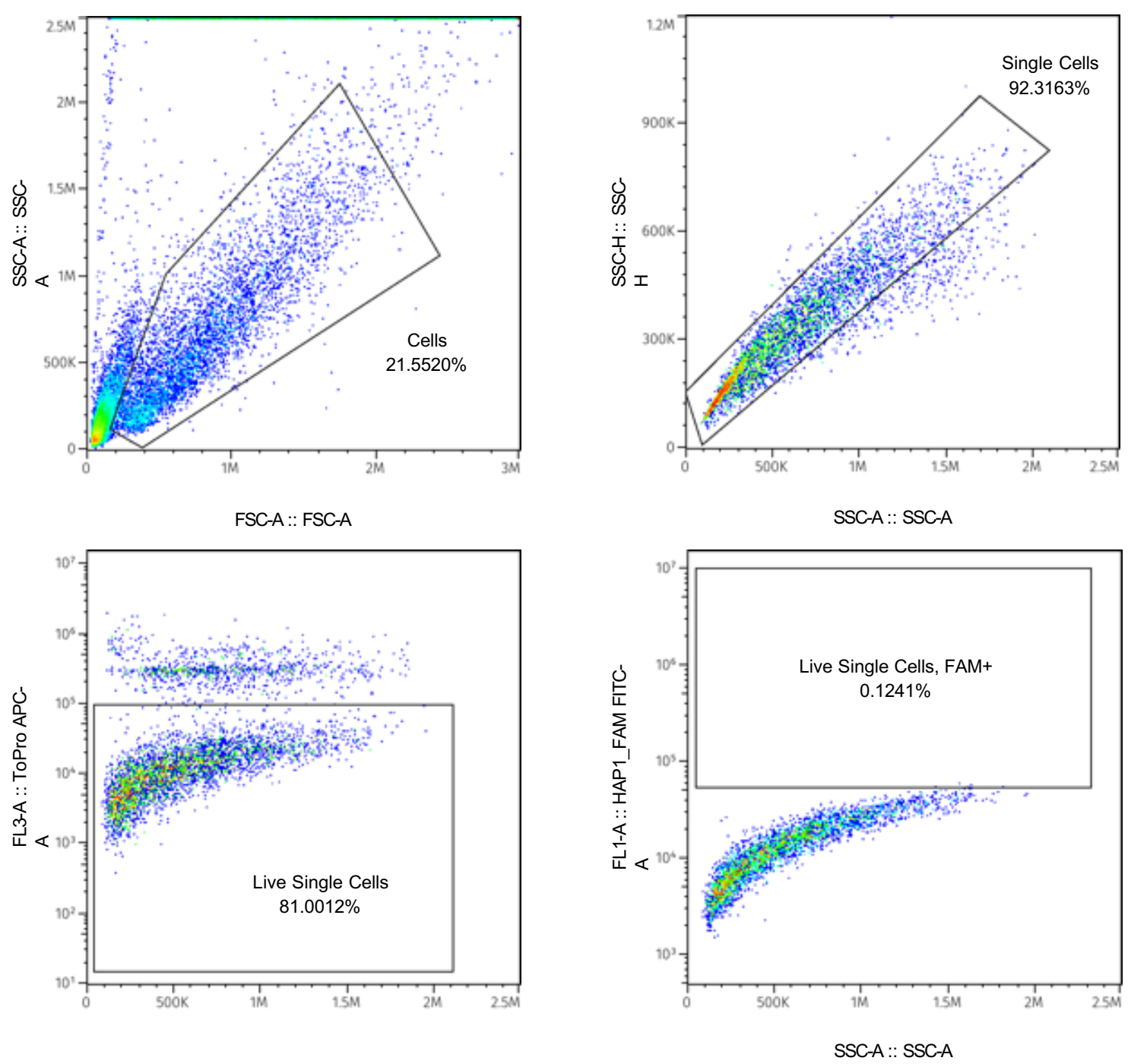

Fig. S31. An example of gating strategy for SH-SY5Y cells (unstained or 0 nM HAP1 peptide).

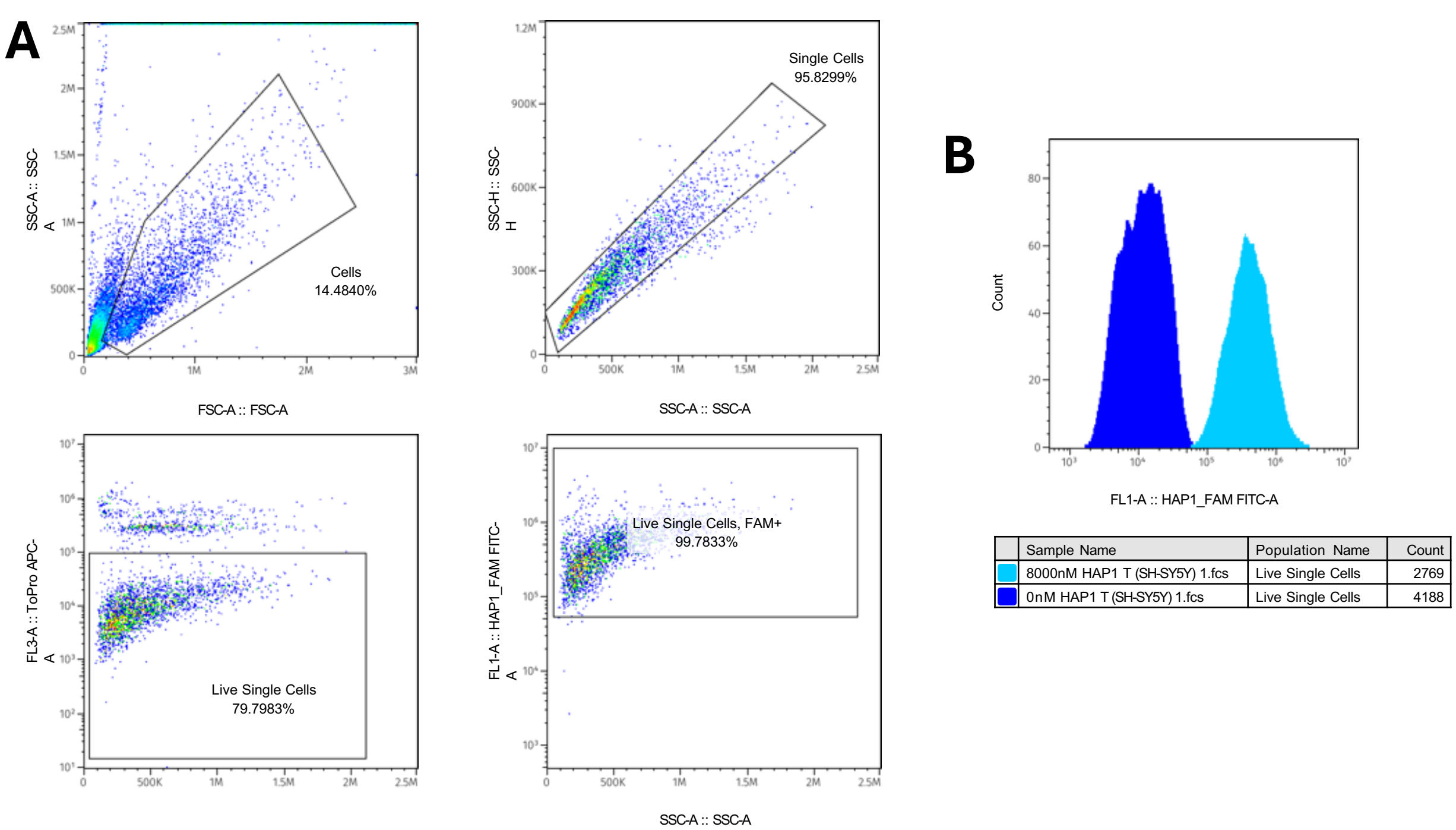

Fig. S32. An example of how the gating in **Fig. S31**. allowed identification of the positively stained SH-SY5Y cells with HAP1-R^{S}_{33} at 37 ºC (**A**). The histogram (**B**) showed that the positively stained cells population showed a good fluorescence signal separation compared to the unstained population, as evident in a clear right shift of the MFI (Median Fluorescence Intensity).

The cytometry data was analyzed with FlowJo software. For each temperature condition and each peptide, the percent of Live Single Cells stained positive for fluorescent peptides at each peptide concentration was plotted and fitted to the "One Site – Total" Binding Saturation model in Prism to obtain K_d values and standard errors.

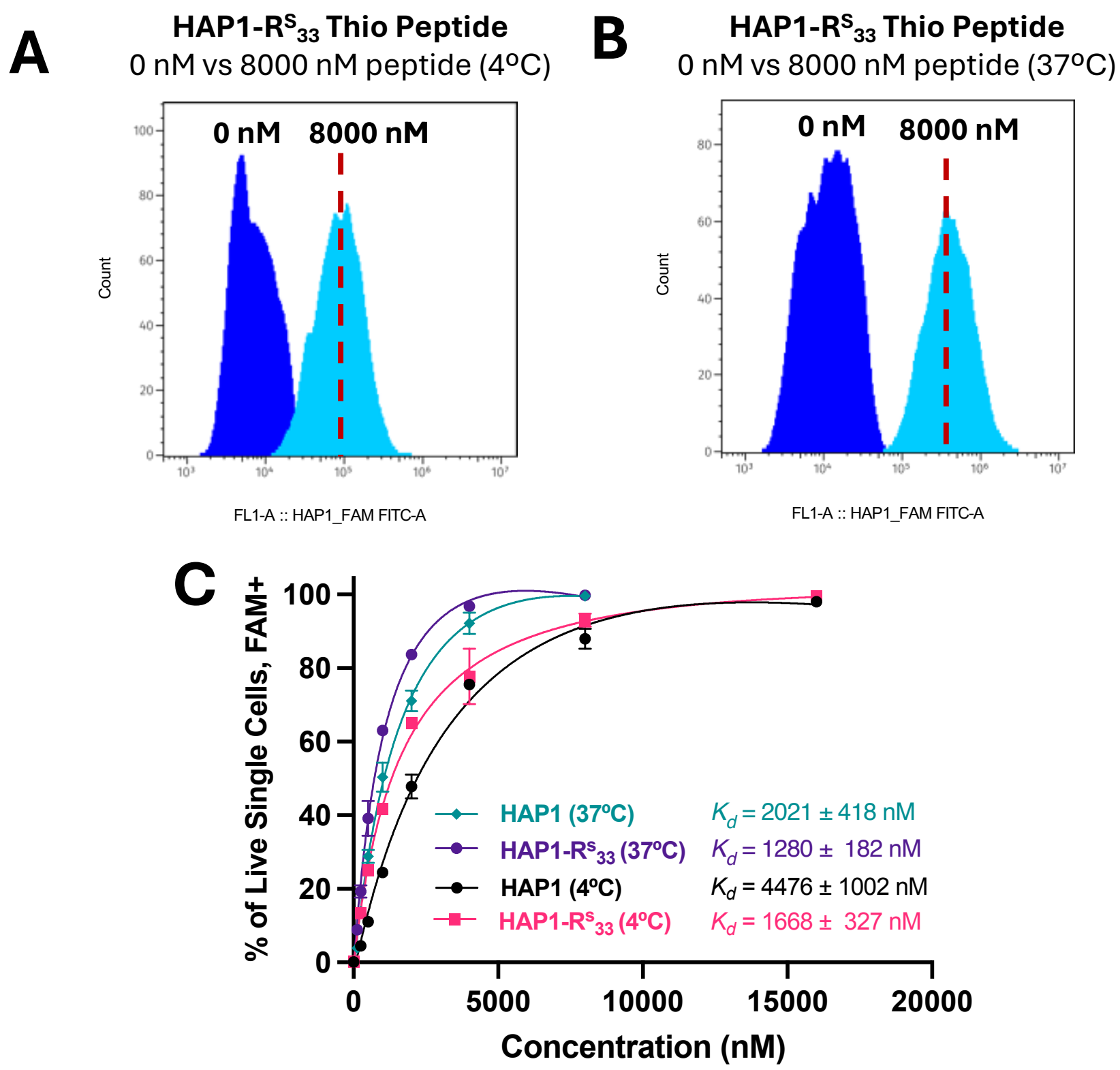

Fig. S33. Staining of HAP1 or HAP1-R$^S_{33}$ peptide with Y_2R-expressing SH-SY5Y cells at 4 ºC versus 37 ºC staining temperature (**C**). Representative histograms of green fluorescence channel of unstained cells vs 8000 nM HAP1-R$^S_{33}$ peptide at 4 ºC (**A**) vs. 37 ºC (**B**) are shown.

Table S6. A summary of the binding affinity between HAP1 or HAP1-R$^S_{33}$ peptide and Y_2R-expressing SH-SY5Y cells at 4ºC and 37ºC incubation or staining temperature. The K_d value is shown for each condition along with the standard error.

Peptide	4 ºC Incubation	37 ºC Incubation
HAP1	4476 ± 1002 nM	2021 ± 418 nM
HAP1-R$^S_{33}$	1668 ± 327 nM	1280 ± 182 nM

12. Imaging Temperature-Dependent Internalization of Peptides by SH-SY5Y Cells

The goal of the experiment was to visualize fluorescein-labeled HAP1 or HAP1-R^{S}_{33} peptide internalized by Y_2R-expressing SH-SY5Y cells. The following media were used in cell culture: DMEM:F12 (Corning), DMEM:F12 no phenol (Thermo Fisher), 10% FBS (Atlanta Biologicals), 1% Glutamine Supplement (Gibco), 1% Penicillin/Streptomycin (Gibco). SH-SY5Y cells were cultured in the media in a T25 flask prior to the experiment. SH-SY5Y cells were cultured in 89% DMEM:F12 (1:1), 10% fetal bovine serum, and 1% Penicillin/Streptomycin. For the cell imaging experiment, 25 µg/mL poly-D-lysine solution was premade with sterile water (tissue culture grade; CCS 1180) and poly-D-lysine hydrobromide (Sigma P6407). The culture surface of ibidi µ- slide 8 well imaging chambers was aseptically coated with 300 µL of the poly-D-lysine solution (300 µL /well or per 25 cm^2) and incubated for 1 hour at room temperature. The solution was aspirated, and each well was rinsed with sterile tissue culture grade sterile water 3 times as excess poly-D-lysine could be toxic to the cells. The coated vessel was uncovered in the laminal hood to dry for at least 2 hours before introducing cells and medium.

When cells reached 80-90% confluency in a T25 flask, the medium was aspirated. Cells were washed with DPBS without Ca^{2+} and Mg^{2+} then aspirated. Cells were then trypsinized with 3 mL trypsin-EDTA for 5 minutes and neutralized with 10 mL medium. The mixture of cells and solution were transferred into a 15 mL falcon tube and centrifuged at 1,000 rpm for 5 minutes. The solution was aspirated, and 6 mL medium was added into the Falcon tube to resuspend the pellet. Then, cells were plated onto pre-treated ibidi µ-slide 8 well imaging chambers for 24-48 hours prior to imaging at 37 °C in 5% CO_2 (each well should have at least $1.5*10^4$ cells). DMEM:F12 no phenol (with Mg^{2+} added) solution was made with 10.17 mg magnesium chloride hexahydrate (203 g/mol) to 50 mL DMEM:F12 no phenol. Once the cells reached 80-100%, old media was aspirated. Cells

were washed twice with 300 µL DMEM:F12 no phenol (Mg^{2+}) solution per well. Then, the cells were stained with 0.5µg/mL Hoechst 33342 and 1.25µg/mL Wheat Germ Agglutinin conjugated with Alexa Fluor Plus 568 for 5 minutes at room temperature. Cells were washed twice with 300 µL DMEM:F12 no phenol (Mg^{2+}) solution per well. After aspirating solution from all wells, 300 µL DMEM:F12 no phenol solution containing one of the peptides (HAP1 or HAP1-R^{S}_{33} peptide) or control solution were added into each well. Each peptide was previously diluted in the DMEM:F12 no phenol solution. The final concentration for our imaging peptides was 250 nM. After incubating for 30 minutes at 4 ºC or 37 ºC, the media was removed from the cell monolayer surface and each well was rinsed with 300 µL DMEM:F12 no phenol solution. The live cell images were taken with 300 µL DMEM:F12 no phenol solution in each well.

We conducted the imaging by using a Zeiss LSM 980 laser scanning confocal microscope equipped with a 63X oil immersion objective (NA = 1.4) and a 1 Airy unit pinhole (Hoechst excitation: 405 nm; Fluorescein excitation: 488 nm; Alexa Fluor Plus 568 excitation: 561nm). The images at 63X are shown in **Figs. S34-S39**; the images for the bright fields and the images at the 405 nm, 488 nm, 561 nm channels along with the merged images of the 3 channels are shown.

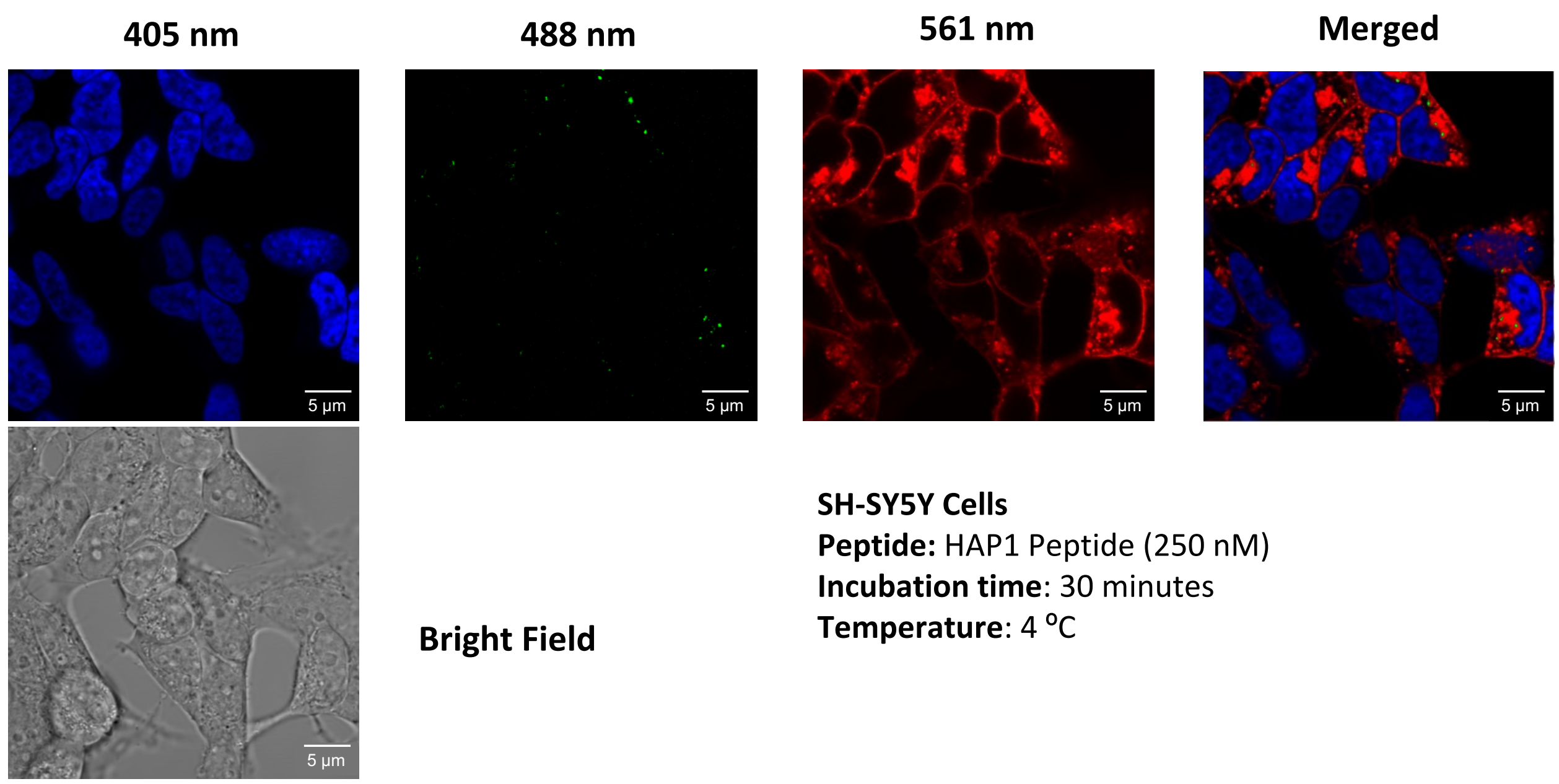

Fig. S34. Images of SH-SY5Y cells incubated with HAP1 peptide for 30 minutes at 4 ºC.

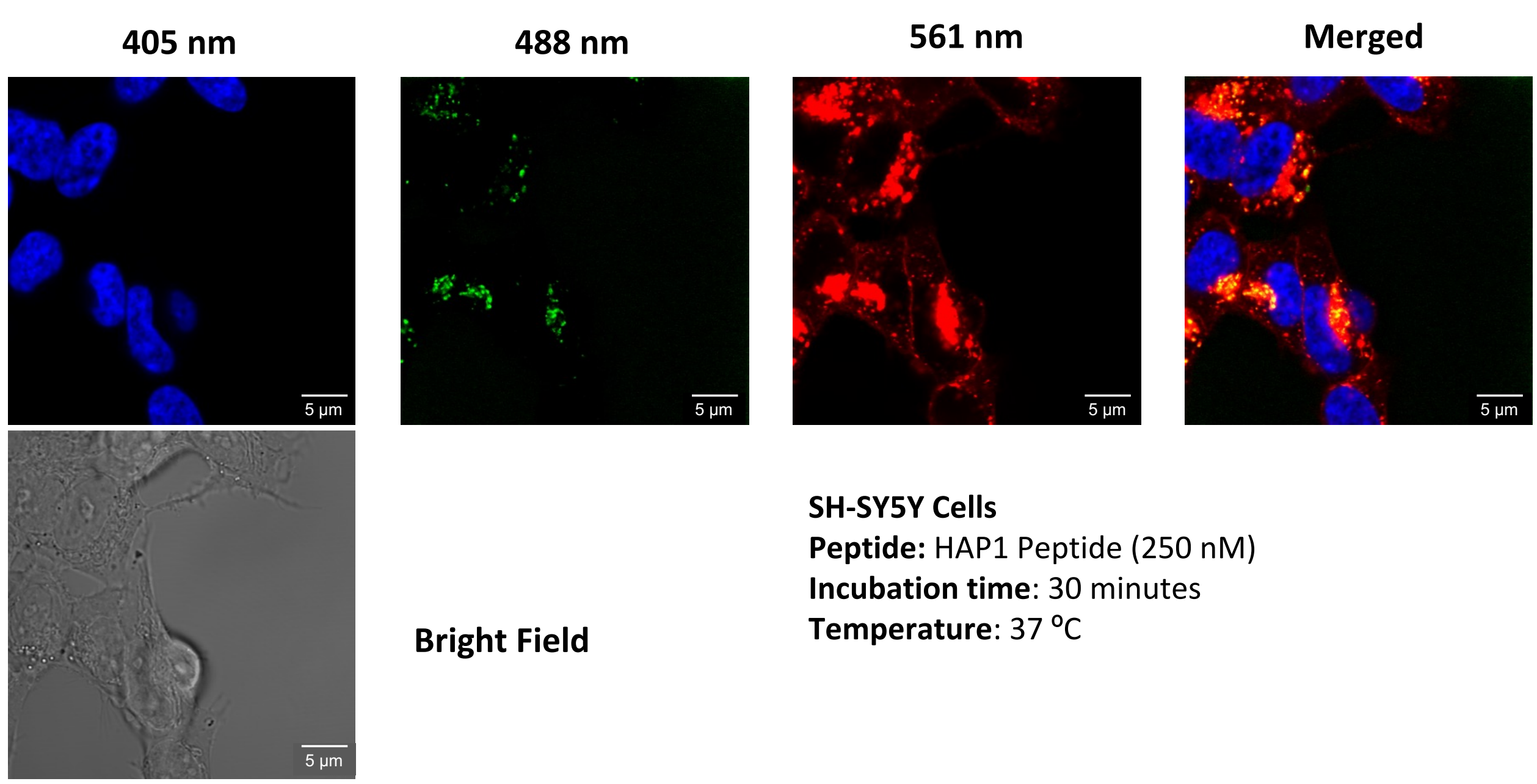

Fig. S35. Images of SH-SY5Y cells incubated with HAP1 peptide for 30 minutes at 37 ºC.

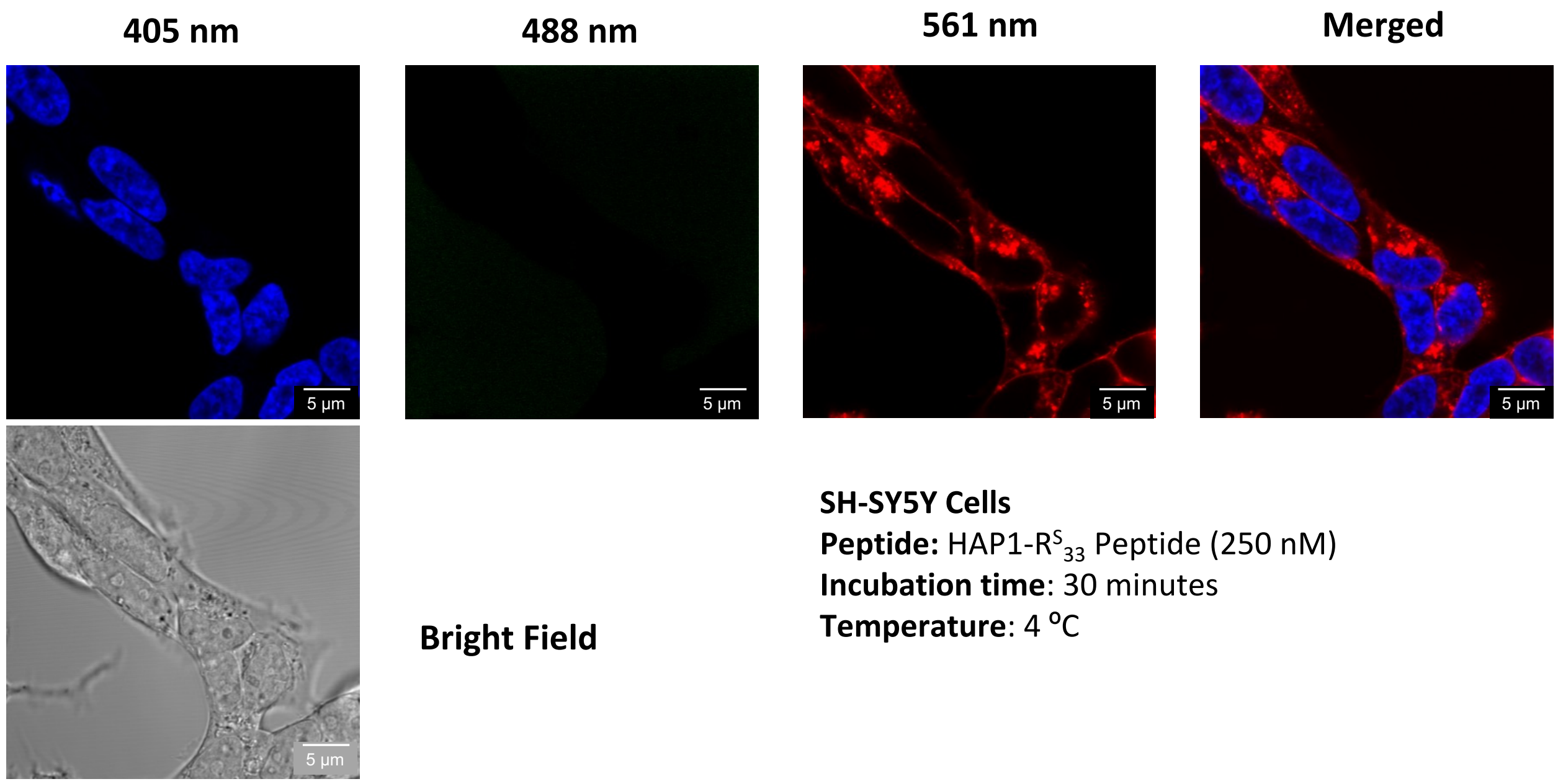

Fig. S36. Images of SH-SY5Y cells incubated with HAP1-R^S_{33} peptide for 30 minutes at 4 °C.

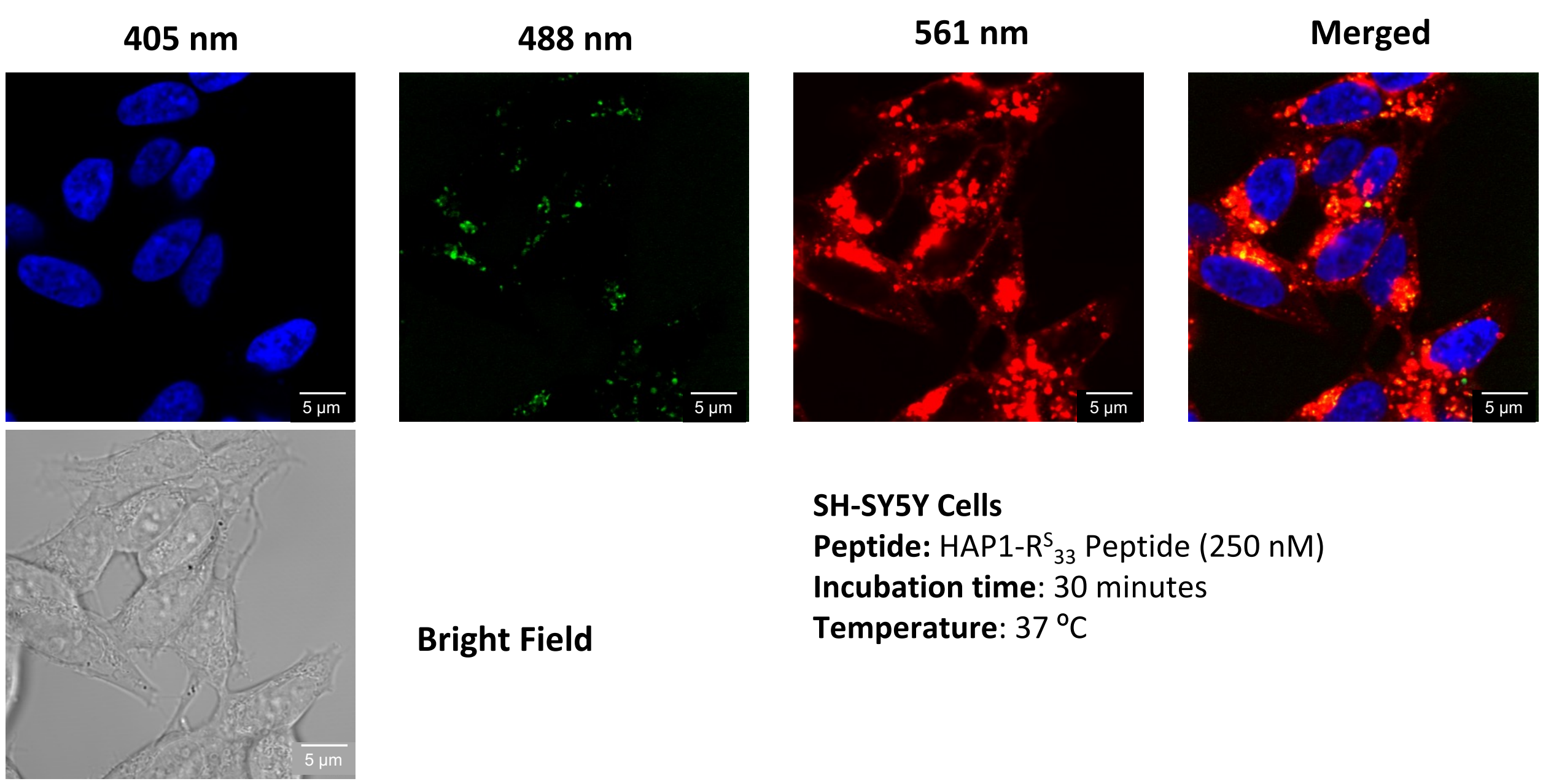

Fig. S37. Images of SH-SY5Y cells incubated with HAP1-R^S_{33} peptide for 30 minutes at 37 °C.

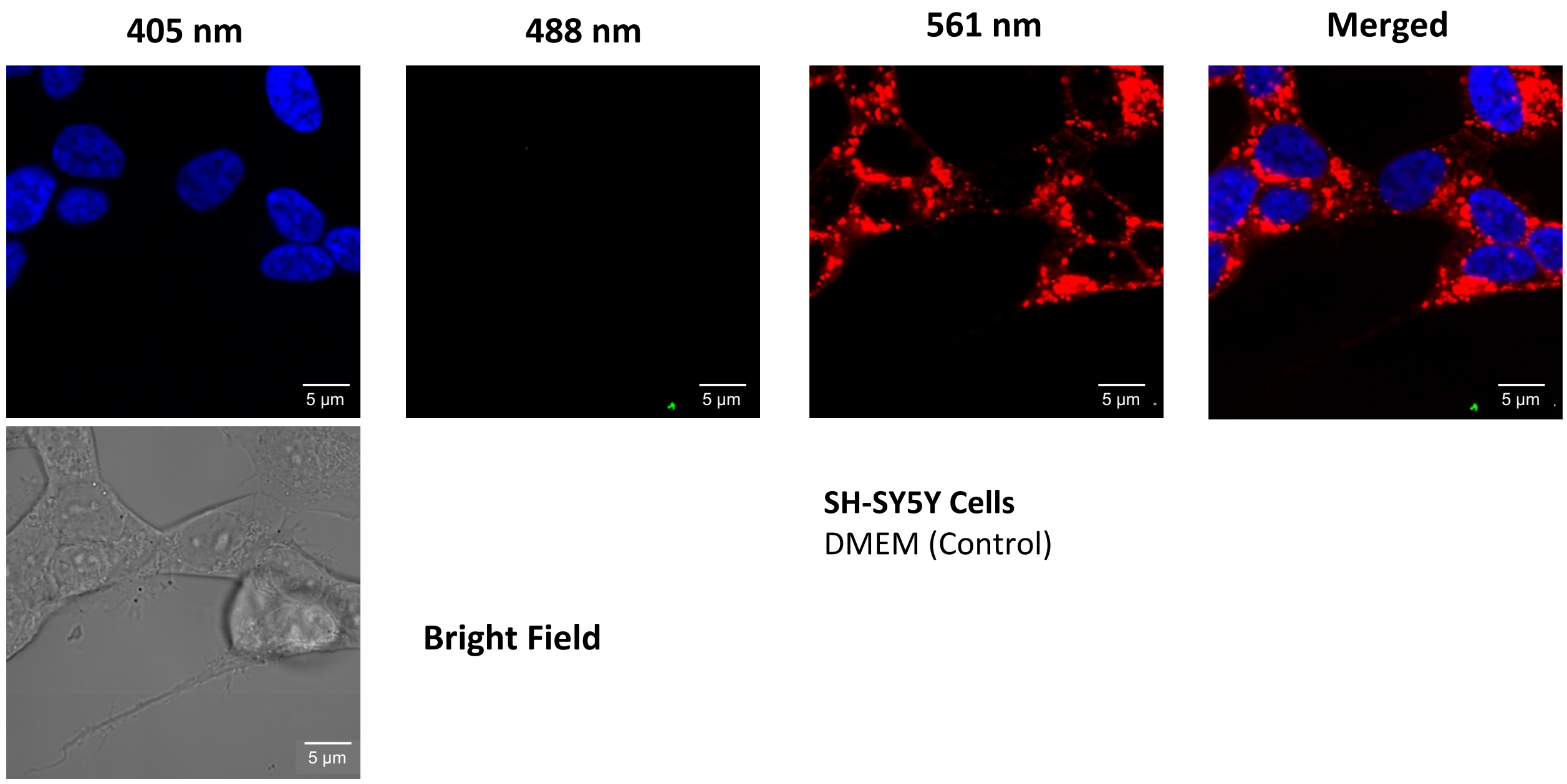

Fig. S38. Images of SH-SY5Y cells incubated with DMEM (media control).

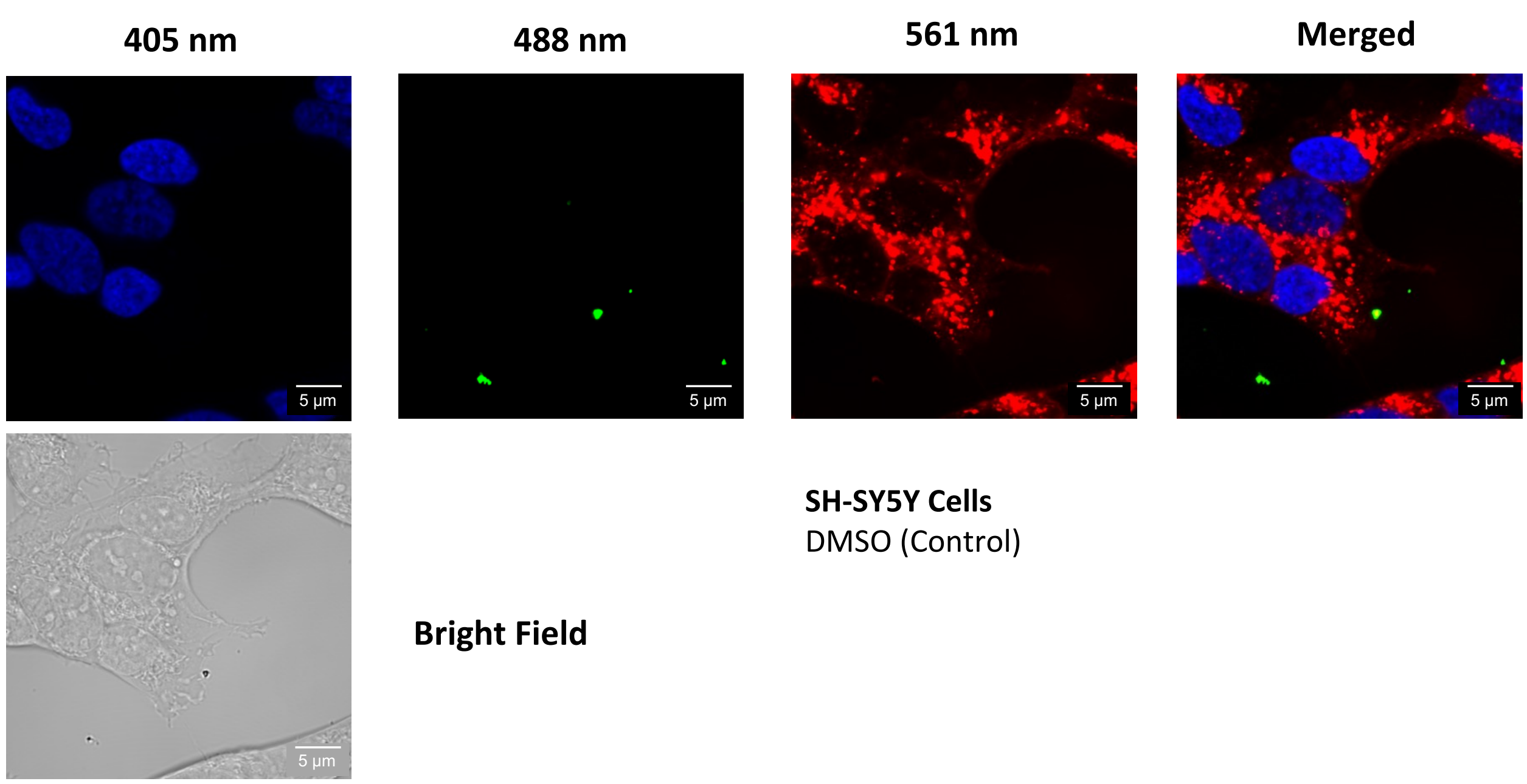

Fig. S39. Images of SH-SY5Y cells incubated with DMSO (control).

13. References

(1) Barrett, T. M.; Chen, X. S.; Liu, C.; Giannakoulias, S.; Phan, H. A. T.; Wang, J.; Keenan, E. K.; Karpowicz, R. J.; Petersson, E. J. Studies of Thioamide Effects on Serine Protease Activity Enable Two-Site Stabilization of Cancer Imaging Peptides. *ACS Chem. Biol.* **2020**, *15* (3), 774–779. https://doi.org/10.1021/acschembio.9b01036.

(2) Herling, M. R.; Dmochowski, I. J. Ratiometric, pH-Sensitive Probe for Monitoring siRNA Delivery. *J. Am. Chem. Soc.* **2023**, *145* (17), 9417–9422. https://doi.org/10.1021/jacs.3c01032.

(3) Yao, S.; Smith-White, M. A.; Potter, E. K.; Norton, R. S. Stabilization of the Helical Structure of Y2-Selective Analogues of Neuropeptide Y by Lactam Bridges. *J. Med. Chem.* **2002**, *45* (11), 2310–2318. https://doi.org/10.1021/jm010543z.

(4) Nakase, I.; Niwa, M.; Takeuchi, T.; Sonomura, K.; Kawabata, N.; Koike, Y.; Takehashi, M.; Tanaka, S.; Ueda, K.; Simpson, J. C.; Jones, A. T.; Sugiura, Y.; Futaki, S. Cellular Uptake of Arginine-Rich Peptides: Roles for Macropinocytosis and Actin Rearrangement. *Molecular Therapy* **2004**, *10* (6), 1011–1022. https://doi.org/10.1016/j.ymthe.2004.08.010.

(5) Yousef, M.; Szabó, I.; Murányi, J.; Illien, F.; Soltész, D.; Bató, C.; Tóth, G.; Batta, G.; Nagy, P.; Sagan, S.; Bánóczi, Z. Cell-Penetrating Dabcyl-Containing Tetraarginines with Backbone Aromatics as Uptake Enhancers. *Pharmaceutics* **2023**, *15* (1), 141. https://doi.org/10.3390/pharmaceutics15010141.
